# Long‐Lasting Cross‐Linked PLGA‐Inspired Nanoparticles from One‐Pot Nanopolymerization of Precisely Sequenced Short Oligolactoglycolic Acid Dimethacrylates

**DOI:** 10.1002/marc.202400778

**Published:** 2025-01-21

**Authors:** Luka Blagojevic, Nazila Kamaly

**Affiliations:** ^1^ Department of Chemistry Imperial College London Molecular Sciences Research Hub White City Campus, Wood Lane London W12 0BZ UK

**Keywords:** drug delivery, nanoparticle, nanopolymerization, oligoester, PLGA

## Abstract

A novel PLGA‐inspired NP polymerization technique is presented, which allows the formation of NPs via the cross‐linking of precisely sequenced short oligolactoglycolic acid dimethacrylates (OLGADMAs). Following the synthesis of a range of OLGADMAs, a library of NPs via this rapid and surfactant‐free nanopolymerization method is successfully generated, which permits the simultaneous NP formation and encapsulation of drugs such as dexamethasone. The results indicate that NPs produced through this nanopolymerization technique with precisely controlled sequences exhibit heightened stability compared to conventionally sequenced and non‐sequence controlled PLGA, as evidenced by minimal pH changes over five weeks. This improved stability is attributed to simultaneous crosslinking and co‐polymerization of the OLGADMAs. Moreover, the long‐acting NPs demonstrate minimal cytotoxicity and uniform cellular uptake in vitro. It is concluded that the ability to precisely regulate the sequence of short PLGA‐inspired monomers and employ a unique in situ nanopolymerizing reaction results in exceptionally stable NPs for sustained drug delivery and opens exciting possibilities for the development of a range of long‐lasting drug delivery systems with programmable structure and function.

## Graphical abstract

PLGA‐inspired monomers form highly stable polymeric nanoparticles via a rapid process that allows for simultaneous radical polymerization and nanoparticle formation. Small‐molecule drugs such as dexamethasone undergo a facile in situ encapsulation which occurs with above‐average drug encapsulation efficiencies. The novel synthetic approach yields crosslinked polymeric nanoparticles with desirable drug delivery properties and bears the potential for enabling novel classes of precision nanoparticles.

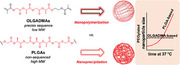

## 1 Introduction

The use of nanoparticles (NPs) as drug carriers can enhance the therapeutic efficacy, minimize the toxicity of established drugs, enable new classes of therapeutics, and allow for the re‐evaluation of bioactive molecules bearing suboptimal pharmaceutical properties.^[^ ^1^ ^]^ Lipid‐based, inorganic, and polymeric NPs are being engineered in increasingly specific ways, facilitating the advancement of nanocarriers toward refined biomedical applications (e.g., precision medicine).^[^ ^2^ ^]^ Polymeric NPs have been described as ideal candidates for drug delivery as they can be designed to possess a range of desirable properties including biocompatibility, biodegradability, and stability upon storage.^[^ ^2^ ^]^ Polymeric NPs can be made of natural or synthetic polymers and obtained from preformed polymers or monomers. A traditional method for the preparation of NPs from preformed polymers relies on the nanoprecipitation method developed by Fessi and co‐workers.^[^ ^3^ ^]^ The method typically involves the addition of a hydrophobic polymer dissolved in a water‐miscible organic solvent to an aqueous phase, which results in the formation of dispersed nanodroplets. The subsequent elimination of the organic solvent yields an aqueous NP suspension. NPs are also obtainable via polymerization of smaller building blocks such as monomers and crosslinkers, allowing for the formation of covalently crosslinked NP modalities. For instance, enzyme‐responsive polyethylene glycol (PEG) nanogels have been prepared via radical polymerization of acrylate monomers and an enzyme‐labile crosslinker.^[^ ^4^ ^]^ Crosslinked polymeric NPs such as nanogels are a particularly promising drug delivery platform, offering increased potential for control over drug binding and drug release properties due to their flexible and tunable synthetic nature.^[^ ^5^ ^]^

Drug‐loaded polymeric NPs are commonly formulated from degradable polymers such as poly(D,L‐lactic acid) (PLA), poly(D,L‐lactic‐*co*‐glycolic acid) (PLGA), or poly(ε‐caprolactone) (PCL) and their copolymPEG.^[^ ^6^ ^]^ Poly(α‐hydroxy acids) such as PLA and PLGA were originally developed for medical applications such as degradable sutures and remain amongst the most investigated degradable polymers.^[^ ^6^, ^7^ ^]^ The significance of these polymers can be exemplified with PLA, which has been defined as the “polymer of the 21st century” and continues to attract enormous industrial interest with an estimated global production of 190 000 tons in 2019.^[^ ^8^, ^9^ ^]^ PLGA is an FDA‐approved, biocompatible polymer that is degradable via ester hydrolysis, releasing metabolizable glycolic and lactic acids.^[^ ^10^ ^]^ PLGA enables the sustained release of drugs from long‐acting formulations allowing for reduced dosing frequency, decreased incidence of side effects, maintenance of stable plasma concentrations, and better patient compliance.^[^ ^11^ ^]^ For instance, long‐acting injectables (LAI) such as PLGA‐based microspheres enhance drug stability, bioavailability, and compliance, and are suitable for encapsulating fragile payloads such as peptides and proteins.^[^ ^12^ ^]^ Over 60 drug products based on PLGA are present on the market (2021) including microparticles and implants.^[^ ^13^ ^]^ Despite successful translation into the clinic, there are few PLGA‐based nanoscale‐formulations available on the market.^[^ ^13^ ^]^ Various translation obstacles have been suggested including difficulties in manufacturing and scale‐up, generation of acidic by‐products via degradation, poor drug loading, high initial burst release, and nanotoxicology.^[^ ^13^, ^14^ ^]^ Indeed, the lack of PLGA NPs in the clinic drives PLGA optimization as an underexplored niche within the field of nanochemistry.

Traditional modulation of PLGA properties, including hydrolytic degradation is achieved via control over polymer molecular weight and lactate/glycolate ratios.^[^ ^15^ ^]^ Seminal work by the Meyer group led to PLGA synthesis with exact control over structural‐ and stereo‐sequence via a segmer assembly polymerization (SAP) approach, which consists of the polycondensation of precisely sequenced repeating lactic and glycolic acid units (dimers, trimers, etc.), and demonstrated a strong correlation between monomer sequence, tacticity, and PLGA properties.^[^ ^16^, ^17^, ^18^, ^19^, ^20^, ^21^ ^]^ Processes such as swelling, erosion, degradation, and payload release from PLGA have all been found to be influenced by monomer sequence.^[^ ^19^ ^]^ Commonly used ring opening polymerizations of cyclic lactides and glycolides yield blocky, non‐sequenced PLGAs.^[^ ^19^ ^]^ Whilst the key components of FDA‐approved pharmaceutical products based on PLGA make use of random polymers, precisely sequenced PLGA is highly valuable for drug delivery applications.^[^ ^18^ ^]^ For example, sequenced PLGA microparticles degrade at slower rates, display a more gradual burst release, and release minimal percolating acids compared to random PLGA analogs.^[^ ^18^, ^21^ ^]^

In this work, we present a novel class of polymeric NPs inspired by sequenced PLGA chemistry and densely crosslinked polymers. We hypothesized such NPs would exhibit substantial stability in water, high drug loading efficiencies, and low rates of drug release, due to sequencing and a crosslinked structure. Specifically, we envisaged that the enhanced stability of these cross‐linked NPs, compared to conventional non‐crosslinked PLGA‐based NPs, would stem from the presence of a hydrolytically stable covalent backbone, the absence of acidic polymer end‐groups that autocatalyze degradation, and the lack of glycolate‐rich regions prone to hydrolytic breakdown. For drug delivery, these features could in turn lead to improved stability for acid‐labile payloads, reduced inflammatory responses, and slower drug release due to the suppression of degradation processes.

We prepared a small library of oligoesters named oligolactoglycolic acid dimethacrylates (OLGADMAs) as polymerizable building blocks. Here, the radical polymerization of OLGADMAs yielded what is de facto a poly(oligoester) comprised of oligoester units that are covalently bound to a carbon‐carbon backbone. OLGADMA‐based NPs are synthesized via a one‐pot nanopolymerization method we term nanoprecipitation‐polymerization, which comprises of a simultaneous radical polymerization of OLGADMAs and formation of NPs. The methodology is surfactant‐free and allows for the in situ encapsulation of dexamethasone, a model hydrophobic drug. The physicochemical analysis of the obtained NPs was carried out in comparison to NPs obtained from commercially available PLGA polymers. The stability of the OLGADMA‐based NPs at physiological temperature over 5 weeks was found to be superior to that of PLGA‐based NPs, which may be attributed to the slower generation of acidic pH. The pharmaceutical potential of long‐lasting OLGADMA‐based NPs is supported by high drug loading efficiencies, good stability in a protein‐rich biological medium, stability during storage at room temperature, and an excellent cytotoxicity profile in vitro.

## 2 Results and Discussion

### 2.1 Design of OLGADMAs

**Figure 1:**
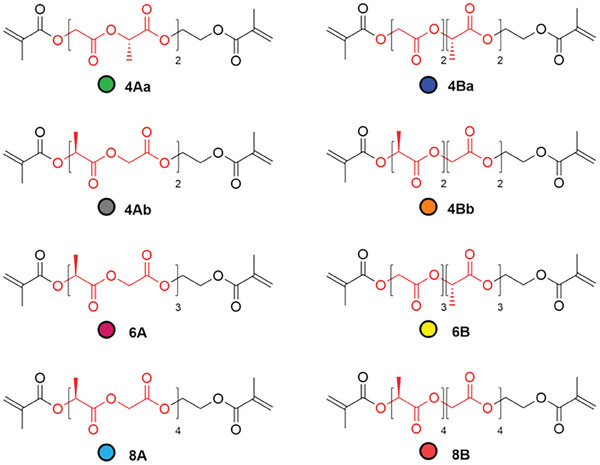
Chemical structure of precisely sequenced OLGADMAs prepared in this work along with color‐code. Red sections on structures denote lactate and glycolate sequences.

Given the structure and composition of OLGADMAs as fundamental building blocks used in this work, it was postulated that the NPs resulting from their polymerization would exhibit: i) elevated physical and chemical stability due to covalent crosslinking and ester sequencing, ii) potential for drug encapsulation and drug release tunability due to the design flexibility related to oligoester sequence and length, and iii) intrinsic synthetic flexibility of the radical polymerization platform which may enable the diversification of the NP composition via copolymerization with additional monomers in a single synthetic step. Furthermore, to mitigate the poor solubility of OLGADMAs, we developed a two‐solvent nanoprecipitation polymerization methodology. First, we synthesized eight precisely sequenced OLGADMAs which differ in the type of sequence and number of lactate and glycolate units (**Figure** **1**). The compounds were prepared using non‐racemic *L*‐lactate derivatives and maintaining the ratio of lactate versus glycolate units at 1. To this end, we prepared OLGADMAs with alternating (**A)** and block (**B)** sequences comprising of tetramers (**4)**, hexamers (**6),** and octamers (**8)**. Tetramers have been prepared in both directions of the sequence relative to the end groups and are further designated with **a** or **b**. The NPs prepared in this work are designated with **NP** followed by the type of OLGADMA used for the synthesis or PLGA (e.g., **NP4Aa** denotes NPs prepared using OLGADMA **4Aa**). Analogously, dexamethasone‐loaded NPs prepared in this work are designated with **DNP** followed by the type of OLGADMA used for the preparation or PLGA (e.g., **DNP4Aa** denotes dexamethasone‐loaded NPs prepared using OLGADMA **4Aa**).

### 2.2 Synthesis of Precisely Sequenced OLGADMAs

**Scheme 1:**
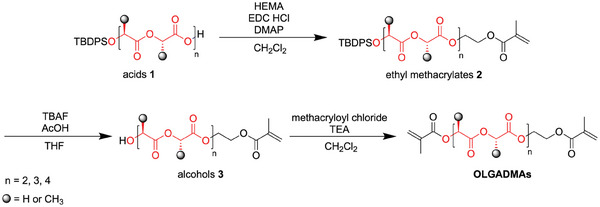
General synthesis of precisely sequenced OLGADMAs from acids **1**. The section of the structure highlighted in red is a general representation of both alternating and block oligolactoglycolic sequences.

Precisely sequenced OLGADMAs were synthesized from sequenced acids **1** via a three‐step synthetic procedure (**Scheme** **1**). Acids **1** required for this work were prepared by a multi‐step synthesis, using previously reported procedures with modifications (S2, Supporting Information).^[^ ^16^, ^22^ ^]^ Functionalization of acids **1** with HEMA was achieved via carbodiimide chemistry to afford ethyl methacrylates **2** in 58–89% yields. Cleavage of the silyl ether moiety of **2** with buffered TBAF gave alcohols **3** in 60–86% yields. The desired OLGADMAs were prepared by methacrylation of alcohols **3** with methacryloyl chloride in the presence of TEA. OLGADMAs were obtained as water‐insoluble oils at room temperature. The characterization of all prepared compounds is presented in S16 (Supporting Information).

### 2.3 Synthesis of OLGADMA‐Based Nanoparticles

**Scheme 2:**
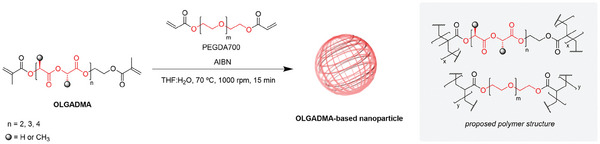
Synthesis of OLGADMA‐based NPs via nanoprecipitation polymerization of OLGADMAs.

The synthesis of polymeric NPs was achieved via nanoprecipitation polymerization of OLGADMAs initiated by AIBN (**Scheme** **2**). Given the poor aqueous solubility of OLGADMAs, it was hypothesized that these compounds could be successfully dispersed in water via a procedure analogous to the nanoprecipitation methodology. The resulting dispersion of oil droplets in water could then undergo radical polymerization in the presence of a radical initiator, affording an aqueous suspension of polymeric NPs. In this context, a procedure was sought that could enable the activation of the radical initiator and removal of the water‐miscible organic solvent from the reaction system. Ultimately, the rapid injection of a THF solution of OLGADMAs, or their mixtures with PEGylated monomers, and AIBN into vigorously stirred water at 70 °C and under an inert atmosphere proved successful in providing polymeric NPs. The novel nanopolymerization methodology was termed nanoprecipitation polymerization (S7, Supporting Information).

Whilst it was possible to synthesize OLGADMA‐based NPs using OLGADMAs as the only monomer, the presence of the PEGDA700 monomer in the reaction mixture proved beneficial for drug encapsulation. Particularly, it was observed that a higher concentration of dexamethasone could be tolerated by the reaction system without compromising the NP stability and yield when PEGDA700 was present (S18, Supporting Information). Therefore, the standard monomer combination employed throughout this work comprises OLGADMAs and PEGDA700 in a 1:1 ratio by weight.

Purifications of the crude reaction mixtures were achieved via repetitive centrifugal filtration. Dilution with deionized water afforded the desired NP suspensions. The reaction of six OLGADMAs successfully yielded NP suspensions, without requiring further sample manipulation such as sonication or filtration. Compounds **4Ba** and **8A** did not yield suitable NPs. While polymerization of OLGADMAs did occur in these reactions, the material obtained was consistently a macroscopic bulk solid which would form via a rapid precipitation during the reaction and could not be re‐suspended. While the origin of this behavior remains elusive, a possible explanation for the observed divergencies could comprise the existence of markedly different preferred conformations in solution for OLGADMA **4Bb** and **8A** which disfavor the formation of stable NPs over macroscopic solids.

**Figure 2:**
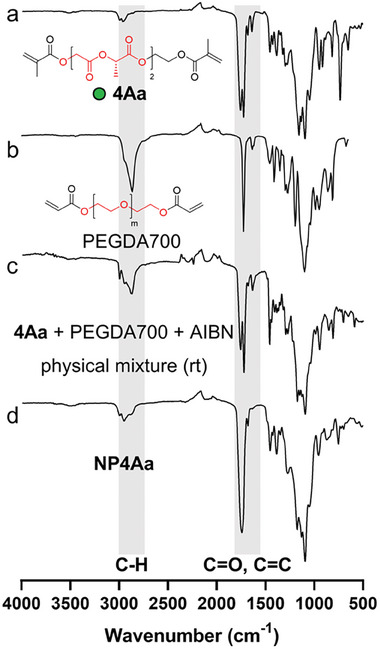
ATR FTIR spectra of a) **4Aa**, b) PEGDA700, c) physical mixture of **4Aa**, PEGDA700, and AIBN at reaction ratios and room temperature, and d) **NP4Aa**.

The successful polymerization of OLGADMAs was confirmed via FTIR analysis of lyophilized NPs (S8, Supporting Information). The conversion of methacrylates can be evidenced by the absence of the vinyl C═C stretch ≈1635 cm^−1^ in the spectra of NPs, as seen in a typical FTIR spectrum of **NP4Aa** (**Figure** **2**). Furthermore, the conjugated carbonyl stretch visible ≈1720 cm^−1^ in the FTIR spectra of the starting materials is absent in the spectrum of the NPs. The two distinct vibrations in the carbonyl stretch region belonging to α‐hydroxy acid esters (≈1750 cm^−1^) and those deriving from (meth)acrylic esters respectively (≈1720 cm^−1^), are superimposed in a single broad vibration in the FTIR spectrum of the NPs. A prominent stretch vibration of the nitrile group deriving from the initiator AIBN which is expected ≈2240 cm^−1^ could not be detected by FTIR in any of the NPs prepared. The same FTIR trends were observed for all OLGADMAs that were successfully polymerized into NPs.

**Figure 3:**
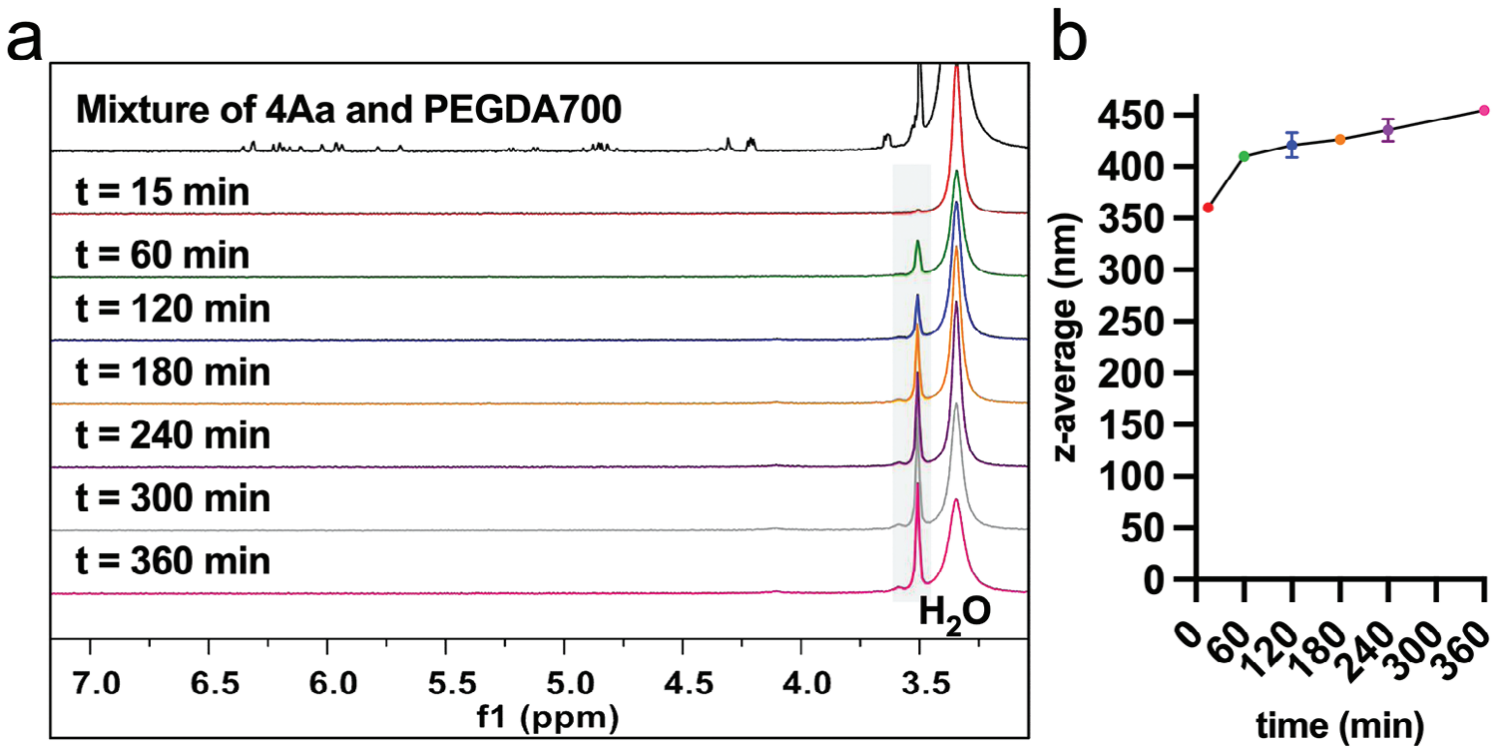
a) Comparison of ^1^H NMR (400 MHz, (CD_3_)_2_SO) spectra of a mixture of **4Aa** and PEGDA700 at standard reaction concentrations, and the crude reaction mixture of the nanoprecipitation polymerization of **4Aa** and PEGDA700 recorded over 6 h (PEG resonance at 3.51 ppm highlighted in grey) and b) evolution of nanoparticle size expressed as z‐average during nanoprecipitation polymerization of **4Aa** and PEGDA 700, measured by DLS analysis of the crude reaction mixture over 6 h.

The nanoprecipitation polymerization reaction was monitored over 6 h via ^1^H NMR spectroscopy and dynamic light scattering (DLS) (S20, Supporting Information), using a mixture of compound **4Aa** and PEGDA700 under standard reaction conditions (**Figure** **3**). The reaction appears to be a fast process, with no starting materials detectable by ^1^H NMR after 15 min of reaction. The polymeric NP itself was not detected by ^1^H NMR, possibly due to factors such as concentration, poor solvation, and densely crosslinked structure. At reaction times longer than 15 min, the presence of a hypothetical PEG species becomes NMR‐visible as a resonance of increasing intensity at 3.51 ppm. The simultaneous DLS analysis of the crude reaction mixture denotes a gradual and time‐dependent increase in NP size. These observations could be indicative of the existence of an initial NP formation step followed by swelling of the NPs caused by hydrolytic degradation in water at 70 °C, and thus we limited the reaction time to 15 min throughout the study.

### 2.4 Synthesis of Dexamethasone‐Loaded OLGADMA‐Based Nanoparticles

**Figure 4:**
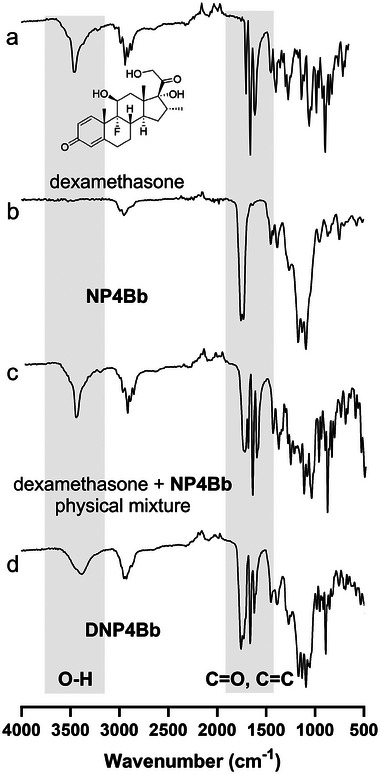
ATR FTIR spectra of a) dexamethasone, b) **NP4Bb**, c) physical mixture of dexamethasone and **NP4Bb** in a 1:1 weight ratio, and d) **DNP4Bb**. The O─H, C═O, and C═C vibrations are highlighted in grey.

To test the potential of OLGADMA‐based NPs as drug nanocarriers we encapsulated dexamethasone, a potent synthetic glucocorticoid.^[^ ^23^ ^]^ Dexamethasone is poorly soluble in water (89 µg mL^−1^ at 25 °C) and its administration via injection is achieved using the inactive phosphate form as a prodrug.^[^ ^24^ ^]^ We envisaged the encapsulation of dexamethasone within OLGADMA‐based NPs would facilitate a pseudo‐solubilization of substantial quantities of the drug, which could then be slowly released from the NPs. Dexamethasone was encapsulated in situ, via solubilization into the THF solution used for nanoprecipitation‐polymerization. The chemical stability of dexamethasone during the nanoprecipitation polymerization was confirmed as the drug could be recovered qualitatively and quantitatively after being subjected to typical reaction conditions (S21, Supporting Information). Furthermore, the ^1^H NMR analysis of the filtrates obtained during drug release assays confirmed the release of structurally intact dexamethasone (S22, Supporting Information). NP suspensions containing dexamethasone were obtained from six out of eight OLGADMAs. Analogously to what was observed with the empty NPs, the reactions of compounds **4Ba** and **8A** failed to yield drug‐loaded NP suspensions. The presence of dexamethasone in NP suspensions was confirmed by FTIR analysis where vibrations characteristic of the drug are observable (**Figure** **4**). A comparison of **DNP4Bb** and a physical mixture obtained by grinding of **NP4Bb** and dexamethasone solid reveals differences between the FTIR spectra. This is suggestive of alternative or stronger drug‐polymer interactions in the drug‐containing **DNP4Bb** compared to a mere physical mixture of empty nanoparticles **N4Bb** and the drug.

The ability to tune the size of drug‐loaded NPs is highly desirable for drug delivery applications. Importantly, we found how dexamethasone‐loaded OLGADMA‐based NPs with sizes ranging from 105 to 313 nm could be synthesized from **4Aa** by simple variation of the duration of the nitrogen purging step of nanoprecipitation polymerization (S23, Supporting Information). Particularly, we found longer purging of the reaction mixture induced the formation of smaller dexamethasone‐loaded OLGADMA‐based NPs. This observation could be explained via a local reduction in the temperature of the reaction mixture deriving from the contact with room temperature nitrogen gas and an increased area available for THF evaporation which in combination could evoke a slower decomposition of the radical initiator (AIBN). The NPs presented in this work were prepared according to standard conditions comprising a 5 min purging time which enabled the highest drug encapsulation efficiencies (EE) among the tested purging durations.

### 2.5 Synthesis of PLGA‐Based Nanoparticles

PLGA‐based control NPs were prepared from three types of PLGA‐based polymers. For this purpose, **PLGA‐PEG** (block polymer, acid terminated, L:G 50:50, *M*_n_ 50 000:5000 Da PLGA:PEG) and **PLGA** (acid terminated, L:G 50:50, *M*_w_ 38000‐54 000 Da) were obtained from commercial sources. Alternating PLGA (**PLGA alt**) (sequenced, acid‐terminated, L:G 50:50, *M*_w_ 7158 Da) was synthesized according to procedures reported by Stayshich and Meyer (S24, Supporting Information).^[^ ^22^ ^]^ Empty and dexamethasone‐loaded PLGA‐based NPs were formulated using conditions that mirror the nanoprecipitation polymerization reaction. PLGA‐based NPs were subjected to purification and analysis conditions identical to those of OLGADMA‐based NPs. The empty PLGA‐based NPs prepared from **PLGA**, **PLGA alt,** and **PLGA‐PEG** are designated with **NP‐PLGA**, **NP‐PLGA alt**, and **NP‐PLGA‐PEG**, respectively. Conversely, the dexamethasone‐loaded NPs prepared from PLGA‐based polymers are designated with **DNP‐PLGA**, **DNP‐PLGA alt**, and **DNP‐PLGA‐PEG**.

### 2.6 Characterization and Comparison of OLGADMA‐Based and PLGA Nanoparticles

**Figure 5:**
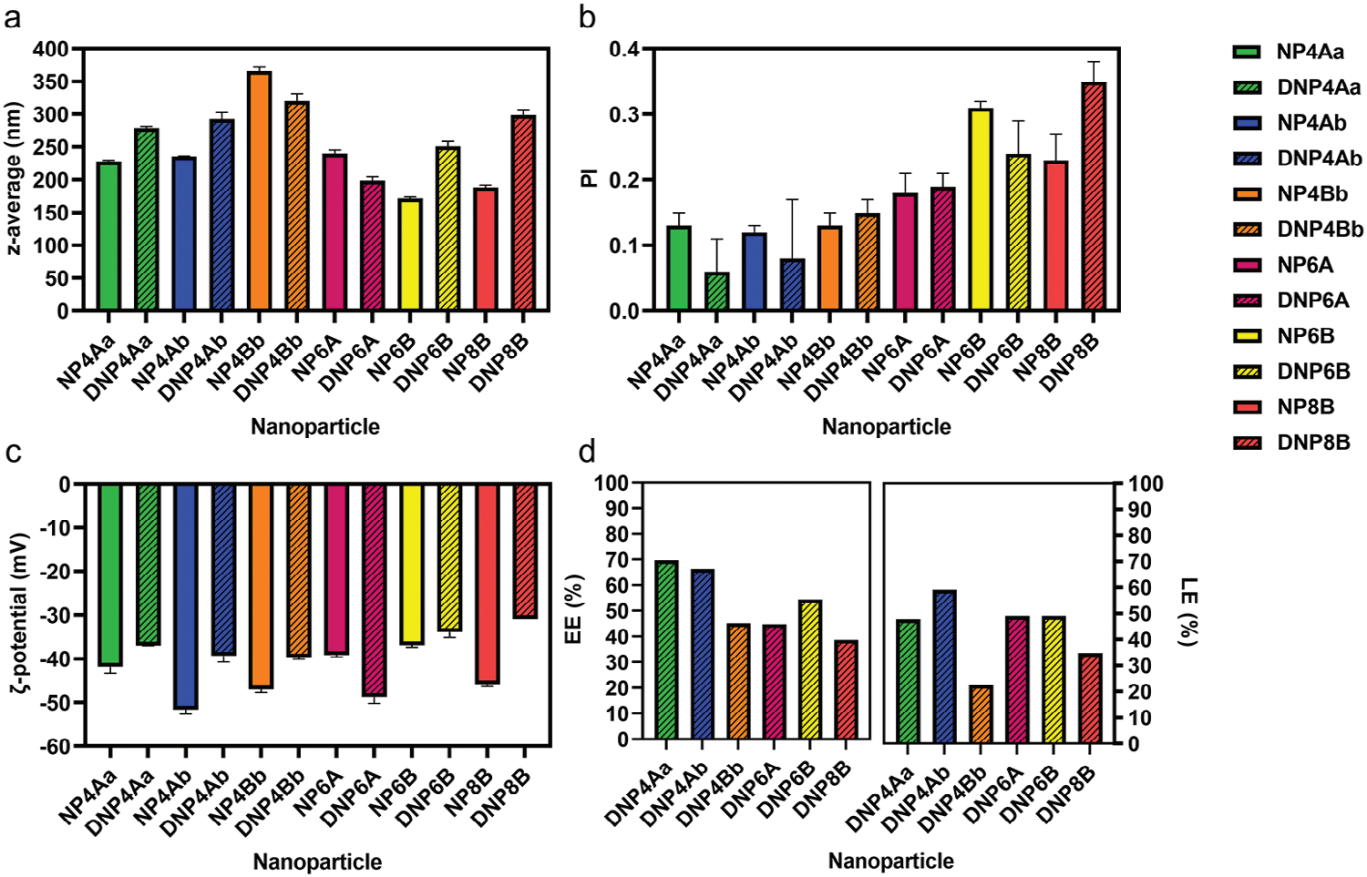
a) size expressed as z‐average, b) polydispersity index (PI), c) ζ‐potential, and d) estimation of encapsulation efficiency (EE) and loading efficiency (LE) of OLGADMA‐based NPs.

Initially, the physicochemical properties of both empty and drug‐loaded OLGADMA‐derived NPs were measured (**Figure** **5**). The size of OLGADMA‐based NPs, measured by DLS and expressed as z‐average, ranged from 172 to 366 nm. Dexamethasone‐loaded OLGADMA‐based NPs had sizes ranging from 199 to 321 nm. Intensity‐weighted size distributions and corresponding correlation functions of all NPs prepared in this work are presented in S26 (Supporting Information). The polydispersity (PI) parameter of the prepared OLGADMA‐based NPs was below 0.4, with NPs obtained from tetramer OLGADMs having an especially low PI (<0.2) indicating a homogeneous size distribution. OLGADMA‐based NPs exhibited negative ζ‐potential in deionized water at 25 °C, as measured by electrophoretic light scattering (ELS). The ζ‐potential of dexamethasone‐loaded OLGADMA‐based NPs was generally less negative than OLGADM NPs, possibly due to differences in surface chemistries deriving from partial surface adsorption of dexamethasone and/or NP size. The drug encapsulation efficiency (EE) of dexamethasone‐loaded NPs was calculated via quantitation of the non‐encapsulated dexamethasone (S27, Supporting Information). OLGADMA‐based NPs had EEs in the range of 39–70% (Figure 5). The highest EE was achieved with **DNP4Aa,** while **DNP8B** had the lowest EE. The drug loading efficiency (LE) was estimated from the EE and the mass of solids obtained after 48 h of freeze‐drying of dexamethasone‐loaded NP suspensions. Dexamethasone‐loaded OLGADMA‐based NPs had LEs ranging from 23–59%.

**Figure 6:**
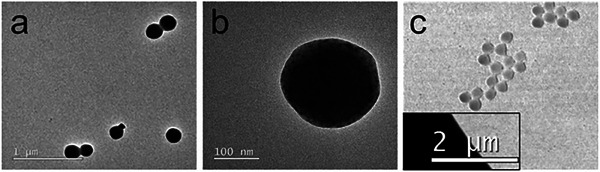
TEM micrographs of a) **NP4Aa** (80 kV, 8000x, scale bar = 1 µm), b) **NP4Aa** (80 kV, 50000x, scale bar = 100 nm), and c) **DNP4Aa** (80 kV, 2000x, scale bar = 2 µm).

Samples of **NP4Aa** and **DNP4Aa** were further analyzed by transmission electron microscopy (TEM) (S28, Supporting Information) which confirmed the presence of spherical NPs (**Figure** **6**). TEM micrographs of **NP4Aa** show spherical NPs presenting a dotted pattern accompanied by a matter‐dense core and a less‐dense periphery.

**Figure 7:**
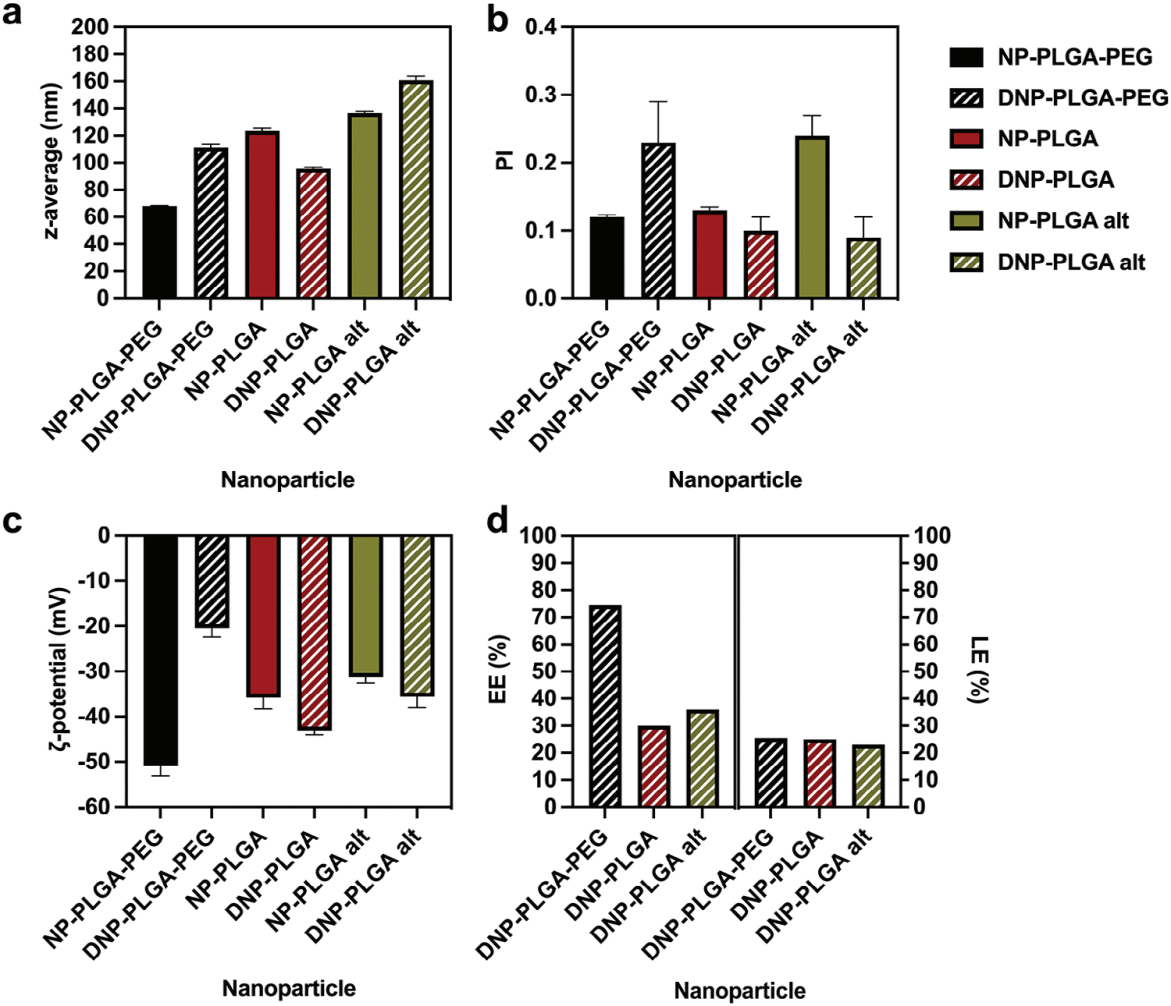
a) size expressed as z‐average, b) polydispersity index (PI), c) ζ‐potential, and d) estimation of encapsulation efficiency (EE) and loading efficiency (LE) of PLGA‐based NPs.

Subsequently, the empty and drug‐loaded control PLGA‐based NPs were analyzed in the same manner (**Figure** **7**). Compared to OLGADMA‐based NPs, PLGA‐based NPs exhibited a smaller size overall, whilst the ζ‐potential was comparatively negative in deionized water. Specifically, the sizes of the prepared PLGA‐based NPs were all <180 nm with PI values <0.3, as determined by DLS. The ζ‐potential of PLGA‐based NPs was negative in deionized water, having values below −15 mV. **NP‐PLGA‐PEG** presented the smallest size, lowest PI, and the most negative ζ‐potential among the three empty PLGA‐based examples. The EE of **DNP‐PLGA** and **DNP‐PLGA alt** was lower than that of OLGADMA‐based NPs, whilst a higher EE was achieved with **DNP‐PLGA‐PEG**. The estimated LE of PLGA‐based NPs was generally lower than that of OLGA‐based NPs, except for **DNP4Bb** (Figure 7).

### 2.7 Comparative Stability Study of OLGADMA‐Based and PLGA‐Based Nanoparticles at 37 °C in Water

PLGA microparticles develop an acidic core and undergo swelling during incubation at or above the physiological temperature in an aqueous medium, due to hydrolytic polymer degradation.^[^ ^25^, ^26^ ^]^ While these processes can affect carrier size, cargo stability, and drug release kinetics, they are often poorly understood at the nanoscale. OLGADMA‐based NPs were expected to exhibit elevated stability in aqueous environments due to the crosslinked structure and the presence of sequenced oligoesters. To test this hypothesis, the stability of the prepared empty OLGA‐based NPs was assessed via incubation of aqueous NP suspensions at the physiological temperature of 37 °C followed by the determination of the NP size, PI, ζ‐potential, and pH of the suspensions over a period of 5 weeks. The results were compared against the identical analysis of empty PLGA‐based NPs prepared from three types of PLGA polymers presented above. Specifically, these included a non‐sequenced form of PLGA, a sequenced form of PLGA, and a PEGylated form of PLGA which share chemical composition similarities with OLGA‐based NPs and were hypothesized to yield NPs that cover a range of different stabilities under experimental conditions.

**Figure 8:**
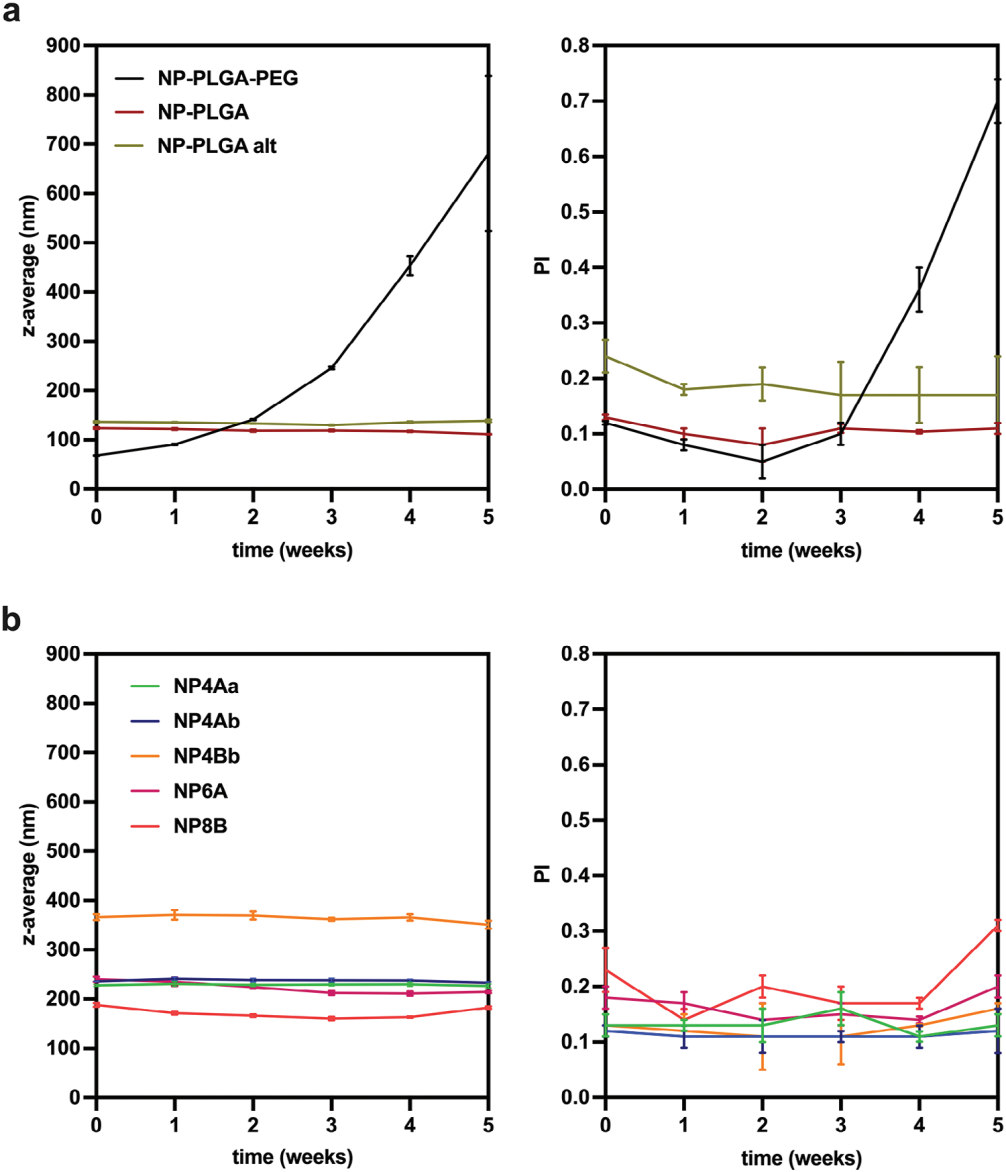
Analysis of nanoparticle size expressed as z‐average and polydispersity index (PI) during 5 weeks of incubation at 37 °C in deionized water. a) PLGA‐based NPs and b) OLGADMA‐based NPs.

The DLS analysis of PLGA‐based NPs demonstrated that **NP‐PLGA** and **NP‐PLGA alt** exhibit elevated size stability under experimental conditions outlined above (**Figure** **8**a). This finding was substantiated by consistently stable z‐average and PI values detected throughout the study. Conversely, **NP‐PLGA‐PEG** manifested a progressive increase in size, ultimately increasing 10‐fold by the end of the study. Particularly, the size of **NP‐PLGA‐PEG** doubled after two weeks under experimental conditions after which, a faster increase in size and PI, suggestive of NP aggregation, was detected. In comparison, five out of six OLGA‐based NPs showed good size stability, with only limited size variations detected throughout the study (Figure 8b). Specifically, **NP4Aa** and **NP4Ab** showcased size variations within a narrow window of <10 nm, whilst the size variations of **NP4Bb**, **NP6A**, and **NP8B** did not exceed 30 nm. Moreover, the PI values remained ≤0.2, with only a slight increase above this value detected for **NP8B** after five weeks at 37 °C. Exceptionally, **NP6B** displayed sudden colloidal instability which manifested via the formation of a precipitate within the first week of the study and was therefore discarded from further assessments. Taken altogether, five types of OLGA‐based NPs displayed size stability similar to that of NPs prepared from **PLGA** and **PLGA alt**, regardless of the similarities in chemical composition to the markedly less size‐stable **NP‐PLGA‐PEG**.

**Figure 9:**
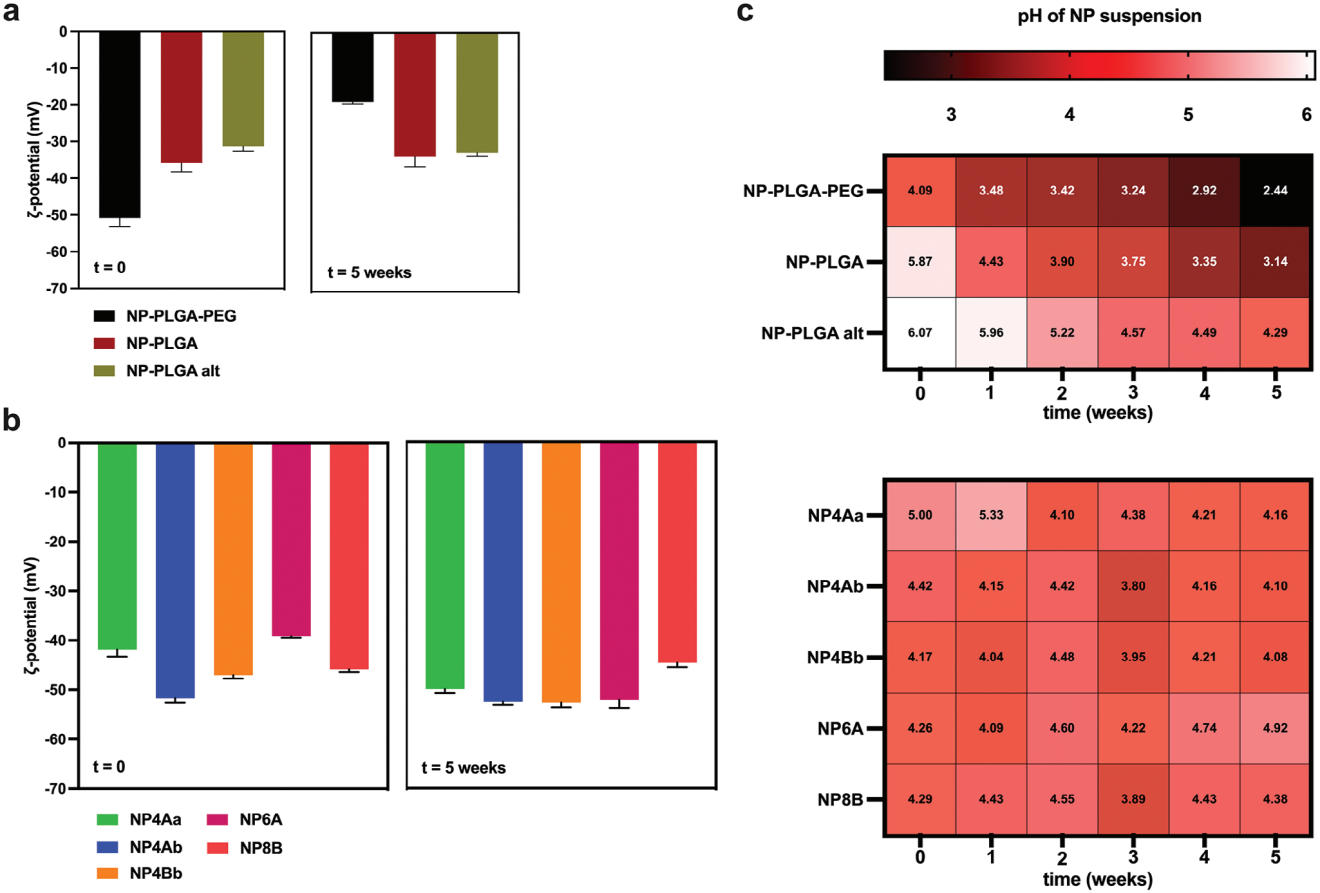
Analysis of ζ‐potential and NP suspension pH during 5 weeks of incubation at 37 °C. a) initial and final ζ‐potential of PLGA‐based NPs, b) initial and final ζ‐potential of OLGADMA‐based NPs, and c) pH of aqueous NP suspensions containing PLGA‐based and OLGADMA‐based NPs.

The examination of the ζ‐potential of PLGA‐based via ELS showed variability with **NP‐PLGA** and **NP‐PLGA alt** with values remaining within the −31.29 ± 1.28 and −42.15 ± 1.31 mV window (initial and final ζ‐potential values are presented in **Figure** **9**a). On the other hand, **NP‐PLGA‐PEG** exhibited a clear trend toward progressively less negative ζ‐potential values which evolved from −50.84 ± 2.30 to −18.75 ± 0.54 mV. The ζ‐potential of OLGADMA‐based NPs showed similar behavior to that of **NP‐PLGA** and **NP‐PLGA alt**, with variable values remaining within the −27.55 ± 3.80 and −55.63 ± 1.93 mV window (initial and final ζ‐potential values are presented in Figure 9b). Moreover, a clear trend toward less negative ζ‐potential values, such as observed with **NP‐PLGA‐PEG**, was not evident with OLGA‐based NPs.

The simultaneous analysis of the pH of the NP suspensions is presented in Figure 9c. The pH analysis revealed the freshly prepared non‐buffered suspensions of PLGA‐based NPs displayed an acidic pH ranging from 4.09 for **NP‐PLGA‐PEG** to 6.07 for **NP‐PLGA alt**. A gradual progression toward more acidic values was detected with PLGA‐based NPs over the course of the study. The suspension subjected to 5 weeks of experimental conditions containing **NP‐PLGA‐PEG** was the most acidic (pH 2.44), followed by **NP‐PLGA** (pH 3.14), and **NP‐PLGA alt** (pH 4.29). These results may be reflective of the hydrolytic stability of the different PLGA forms under experimental conditions and are congruent with previously reported properties of MPs and NPs prepared from these polymers. Analogously to PLGA‐based NPs, the pH of freshly prepared OLGADMA‐based NPs was acidic ranging from pH 4.17 to pH 5.00. However, contrary to PLGA‐based NPs the trend of progressive acidification of the suspension containing OLGADMA‐based NPs was not observed. Particularly, the pH of the examined NP suspensions demonstrated a slight variability throughout the study with the pH values at all time points remaining within a relatively narrow window of ≈1.5 pH units. These findings could potentially be explained by the increased hydrolytic stability of the NPs deriving from the presence of sequenced oligoesters and/or the absence of free carboxylic acids in the NP building blocks, which are known to catalyze the hydrolysis of ester‐containing polymers.

Taken together, OLGADMA‐based NPs maintained a good level of size stability in an aqueous environment at the physiological temperature of 37 °C for at least five weeks. Moreover, aqueous suspensions of OLGADMA‐based NPs retain the pH within a narrow window over 5 weeks at 37 °C, whilst PLGA‐based NPs showcase gradual acidification. Despite being PEGylated, the size‐stability of OLGADMA‐based NPs at 37 °C was found to be similar to that of NPs prepared from PLGA and sequenced PLGA, whilst being markedly more stable than NPs prepared from PLGA‐PEG. Additionally, the expected higher hydrolytic susceptibility of OLGADMA‐based NPs containing block oligolactoglycolic sequences deriving from the presence of the more labile glycolic‐glycolic linkages could not be detected in the studied examples, possibly due to a strong stabilizing effect of covalent crosslinking and the absence of acidic end‐groups in the utilized monomers.

**Figure 10:**
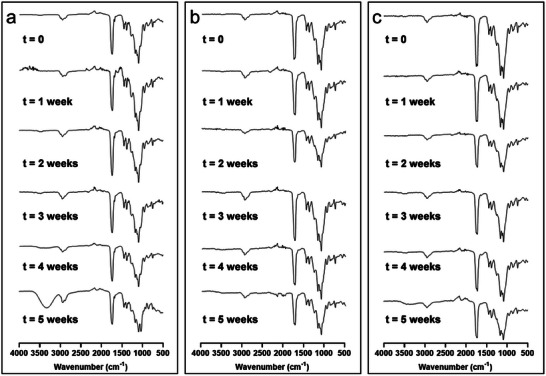
ATR FTIR spectra of a) **NP4Aa**, b) **NP4Ab,** and c) **NP4Bb** over 5 weeks in deionized water at 37 °C.

NPs made from tetramer OLGADMs were further analyzed by FTIR spectroscopy, to identify and compare potential changes in NP chemical composition. While the FTIR spectrum of **NP4Ab** remains identical over the course of the experiment, spectral changes have been detected with **NP4Aa** and **NP4Bb** (**Figure** **10**). An evolution of a broad vibration in the OH stretch region becomes visible in both spectra and is more pronounced with **NP4Aa**. Furthermore, changes in the fingerprint region are detectable in **NP4Aa**, which are particularly visible at week 5. We hypothesize these changes to derive from partial hydrolysis of esters under experimental conditions. We emphasize the observed changes in the chemical composition were not accompanied by a simultaneous variation of the size of the NPs, as confirmed by DLS.

### 2.8 Dexamethasone Release at 37 °C

**Figure 11:**
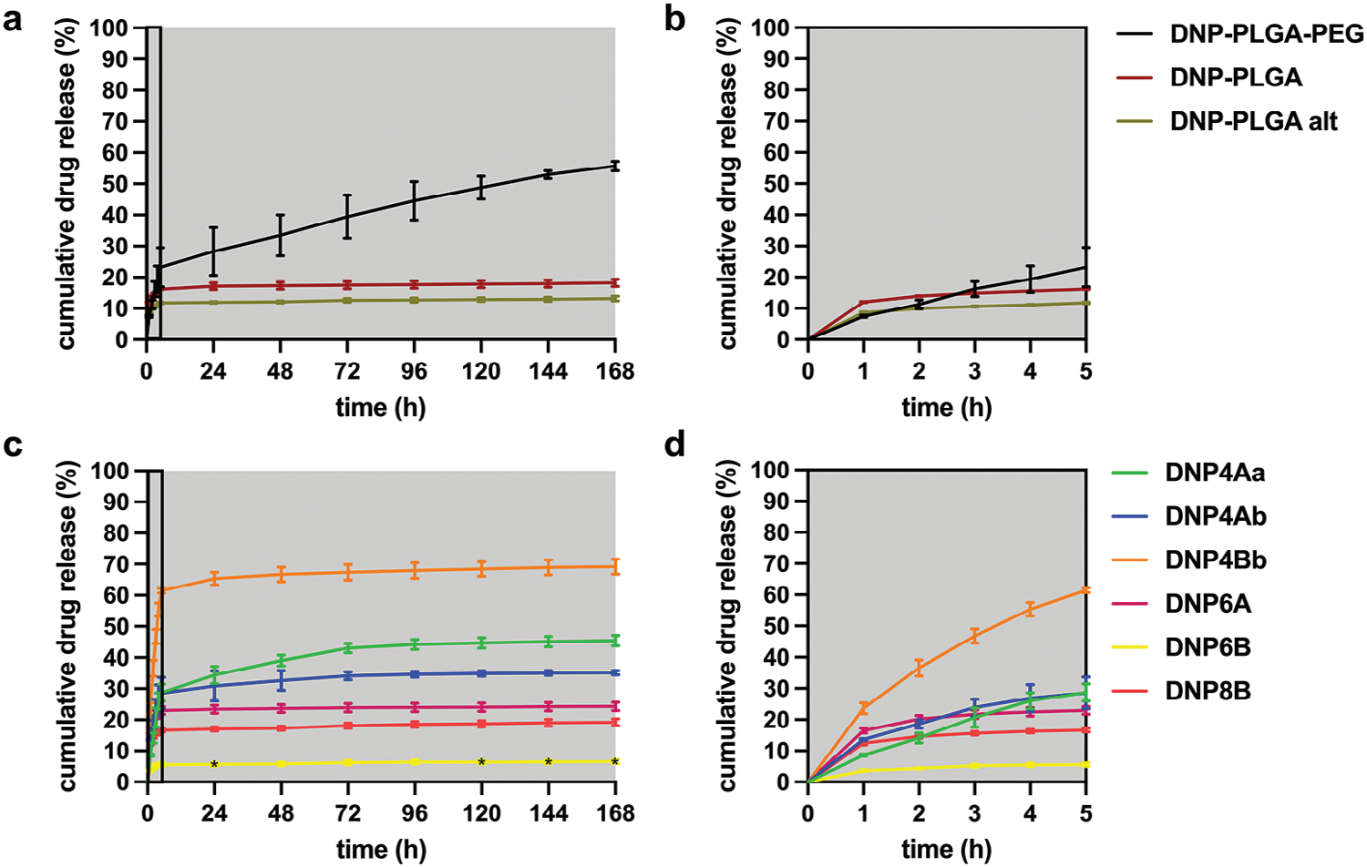
Cumulative release of dexamethasone from dexamethasone‐loaded NPs under experimental conditions: a) PLGA‐based NPs (phase 1 and phase 2 combined), b) PLGA‐based NPs (phase 1 enlarged), c) OLGADMA‐based NPs (phase 1 and phase 2 combined), and d) OLGADMA‐based NPs (phase 1 enlarged). Concentrations of released dexamethasone below the limit of quantitation are labeled with an asterisk.

The library of dexamethasone‐loaded NPs was screened in terms of their ability to release the encapsulated dexamethasone when subjected to a predetermined set of experimental conditions. The aim of this study was to i) compare the drug release of OLGADMA‐based NPs against PLGA‐based NPs, as an established class of NPs for drug delivery applications, ii) identify potential differences in drug release among the prepared types of OLGADMA‐based NPs. Dexamethasone‐loaded NPs were subjected to an adaptation of the “sample and separate” method which consisted of the incubation of aqueous NP suspensions within centrifugal filters at 37 °C, followed by centrifugation, re‐suspension, and quantitation of the dexamethasone content of the filtrates at different time points. This procedure was performed hourly over the first five hours of the study (phase 1), followed by daily intervals (phase 2). The experiment was performed over the course of a 7‐day period. As expected, the release of dexamethasone from PLGA‐based NPs under experimental conditions revealed substantial differences between the PEGylated and the non‐PEGylated NPs (**Figure** **11**a,b). Particularly, **DNP‐PLGA‐PEG** released dexamethasone at a higher rate than **DNP‐PLGA** and **DNP‐PLGA alt** throughout the study. Notably, **DNP‐PLGA‐PEG** NPs were able to release 3% to 23% of the encapsulated dexamethasone daily, whilst **DNP‐PLGA** and **DNP‐PLGA alt** released 0.1% to 16% and 0.1% to 12%, respectively. Importantly, the cumulative release of dexamethasone over 7 days under experimental conditions was the highest for **DNP‐PLGA‐PEG** (56%), followed by **DNP‐PLGA** (18%), and **DNP‐PLGA alt** (13%).

The release of dexamethasone from OLGADMA‐based NPs was followed via an identical procedure (Figure 11c,d). Interestingly, the different types of OLGADMA‐based NPs exhibited a range of cumulative releases of dexamethasone ranging from 7% to 69%, measured after 7 days under the experimental conditions outlined above. **DNP4Bb** released the highest amount of encapsulated dexamethasone, whilst **DNP6B** released the smallest amount. Particularly, samples containing **DNP6B** were releasing between 0.1% and 6% of their dexamethasone content daily, and as such were the slowest‐releasing NPs of the entire library of dexamethasone‐loaded NPs investigated in this work. The differential extent of dexamethasone release from OLGADMA‐based NPs could be reflective of different degrees of porosity present within these NPs, possibly deriving from different crosslinked network structures dependent of the length and conformational flexibility of the different OLGADMA monomers. Regarding the drug release mechanism, we hypothesize that diffusion processes dominate during the investigated period, as evidenced by the stable particle size and minimal pH fluctuations of OLGADMA‐based NP suspensions, rather than polymer degradation (erosion) or swelling.

### 2.9 Stability of Dexamethasone‐Loaded NPs in Biological Media

**Figure 12:**
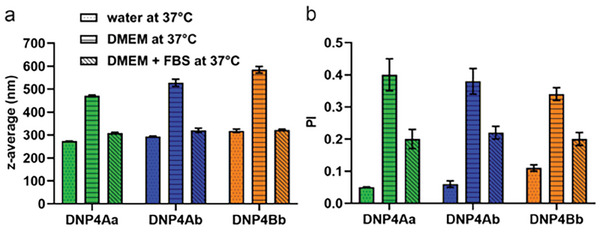
a) NP size expressed as z‐average and b) PI of **DNP4Aa**, **DNP4Ab,** and **DNP4Bb** in water at room temperature and after 2 h of incubation in water, DMEM, and DMEM+FBS at 37 °C.

Many of the synthetic chemistry developed in the past yields NPs which are not stable in biological fluids.^[^ ^27^ ^]^ Contrary to dispersants such as deionized water, complete cell culture medium is rich in electrolytes and proteins. These two components can alter the colloidal stability of NPs resulting in modifications of their behavior in vitro and in vivo. For instance, aggregation can influence the dosimetry, cellular uptake, and toxicity of NPs in vitro, and affect pharmacokinetics, biodistribution, and toxicity in vivo.^[^ ^28^ ^]^ To assess the colloidal stability in the presence of salts and proteins, we compared the size of the top three ideal synthesized NPs; **DNP4Aa**, **DNP4Ab,** and **DNP4Bb** in (i) deionized water, (ii) DMEM, and (iii) FBS‐supplemented DMEM after incubation at 37 °C (**Figure** **12**). The NPs retained their initial size after 2 h of incubation in deionized water, while the PI values slightly decreased. Incubation in DMEM led to an 80% increase in size on average. The simultaneous increase in PI values is strongly suggestive of particle aggregation. Conversely, incubation in DMEM supplemented with FBS (10%) induced only a minimal increase in size. The detected size increments were roughly in the range of 4–35 nm, while the PI values were maintained below the 0.3 value. We hypothesize the tested NPs naturally rely on electrostatic stabilization, which becomes challenged in an electrolyte‐rich medium such as DMEM. This detrimental effect is lessened in the presence of proteins, possibly due to protein adsorption leading to the formation of a stabilizing protein corona.

### 2.10 Stability of Dexamethasone‐Loaded NPs During Storage at Room Temperature

**Figure 13:**
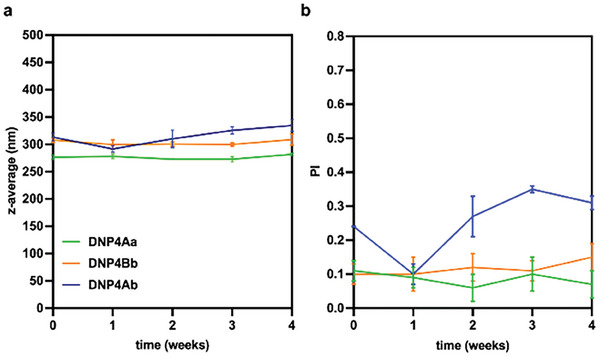
a) size expressed as z‐average and b) polydispersity index (PI) of **DNP4Aa**, **DNP4Ab,** and **DNP4Bb** over 4 weeks of storage at room temperature, in the absence of mechanical stirring.

The investigation of NP stability under storage conditions is one of the essential aspects of developing nanomedicines.^[^ ^29^ ^]^ We aimed at establishing the colloidal stability of **DNP4Aa**, **DNP4Ab,** and **DNP4Bb** during storage at room temperature (16–25 °C), exposed to moderate levels of natural light, and in the absence of mechanical stirring. For this purpose, NP suspensions were placed in transparent vials and stored on the bench over a period of 4 weeks. The suspensions were gently mixed by three inversions of the vial and analyzed by DLS weekly (**Figure** **13**). **DNP4Aa** and **DNP4Bb** maintained excellent size stability over time, while **DNP4Ab** showed only marginal size variations. The PI of the suspensions was maintained below 0.4 and was most variable for **DNP4Ab**. The ζ‐potential was most stable for **DNP4Bb**, while **DNP4Aa** and **DNP4Ab** showed a slight evolution toward less negative values (S29, Supporting Information).

### 2.11 Cytotoxicity and Cellular Uptake In Vitro

**Figure 14:**
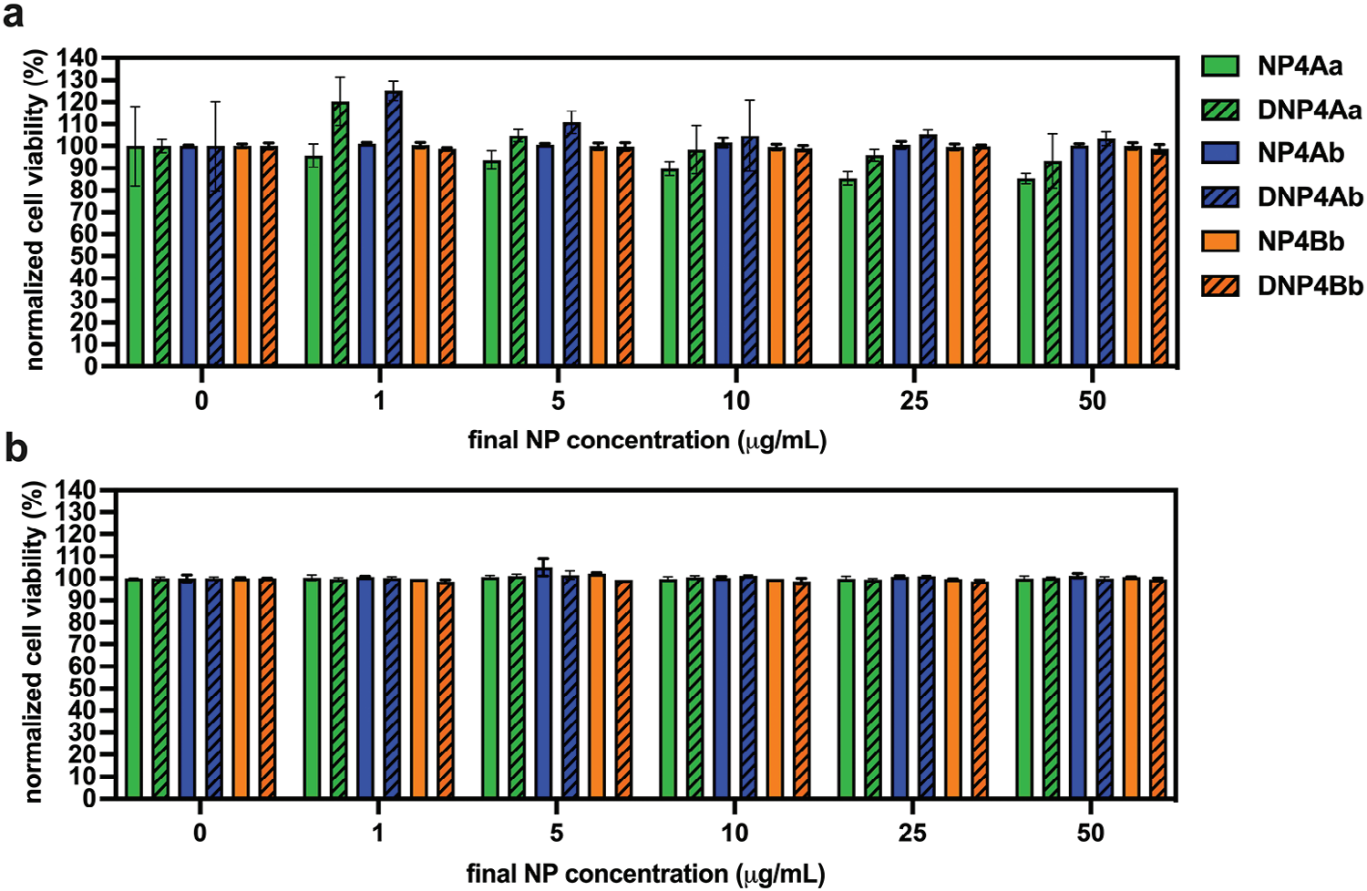
Viability of HeLa cells upon a) 24 h of incubation and b) 48 h of incubation with NPs (1–50 µg mL^−1^) prepared from tetramer OLGADMAs, measured by CCK‐8 assay. The viabilities are normalized to control cells treated with the dispersant alone (water).

To assess the cytotoxicity of both empty and dexamethasone‐loaded NPs prepared from tetramer OLGADMAs, we tested the viability of HeLa cells upon 24 and 48 h of incubation with aqueous NP suspensions using the Cell Counting Kit‐8 (CCK‐8) assay (**Figure** **14**). In brief, the assay uses a water‐soluble tetrazolium salt which is converted to a yellow formazan dye by action of cellular dehydrogenases. The amount of the developed formazan dye is directly proportional to the number of living cells in the sample. After 24 h of incubation with NP suspensions, the normalized cell viability was above 80% for all tested NPs and NP concentrations. Furthermore, no significant decrease in cell viability could be detected after 48 h of incubation with NP suspensions.

**Figure 15:**
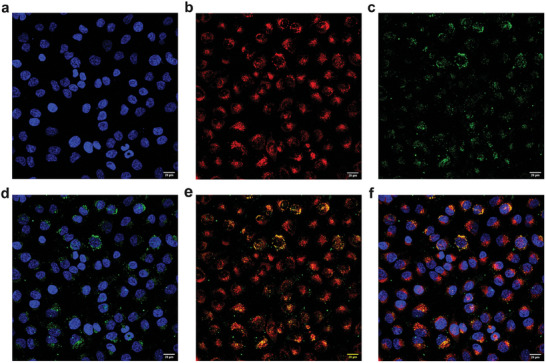
Uptake of fluorescein‐labeled **NP4Aa** in HeLa cells (40 µg mL^−1^), 40× magnification, scale bar: 20 µm. a) Hoechst, b) lysotracker red, c) NPs, d) Hoechst and NPs superimposed, e) lysotracker red and NPs superimposed, and f) Hoechst, lysotracker red and NPs superimposed.

We then incubated HeLa cells with fluorescent (fluorescein‐labeled) **NP4Aa** prepared by including 1 wt.% of fluorescein O‐methacrylate in the usual nanoprecipitation polymerization protocol and were able to verify the uptake of these NPs into these cells, with highly uniform distribution. As visible in **Figure** **15**, the fluorescein‐labeled **NP4Aa** (green) accumulated in the perinuclear region of the cells (blue), and displayed a relative colocalization with Lysotracker Red, a dye capable of staining acidic cellular compartments (e.g., endosomes and lysosomes).

## 3 Conclusion

In conclusion, we prepared novel polymerizable building blocks (OLGADMAs) inspired by PLGA and developed a rapid and surfactant‐free methodology to polymerize them into NPs. The main advantages of this technique are reflected in its ability to yield sub‐micron polymeric particles rapidly (e.g., within 15 min), without sonication requirements, and in the absence of stabilizers such as surfactants. The configuration of the method enables processes such as monomer polymerization, drug encapsulation, and NP functionalization (e.g., PEGylation) to occur simultaneously, in one pot. Furthermore, due to its simplicity, a facile translation of the nanoprecipitation‐polymerization technique toward automated methods for the preparation of polymeric NPs in a flexible and modular fashion can be envisaged. The methodology allows for the in situ encapsulation of dexamethasone, which was achieved with above‐average drug loading efficiencies (23–59%). We established a divergence in the nanopolymerization and drug release behavior of structurally diverse OLGADMAs and OLGADMA‐based NPs, which is suggestive of the existence of privileged sequences and structure‐property relationships. We carried out a comparative stability study against NPs prepared from three types of PLGA polymers and demonstrated a superior level of stability of OLGADMA‐based NPs at physiological temperature in water. We showed how a fraction of the encapsulated dexamethasone can be released in vitro from long‐lasting OLGADMA‐based NPs over 7 days and established the existence of a relationship between nanoparticle structure and cumulative drug release. Furthermore, we proved **DNP4Aa**, **DNP4Ab,** and **DNP4Bb** to exhibit good stability in biological medium, and an excellent cytotoxicity profile in vitro with storage at room temperature without significant size variations. We envisage the presented OLGADMA‐based NPs as potential drug nanocarriers with elevated loading capacity for hydrophobic small‐molecule drugs and high stability in aqueous environments. Drug‐loaded OLGADMA‐based NPs could find application for prolonged and long‐lasting drug delivery of small‐molecule therapeutics and as an efficient way of pseudo‐solubilization of poorly water‐soluble therapeutics. This would allow for the parenteral administration of substantial quantities of hydrophobic drug molecules, circumventing the need for chemical modifications of the drug or the use of toxic solubilizers such as surfactants. We envisage these NPs to enable long‐term delivery of small molecule therapeutics over several days to weeks or months, reducing dosing frequency and increasing patient compliance. Future works will focus on the investigation of the stability of OLGADMA‐based NPs over longer periods to establish the NP degradation products and ascertain the potential for full elimination of these species, especially with regards to the C‐C backbone resulting from the polymerization of the employed vinyl‐containing monomers.

## Conflict of Interest

The authors declare no conflict of interest.

## Supporting information



Supporting Information

## Data Availability

The data that support the findings of this study are available in the supplementary material of this article.
