# Supplementary material for: Long‐Lasting Cross‐Linked PLGA‐Inspired Nanoparticles from One‐Pot Nanopolymerization of Precisely Sequenced Short Oligolactoglycolic Acid Dimethacrylates
Source: Macromol Rapid Commun. 2025 Jan 21;46(8):2400778. doi: 10.1002/marc.202400778 (PMC12004898; doi:10.1002/marc.202400778)
Supplement: Supplementary file 1 — Supporting Information [file MARC-46-2400778-s001.docx]

*Supporting Information:*

**Long-lasting Cross-linked PLGA-Inspired Nanoparticles**

**from One-pot Nanopolymerization of Precisely Sequenced**

**Short Oligolactoglycolic Acid Dimethacrylates**

*Luka Blagojevic and Nazila Kamaly**

Department of Chemistry, Molecular Science Research Hub, Imperial College London, White City Campus, Wood Lane, W12 0BZ, London

Table of Contents

[S1. General procedures and instrumentation 3](#_Toc156839021)

[S2. Synthesis of acids 1 4](#_Toc156839022)

[S3. Synthesis of ethyl methacrylates 2 15](#_Toc156839023)

[S4. Synthesis of alcohols 3 15](#_Toc156839024)

[S5. Synthesis of OLGADMAs 15](#_Toc156839025)

[S6. Synthesis of OLGADMA-based Nanoparticles 16](#_Toc156839026)

[S7. Typical composition of nanoprecipitation polymerization reactions 17](#_Toc156839027)

[S8. Synthesis of PLGA-based Nanoparticles 17](#_Toc156839028)

[S9. Encapsulation Efficiency and Loading Efficiency Estimation 18](#_Toc156839029)

[S10. Dexamethasone Release at 37 °C 19](#_Toc156839030)

[S11. Stability of OLGADMA-based and PLGA-based Nanoparticles at 37 °C in Water 19](#_Toc156839031)

[S12. Stability of Dexamethasone-loaded Nanoparticles in Biological Media 19](#_Toc156839032)

[S13. Stability of Dexamethasone-loaded Nanoparticles During Storage at Room Temperature 20](#_Toc156839033)

[S14. Cytotoxicity *in vitro* 20](#_Toc156839034)

[S15. Fluorescence microscopy with HeLa cells 20](#_Toc156839035)

[S16. Characterization of compounds 22](#_Toc156839036)

[S17. ^1^H and ^13^C NMR spectra of OLGADMAs 57](#_Toc156839037)

[S18. Optimization of dexamethasone encapsulation 65](#_Toc156839038)

[S19. ATR-FTIR spectra of NPs 66](#_Toc156839039)

[S20. ^1^H NMR and DLS study of the nanoprecipitation polymerization reaction 68](#_Toc156839040)

[S21. Stability of dexamethasone under typical reaction conditions 69](#_Toc156839041)

[S22. ^1^H NMR analysis of released dexamethasone 70](#_Toc156839042)

[S23. Size-tunability of dexamethasone-loaded OLGADM-based NPs 71](#_Toc156839043)

[S24. Synthesis of PLGA alt 71](#_Toc156839044)

[S25. Dynamic Light Scattering (DLS) and Electrophoretic Light Scattering (ELS) 73](#_Toc156839045)

[S26. Intensity-weighted nanoparticle size distributions and correlation functions 74](#_Toc156839046)

[S27. Quantitation of dexamethasone 80](#_Toc156839047)

[S28. Transmission Electron Microscopy (TEM) 80](#_Toc156839048)

[S29. ζ-potential Analysis During Storage at Room Temperature 81](#_Toc156839049)

[S30. References 82](#_Toc156839050)

S1. General procedures and instrumentation

Commercial reagents were used as supplied. All non-aqueous reactions were carried-out under nitrogen gas atmosphere with flame-dried glassware, using standard techniques. Anhydrous solvents were obtained by filtration through drying columns (CH_2_Cl_2_ and THF) or used as supplied (EtOAc). Deionized water was obtained from a Sartorius Arium^®^ water purification system and was filtered through 0.2 µm Acrodisc^®^ syringe filters prior use. Deionized water used for nanoprecipitation polymerizations was deoxygenated by passing through nitrogen gas over 1 h. Nanoparticle suspensions were purified using Amicon^®^ Ultra-15 centrifugal filter units with a 30 kDa MWCO. Dexamethasone release was assayed in Amicon^®^ Ultra 0.5 centrifugal filter devices with a 100 kDa MWCO. Flash column chromatography was performed using 230–400 mesh silica, with the indicated solvent system according to standard techniques. Analytical thin-layer chromatography (TLC) was performed on precoated, aluminium silica gel sheets. Visualization of the developed chromatogram was performed by UV absorbance (254 nm) and staining with basic potassium permanganate stain. Infrared spectra (ν_max_, FTIR ATR) were recorded in reciprocal centimeters (cm^–1^) on an Agilent Cary 630 FTIR spectrometer. Nuclear magnetic resonance spectra were recorded on 400 MHz spectrometers. Chemical shifts for ^1^H NMR spectra are recorded in parts per million from tetramethylsilane with the solvent resonance as the internal standard (CHCl_3_: δ= 7.27 ppm, H_2_O: δ=4.79 ppm, DMSO: δ= 2.50 ppm). Data is reported as follows: chemical shift (multiplicity [s = singlet, d = doublet, t = triplet, q = quartet, quint = quintet, multiplet and br = broad], coupling constant (in Hz) and integration). ^13^C NMR spectra were recorded with complete proton decoupling. Chemical shifts are reported in parts per million from tetramethylsilane with the solvent resonance as the internal standard (^13^CDCl_3_: δ= 77.0 ppm). For clarity NMR spectra are displayed as follows: ^1^H NMR spectra are displayed between 10 ppm and 0 ppm; ^13^C NMR spectra are displayed between 210 ppm and 0 ppm. Quantitation of dexamethasone was performed by measurement of absorbance on an Agilent Cary 60 UV-Vis spectrophotometer. Particle size as z-average, PI and ζ-potential were determined by DLS analysis of aqueous suspensions performed at 25°C using a Malvern Zetasizer Ultra instrument. Nanoparticles were imaged directly using a JEM-2100Plus transmission electron microscope.

# S2. Synthesis of acids 1

Acids **1** were synthesized according to a previously reported multi-step synthetic procedure with minor modifications.^1^ A detailed description of the synthesis and characterization of acids **1** is presented in the following section.

#

**TBDPSO-(GL)_2_-COOH**

**
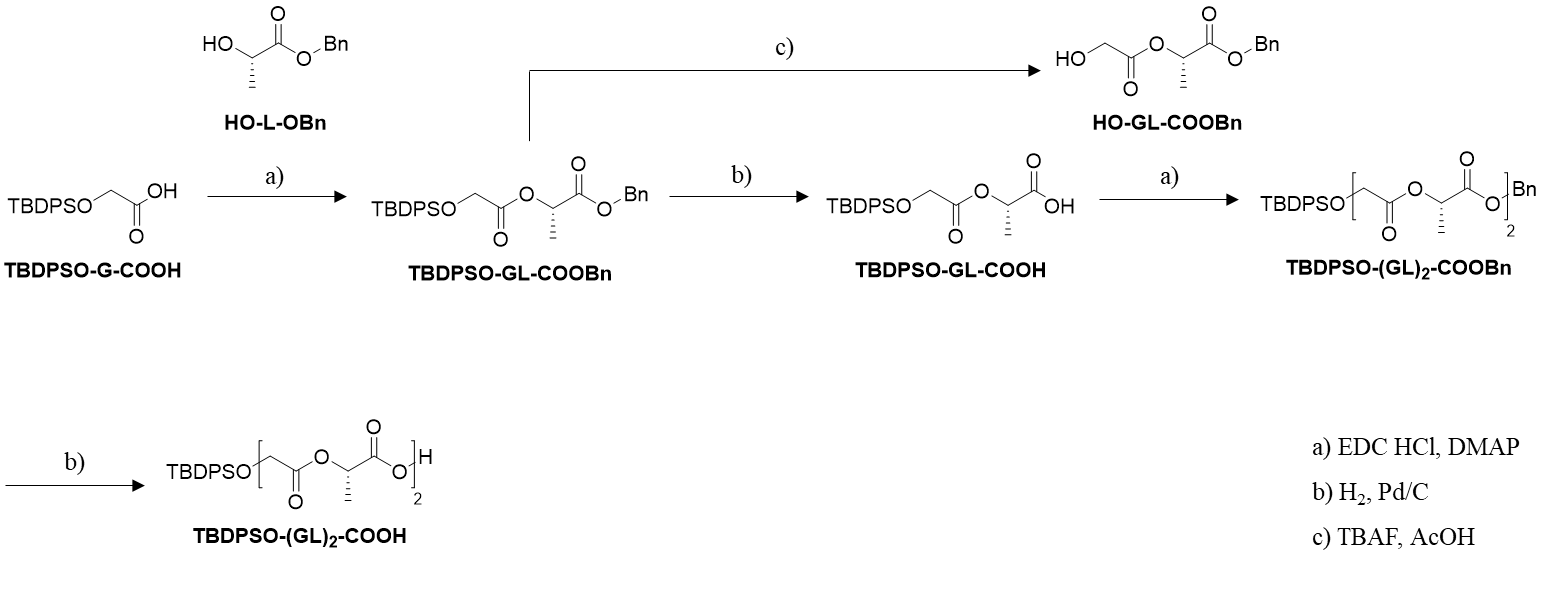
**

**TBDPSO-(LG)_2_-COOH**

**
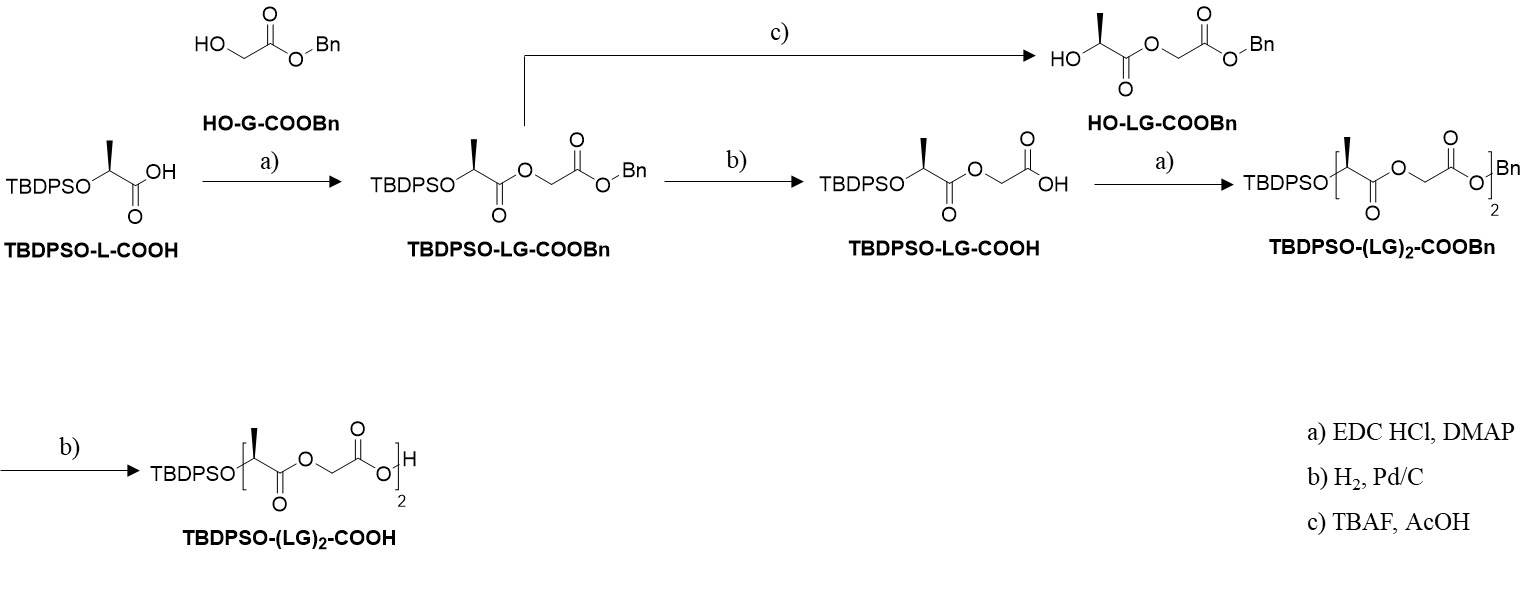
**

**TBDPSO-G_2_L_2_-COOH**

**
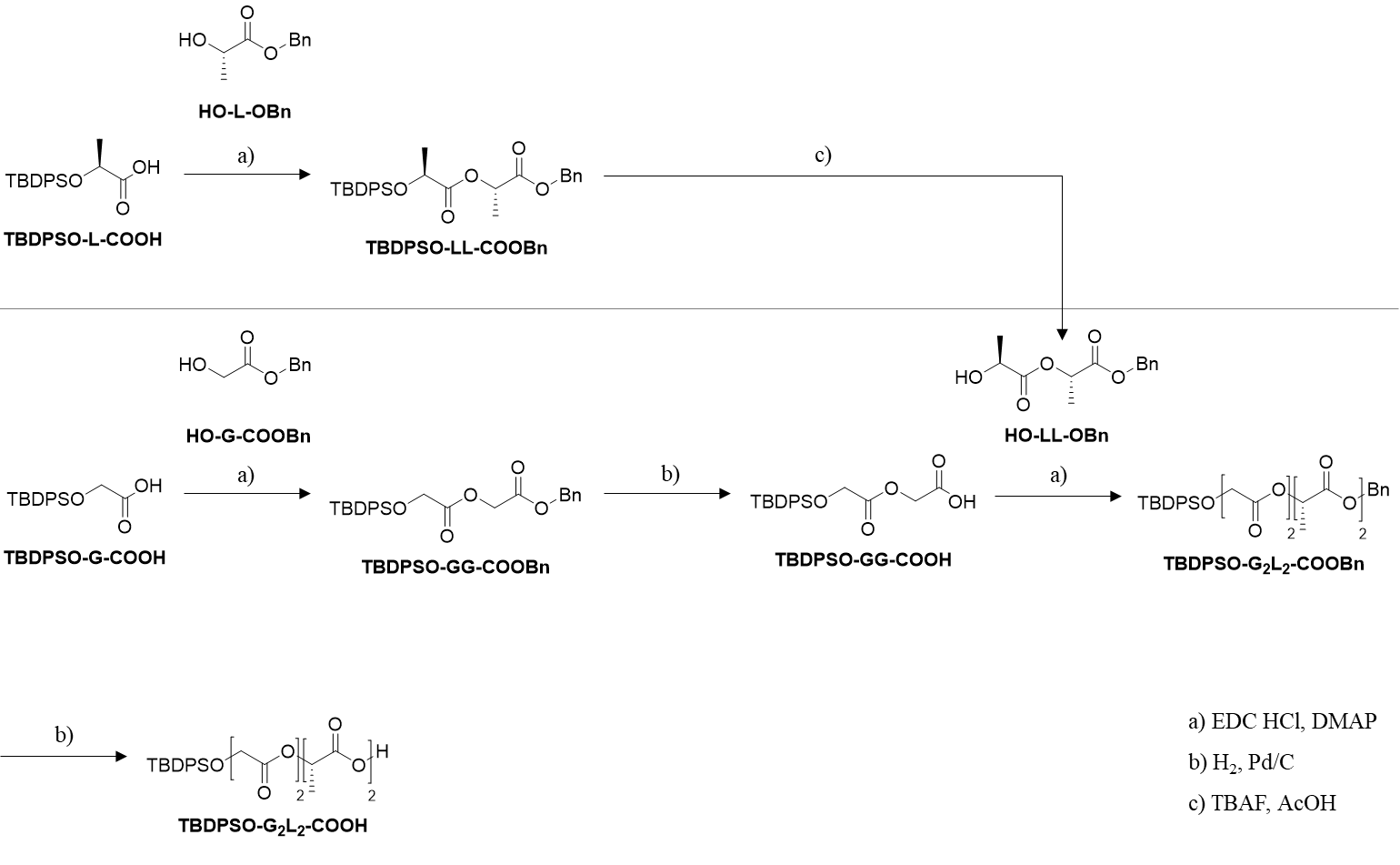
**

**TBDPSO-L_2_G_2_-COOH**

**
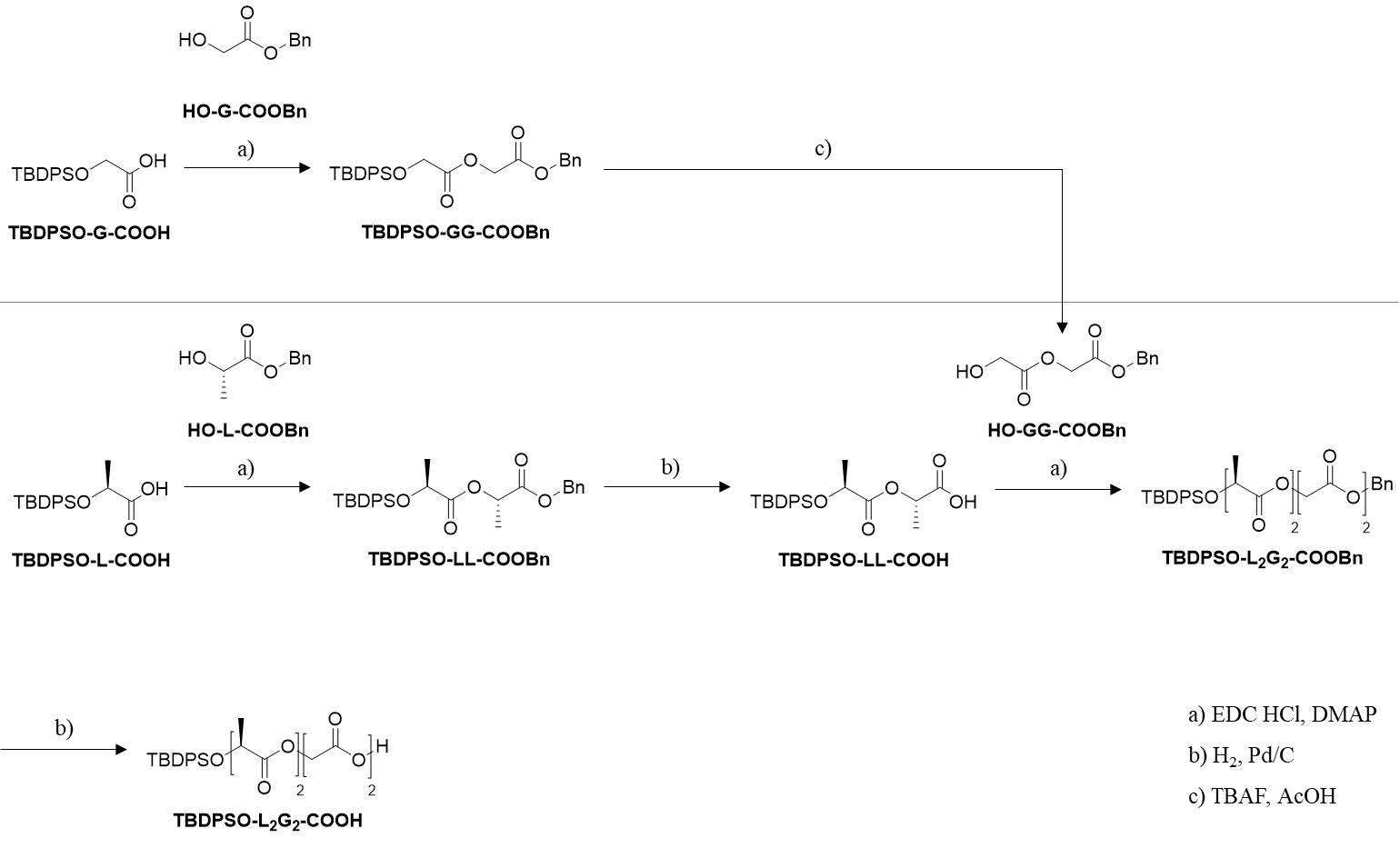
**

**TBDPSO-(LG)_3_-COOH**

**
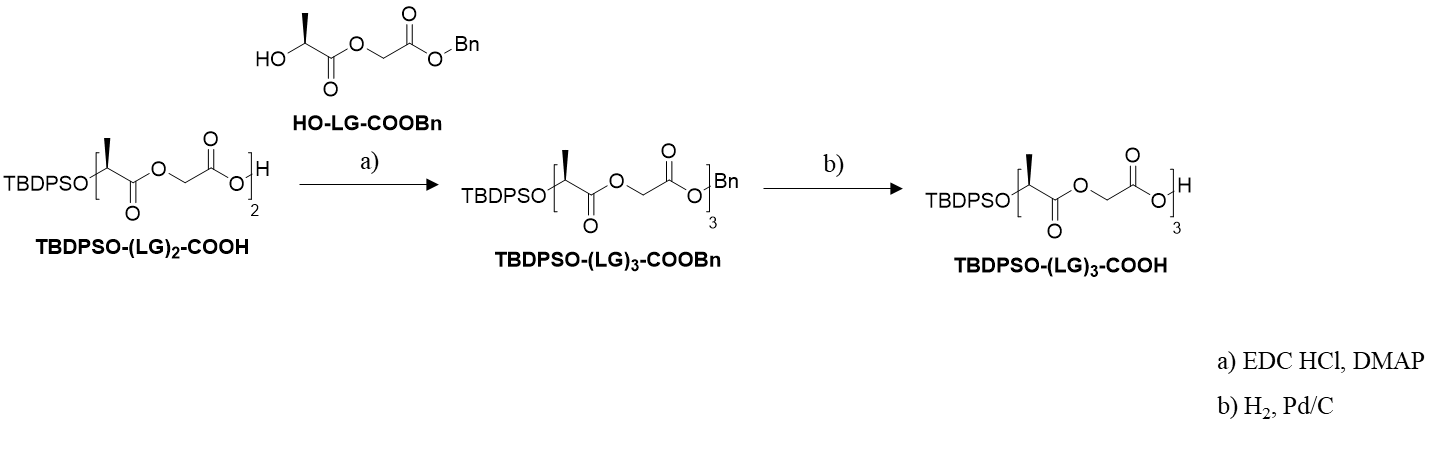
**

**TBDPSO-G_3_L_3_-COOH**

**
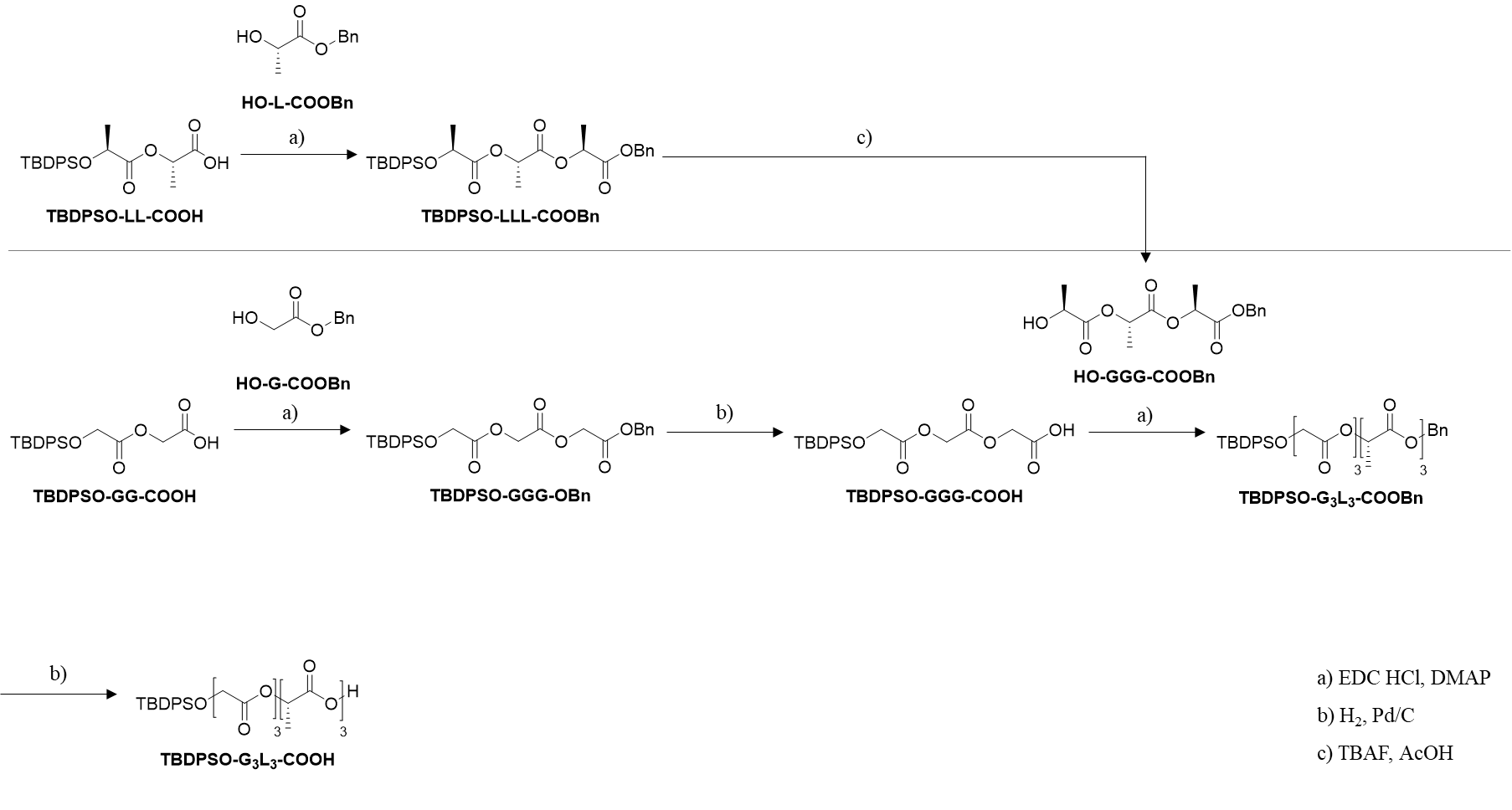
**

**TBDPSO-(LG)_4_-COOH**

**
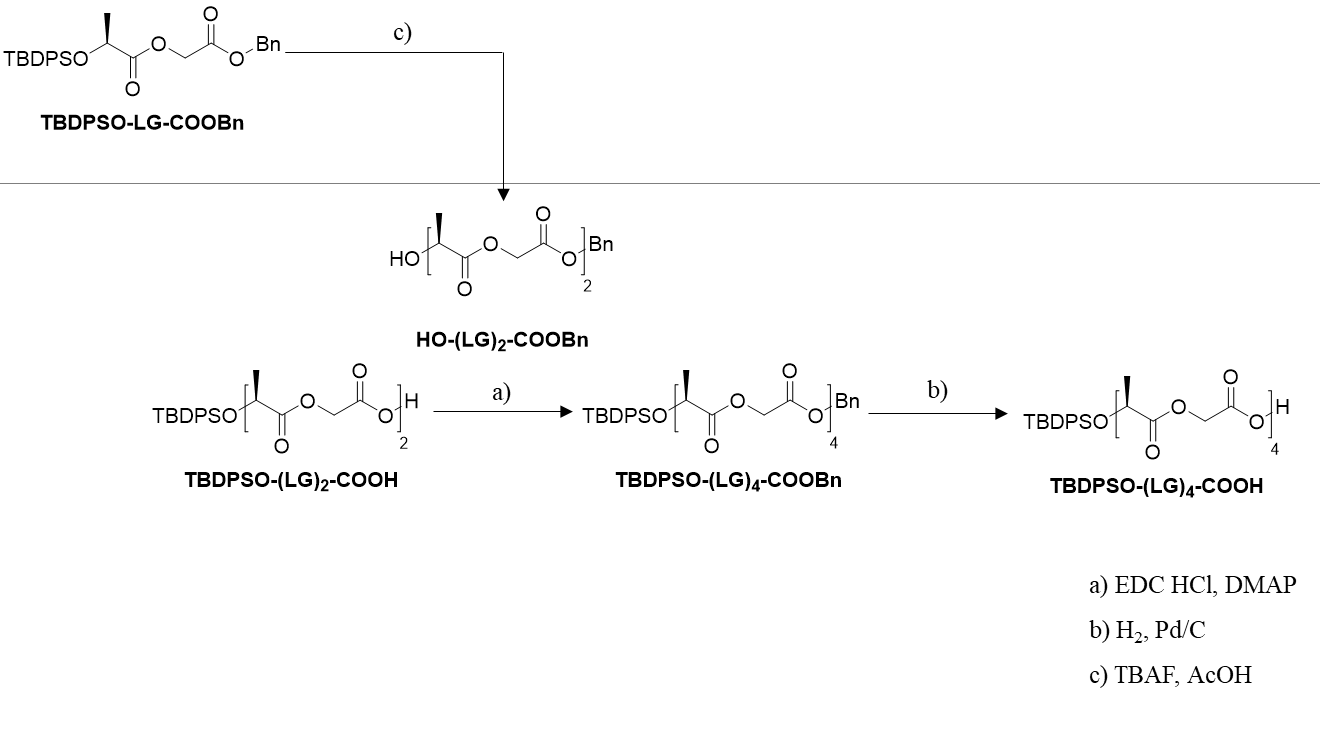
**

**TBDPSO-L_4_G_4_-COOH**

**
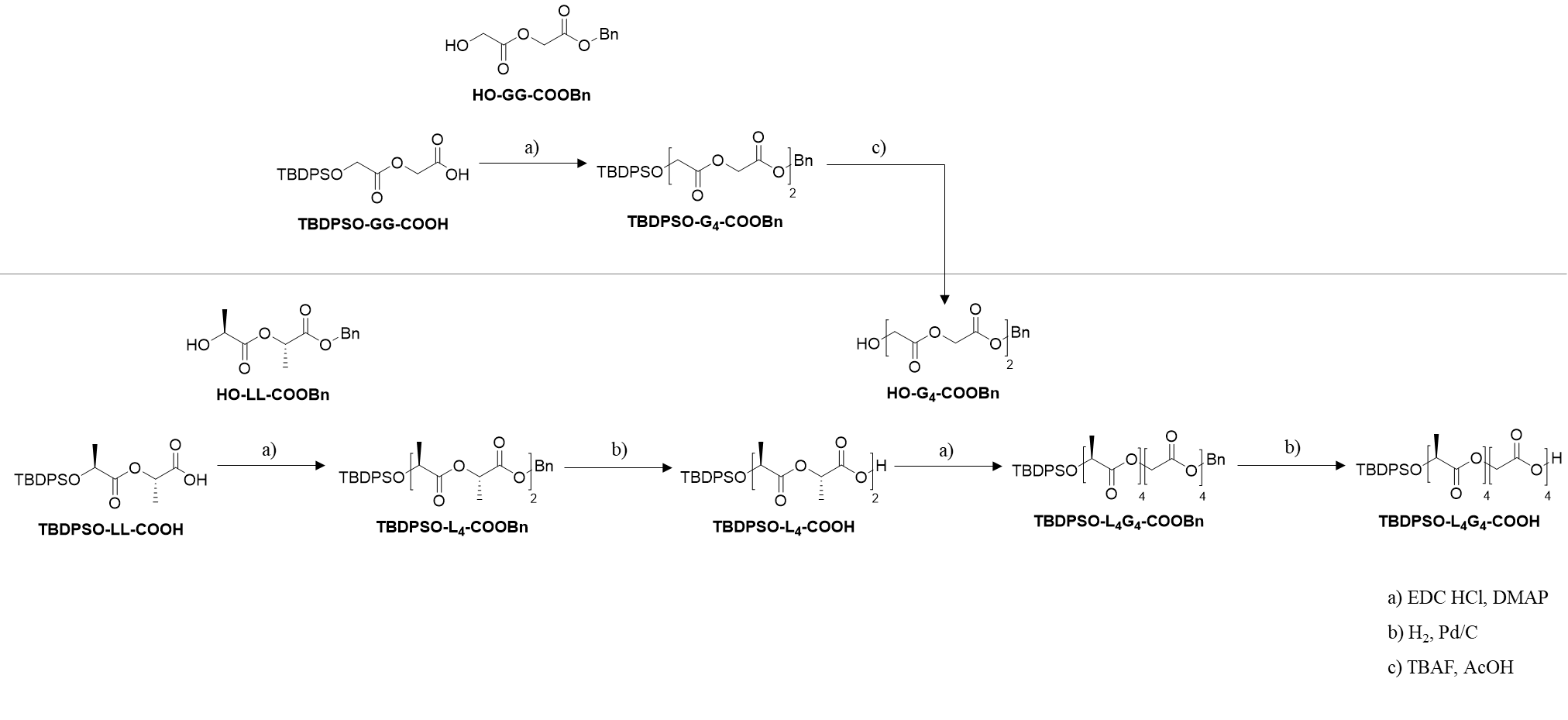
**

**HO-G-COOBn**. DBU (0.17 mL, 1.1 mmol) was added dropwise to a mixture of glycolic acid (0.09 g, 1.2 mmol) and benzyl bromide (0.12 mL, 1.0 mmol) in acetonitrile (1 mL) at 0 °C. The reaction mixture was warmed to 70 °C, left stirring for 5h and then poured into ice cold water (10 mL). The aqueous phase was extracted with ethyl acetate (3 × 10 mL) and the combined organic extracts washed with aq. HCl (1M, 10 mL), ice cold water (2 × 10 mL), dried over Na_2_SO_4_ and concentrated *in vacuo* to afford the desired compound.

**HO-L-COOBn**. DBU (3.30 mL, 22.0 mmol) was added dropwise to a mixture of *L*-lactic acid (>85% solution in water, 2.5 g, >24 mmol) and benzyl bromide (2.38 mL, 20.0 mmol) in acetonitrile (20 mL) at 0 °C. The reaction mixture was warmed to 70 °C, left stirring for 2 h 45 min and then poured into ice cold water (20 mL). The aqueous phase was extracted with ethyl acetate (2 × 20 mL) and the combined organic extracts washed with aq. HCl (10%, 20 mL), brine (20 mL), dried over Na_2_SO_4_ and concentrated *in vacuo*. The crude mixture was purified by vacuum distillation (165 °C at 0.22 mmHg) to afford the desired compound.

**TBDPSO-G-COOH**. TBDPS chloride (6.24 mL, 24.0 mmol) was added dropwise to a solution of methyl glycolate (1.54 mL, 20.0 mmol) and imidazole (2.7 g, 40 mmol) in dichloromethane (80 mL) at 0°C. The reaction mixture was warmed to rt, left stirring for 2h and then quenched by addition of aq. HCl (1 M, 50 mL). The organic phase was washed with water (2 × 100 mL), dried over Na_2_SO_4_ and concentrated *in vacuo*. The resulting crude mixture was dissolved in THF (180 mL) and aq. NaOH (1 M, 80 mL, 80 mmol) was added to the solution at 0°C. The mixture was then warmed to rt and stirred for 4 h. Water (100 mL) was added upon reaction completion and the THF removed by concentration *in vacuo.* The aqueous phase was washed with diethyl ether (2 × 100 mL), acidified with aq. HCl (1 M, 50 mL) until reaching pH 2 and extracted with ethyl acetate (3 × 50 mL). The organic phase was dried over Na_2_SO_4_ and concentrated *in vacuo* to afford the desired compound.

**TBDPSO-L-COOH**. TBDPS chloride (6.24 mL, 24.0 mmol) was added dropwise to a solution of methyl *L*-lactate (1.91 mL, 20.0 mmol) and imidazole (2.72 g, 40.0 mmol) in dichloromethane (80 mL) at 0 °C. The reaction mixture was warmed to rt, left stirring for 1 h and then quenched by addition of 10% aq. HCl (50 mL). The organic phase was washed with brine (2 × 50 mL), dried over Na_2_SO_4_ and concentrated *in vacuo*. The resulting crude mixture was dissolved in THF (100 mL), aq. NaOH (1 M, 80 mL, 80 mmol) was added and the mixture was stirred 32 h at rt. Upon completion, water (100 mL) was added and the THF removed by concentration *in vacuo.* The aqueous phase was washed with diethyl ether (2 × 100 mL), acidified with 10% aq. HCl (50 mL) to reach pH 2 and extracted with ethyl acetate (3 × 50 mL). The organic phase was dried over Na_2_SO_4_ and concentrated *in vacuo* to afford the desired compound.

**General procedure for ester synthesis (a)**. EDC HCl (1.3 equiv) was added to a solution of acid (1 equiv), alcohol (1.2 equiv) and DMAP (0.5 equiv) in dichloromethane. The mixture was left stirring overnight at rt and then concentrated *in vacuo*. Purification by flash chromatography (ethyl acetate/hexane) afforded the desired compounds.

**General procedure for catalytic hydrogenation (b).** A mixture of benzyl ester and Pd/C (5 wt%) in ethyl acetate was left stirring under hydrogen gas (atmospheric pressure) overnight. The system was then purged with nitrogen gas and the mixtures filtered through a short plug of celite, eluting with dichloromethane. The resulting solution was concentrated *in vacuo* to afford the desired acids.

**General procedure for silyl ether cleavage (c).** TBAF (1.5 equiv) was added to a cold solution of silyl ether and glacial acetic acid (3 equiv) in THF and left stirring at 0 °C. The reaction was monitored by TLC, warming to room temperature if necessary. Upon completion, the reaction mixture was mixed with brine, extracted three times with diethyl ether, dried over Na_2_SO_4_ and concentrated *in vacuo*. Purification by flash chromatography (ethyl acetate/hexane or diethyl ether/hexane) afforded the desired alcohols.

# S3. Synthesis of ethyl methacrylates 2

EDC HCl (1.3 equiv) was added to a solution of **1** (1 equiv), HEMA (1.3 equiv) and DMAP (0.5 equiv) in dichloromethane. The mixture was left stirring overnight at rt and then concentrated *in vacuo*. Purification by flash chromatography (ethyl acetate/hexane) afforded the desired compounds **2**.

# S4. Synthesis of alcohols 3

TBAF (1.5 equiv) was added to a cold solution of **2** and glacial acetic acid (3 equiv) in THF and left stirring at 0 °C. The reaction was monitored by TLC, warming to room temperature if necessary. Upon completion, the reaction mixture was mixed with brine, extracted three times with diethyl ether, dried over Na_2_SO_4_ and concentrated *in vacuo*. Purification by flash chromatography (ethyl acetate/hexane or diethyl ether/hexane) afforded the desired compounds **3**.

# S5. Synthesis of OLGADMAs

Methacryloyl chloride (2 or 3 equiv) was added to a cold solution of **3** and triethylamine (4 equiv) in dichloromethane at 0 °C. The reaction was monitored by TLC, warming to room temperature if necessary. Upon completion, the reaction mixture was partitioned between brine and dichloromethane, extracted with dichloromethane, dried over Na_2_SO_4_ and concentrated *in vacuo*. Purification by flash chromatography (ethyl acetate/hexane) afforded the desired **OLGADMAs**.

# S6. Synthesis of OLGADMA-based Nanoparticles

OLGADMA-based nanoparticles have been prepared by quick injection of a THF solution of either a) OLGADM and PEGDA700 (1:1 weight ratio) or b) OLGADMA, PEGDA700 and dexamethasone (1:1:0.75 weight ratio) and AIBN (20 wt% of total reagents) into water at 70 ºC, stirring at 1000 rpm, with the system open to air and under N_2_ gas flow. The reaction mixture was immediately purged with N_2_ gas for 5 minutes and then left under N_2_ gas atmosphere for a further 10 min. The reaction mixtures were then exposed to air and cooled in an ice bath for 2 minutes. The resulting suspensions were purified by centrifugal filtration (MWCO 30 kDa), *via* two centrifugations at 3200 rcf for 5 minutes, passing through water (12 mL). NP suspensions were obtained by diluting the retentate (< 0.5 mL) to a final volume of 5 mL with deionized water, mixed manually to achieve re-suspension, and stored at 4 ºC. Examples of typical reaction compositions are reported in the supplementary information section.

*Safety note*: We acknowledge the potential hazards deriving from a quick injection of substantial quantities of an organic solution of AIBN (H242) into water at 70 °C. We did not observe any unwanted events in any of the reactions. The highest amount of AIBN used in this work was 4 mg per reaction.

# S7. Typical composition of nanoprecipitation polymerization reactions

OLGADMA-based nanoparticles and dexamethasone-loaded OLGADMA-based nanoparticles presented in this work were synthesized via nanoprecipitation polymerizations having the compositions presented in Table S1.

| Component | OLGADM-based NPs | Dexamethasone-loaded OLGADM-based NPs |
| --- | --- | --- |
| OLGADM (mg) | 8 | 8 |
| PEGDA700 (mg) | 8 | 8 |
| Dexamethasone (mg) | - | 6 |
| AIBN (mg) | 4 | 4 |
| THF (mL) | 1.6 | 1.6 |
| H_2_O (mL) | 4.8 | 4.8 |

**Table S1.** Typical composition of nanoprecipitation polymerization reactions.

# S8. Synthesis of PLGA-based Nanoparticles

PLGA-based nanoparticles were prepared by quick injection of a THF solution of either a) PLGA-based polymer or b) PLGA-based polymer and dexamethasone (1:0.375 weight ratio) into water at 70 ºC, stirring at 1000 rpm, with the system open to air and under N_2_ gas flow. The reaction mixture was immediately purged with N_2_ gas for 5 minutes and then left under N_2_ gas atmosphere for a further 10 minutes. The mixtures were then exposed to air and cooled in an ice bath for 2 minutes. The crude mixtures were subjected to identical purification and storage conditions described above.

# S9. Encapsulation Efficiency and Loading Efficiency Estimation

The encapsulation efficiency of nanoparticles was estimated by quantitation of un-encapsulated dexamethasone and subtraction of this value from the dexamethasone feed according to the following equation:

$$\%EE =\frac{un-encapsulated dexamethasone mass}{dexamethasone feed mass} \times100$$

Un-encapsulated dexamethasone was quantified spectrophotometrically from methanol/water solutions (1:1 volume ratio) obtained by combining the filtrates from ultrafiltration step and methanol solutions obtained by washing the reaction vial and filters used for the ultrafiltration. The loading efficiency of nanoparticles was determined as the ratio of the concentration of dexamethasone and the mass of the solids obtained after 48 h of freeze-drying of nanoparticle suspensions.

$$\%LE =\frac{mass of dexamethasone in 1 mL of suspension}{mass of solids obtained from 1 ml of suspension} \times100$$

# S10. Dexamethasone Release at 37 °C

Dexamethasone-containing nanoparticle suspensions (400 µL) were placed into ultrafiltration filters (MWCO 100 kDa), sealed with parafilm and incubated at 37 ºC. The samples were then centrifuged (14000 rcf for 5 minutes at 37 ºC) at determined time points and the retentate was collected for dexamethasone quantitation. The content of the filter was then re-suspendedthe by addition of water (400 µL), re-sealed, sonicated in a bath sonicator (2 min) and re-incubated at 37 ºC. The released dexamethasone was quantified from the individual filtrates and cumulative release curves plotted against time.

S11. Stability of OLGADMA-based and PLGA-based Nanoparticles at 37 °C in Water

Nanoparticle stability in water at 37 ºC was studied by examination of hydrodynamic size, polydispersity, ζ-potential, formulation pH and FTIR over a period of 5 weeks. Typically, OLGADMA/PEGDA700 and PLGA-based nanoparticle suspensions (2 mL) were placed in test tubes, sealed with parafilm, incubated at 37 ºC and left stirring at 500 rpm. At determined time points, samples (300 µL) were collected for DLS and pH analysis, then freeze-dried for FTIR analysis.

# S12. Stability of Dexamethasone-loaded Nanoparticles in Biological Media

Dexamethasone-containing nanoparticle suspensions (20 µL) were mixed with the dispersant (900 µL of either water, DMEM, and FBS-supplemented DMEM) and left to equilibrate at room temperature for 15 min. The samples were then incubated at 37 ºC for 2 hours and analyzed by DLS size and PI. Prior to DLS analysis, the samples containing FBS were centrifuged (15000 rcf at room temperature for 20 minutes), the supernatant discarded, and the sediment re-suspended in FBS-free media (900 µL) by simple inversion of the centrifuge tube.

# S13. Stability of Dexamethasone-loaded Nanoparticles During Storage at Room Temperature

Samples of dexamethasone-loaded nanoparticle stock dispersions (100 µL) were suspended in deionized water (1.5 mL), sealed in vials, and left to equilibrate at room temperature for 2 h. The samples were at room temperature and monitored by DLS (size, PI and ζ-potential) at determined time points.

# S14. Cytotoxicity *in vitro*

HeLa cells were seeded in 96-well plates (5 × 103 cells per well) in 100 μL of DMEM supplemented with 10% fetal bovine serum (FBS), 1% penicillin and streptomycin, and incubated for 24 h at 37 °C and 5% CO_2_. The media was then removed, fresh media was added (90 μL), and the cells were treated with aliquots (10 μL) of aqueous suspensions of **NP4Aa**, **NP4Ab**, and **NP4Bb** with NP concentrations of 0.5, 0.25, 0.1, 0.05, and 0.01 mg/mL. Control cells were treated with water (10 μL). The cells were then incubated for 24 h and 48 h at 37 °C and 5% CO_2_. The cell viability was measured using the Cell Counting Kit-8 (CCK-8) assay, according to the manufacturer’s instructions, assigning 100% viability to cells treated with water.

# S15. Fluorescence microscopy with HeLa cells

Fluorescein-labelled NP4Aa were prepared according to the standard nanoprecipitation polymerization and purification protocol, with the addition of fluorescein O-methacrylate (1 wt. % of monomers). NPs had a z-average = 272 ± 6.6 nm, PI = 0.20 ± 0.06, ζ-potential (deionized water, 25 °C) = -27.68 ± 1.2 mV. The cellular uptake of fluorescein-labelled NP4Aa was investigated using HeLa cells, which were seeded into an 8-well chambered coverslip for cell imaging (5 × 10^4^ cells per chamber) in 300 μL of DMEM supplemented with 10% FBS and incubated at 37 °C and 5% CO2. The media was removed after 48 h of incubation and 300 μL of OptiMEM was added to each chamber, followed by the addition of an aqueous suspension of fluorescent NP4Aa to achieve a NP content of 40, 60, and 100 μg/mL per chamber. The cells were then incubated at 37 °C and 5% CO2 for 6 h after which the media was removed, the cells washed fourfold with sterile PBS and treated with Lysotracker Red (75 nM) for 30 min at 37 °C. Then, the cells were washed thrice with PBS and fixed with 300 μL of a 4% paraformaldehyde solution for 15 min. The paraformaldehyde solution was then removed, the cells washed twice with PBS and stained with Hoechst stain for 15 min in the dark. The stain was then removed, the cells were washed twice with PBS, and stored in sterile PBS until imaging. The cells were imaged at 40x on a Leica SP8 DLS system using the oil immersion technique.

# S16. Characterization of compounds

**
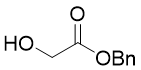
**

**HO-G-COOBn**

**Appearance:** colorless oil; **Yield:** 76%; R*_f_* = 0.23 (50% Et_2_O/hexane); IR (film)/cm^-1^ 3451 (br. OH), 1736 (C=O), 1498, 1453, 1192, 1084, 991, 738, 697; The observed spectroscopic data (^1^H NMR and ^13^C NMR) for this compound was consistent with that previously reported.^1^


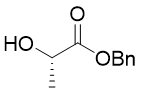


**HO-L-COOBn**

**Appearance:** colorless oil; **Yield:** 41%; R*_f_* = 0.69 (50% ethyl acetate/hexane); IR (film)/cm^-1^ 3462 (br. OH), 2982, 1737 (C=O), 1498, 1453, 1375, 1264, 1215, 1126, 1043, 954, 753, 701; The observed spectroscopic data (^1^H NMR and ^13^C NMR) for this compound was consistent with that previously reported.^1^


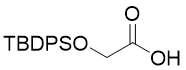


**TBDPSO-G-COOH**

**Appearance:** colorless oil; **Yield:** 55%; IR (film)/cm^-1^ 3071, 2929, 2855, 1726 (s. C=O), 1587, 1472, 1427, 1390, 1364, 1244, 1136, 1110, 998, 939, 820, 738, 697. The observed spectroscopic data (^1^H NMR and ^13^C NMR) was consistent with that previously reported.^1^

**
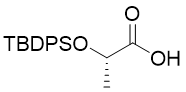
**

**TBDPSO-L-COOH**

**Appearance:** white solid; MP: 69-72 °C; **Yield:** 59%; IR (film)/cm^-1^ 3071, 2933, 2892, 2859, 1722 (s. C=O), 1472, 1427, 1364, 1300, 1244, 1140, 1110, 1058, 969, 823, 786, 738, 701. The observed spectroscopic data (^1^H NMR and ^13^C NMR) was consistent with that previously reported.^1^

**TBDPSO-GL-COOBn**

**Appearance:** colorless oil**, Yield:** 62%**, Characterization:** R*_f_* = 0.18 (5% EtOAc/hexane); IR (film)/cm^-1^ 3041, 2937, 2855, 1751 (C=O), 1453, 1267, 1192, 1133, 820, 700, 506; ^1^H NMR (400 MHz, CDCl_3_) δ 7.73 – 7.67 (m, 4H), 7.48 – 7.31 (m, 11H), 5.24 – 5.14 (m, 3H), 4.37 (d, *J* = 16.8 Hz, 1H), 4.31 (d, *J* = 16.7 Hz, 1H), 1.46 (d, *J* = 7.1 Hz, 3H), 1.10 (s, 9H); ^13^C NMR (101 MHz, CDCl_3_) δ 170.6, 170.3, 135.6, 135.5, 135.2, 132.7, 129.9, 128.6, 128.4, 128.1, 127.8, 68.7, 67.0, 62.0, 26.6, 19.2, 16.9.

**TBDPSO-LG-COOBn**

**Appearance:** colorless oil**, Yield:** 74%**, Characterization:** R*_f_* = 0.30 (5% EtOAc/hexane); IR (film)/cm^-1^ 3071, 2933, 2858, 1759 (C=O), 1427, 1394, 1177, 1133, 969, 782, 741, 700; ^1^H NMR (400 MHz, CDCl_3_) δ 7.72 – 7.65 (m, 4H), 7.48 – 7.28 (m, 11H), 5.18 (s, 2H), 4.62 (d, *J* = 15.9 Hz, 1H), 4.47 (d, *J* = 15.9 Hz, 1H), 4.39 (q, *J* = 6.8 Hz, 1H), 1.41 (d, *J* = 6.8 Hz, 3H), 1.10 (s, 9H); ^13^C NMR (101 MHz, CDCl_3_) δ 173.1, 167.3, 135.9, 135.7, 135.0, 133.4, 133.0, 129.8, 128.6, 128.5, 128.4, 127.6, 127.6, 68.7, 67.0, 60.6, 26.8, 21.2, 19.2; HRMS (TOF MS ES+) m/z calcd for C_28_H_32_O_5_NaSi^+^ [M+Na]^+^: 499.1917; Found: 499.1909.

**TBDPSO-GG-COOBn**

**Appearance:** colorless oil**, Yield:** 78%**, Characterization:** R*_f_* = 0.15 (5% EtOAc/hexane); IR (film)/cm^-1^ 3071, 2929, 2855, 1755 (C=O), 1472, 1427, 1267, 1133, 823, 738, 697; ^1^H NMR (400 MHz, CDCl_3_) δ 7.73 – 7.67 (m, 4H), 7.48 – 7.32 (m, 11H), 5.20 (s, 2H), 4.68 (s, 2H), 4.37 (s, 2H), 1.10 (s, 9H); ^13^C NMR (101 MHz, CDCl_3_) δ 170.6, 167.3, 135.5, 135.0, 134.8, 132.6, 129.9, 128.6, 128.5, 128.4, 127.8, 67.1, 61.9, 60.7, 26.6, 19.2; HRMS (TOF MS ES+) m/z calcd for C_27_H_30_O_5_NaSi^+^ [M+Na]^+^: 485.1760; Found: 485.1742.

**TBDPSO-LL-COOBn**

**Appearance:** colorless oil**, Yield:** 57%**, Characterization:** R*_f_* = 0.20 (5% EtOAc/hexane); IR (film)/cm^-1^ 3071, 2933, 2858, 1751 (C=O), 1587, 1453, 1427, 1267, 1185, 1129, 972, 823, 738, 700; ^1^H NMR (400 MHz, CDCl_3_) δ 7.73 – 7.64 (m, 4H), 7.50 – 7.27 (m, 11H), 5.17 (d, *J* = 12.3 Hz, 1H), 5.11 (d, *J* = 12.3 Hz, 1H) 4.99 (q, *J* = 7.1 Hz, 1H), 4.33 (q, *J* = 6.7 Hz, 1H), 1.38 (d, *J* = 6.7 Hz, 3H), 1.34 (d, *J* = 7.1 Hz, 3H), 1.10 (s, 9H); ^13^C NMR (101 MHz, CDCl_3_) δ 173.1, 170.3, 135.9, 135.7, 135.2, 133.4, 133.1, 129.8, 128.6, 128.4, 128.2, 127.6, 127.5, 68.5, 67.0, 26.8, 21.1, 19.2, 16.7; HRMS (TOF MS ES+) m/z calcd for C_29_H_34_O_5_SiNa^+^ [M+Na]^+^: 513.2073; Found: 513.2070.

**TBDPSO-GGG-COOBn**

**Appearance:** colorless oil**, Yield:** 20%**, Characterization:** R*_f_* = 0.40 (30% EtOAc/hexane); IR (film)/cm^-1^ 3071, 2929, 2855, 1755 (C=O), 1587, 1423, 1394, 1267, 1170, 1133, 1073, 823; ^1^H NMR (400 MHz, CDCl_3_) δ 7.73 – 7.66 (m, 4H), 7.49 – 7.32 (m, 11H), 5.21 (s, 2H), 4.75 (s, 2H), 4.73 (s, 2H), 4.37 (s, 2H), 1.10 (s, 9H); ^13^C NMR (101 MHz, CDCl_3_) δ 170.5, 166.9, 135.5, 134.9, 132.6, 129.9, 128.7, 128.6, 128.4, 127.8, 67.3, 61.8, 61.1, 60.3, 26.6, 19.2; HRMS (TOF MS ES+) m/z calcd for C_29_H_32_O_7_NaSi^+^ [M+Na]^+^: 543.1815; Found: 543.1811.

**TBDPSO-LLL-COOBn**

**Appearance:** colorless oil**, Yield:** 91%**, Characterization:** R*_f_* = 0.23 (10% EtOAc/hexane); IR (film)/cm^-1^ 3068, 2930, 2856, 1750 (C=O), 1588, 1452, 1184, 1127, 1091, 971, 738, 698; ^1^H NMR (400 MHz, CDCl_3_) δ 7.73 – 7.64 (m, 4H), 7.53 – 7.28 (m, 11H), 5.22 – 5.10 (m, 3H), 4.95 (q, *J* = 7.1 Hz, 1H), 4.33 (q, *J* = 6.7 Hz, 1H), 1.51 (d, *J* = 7.1 Hz, 3H), 1.42 (d, *J* = 6.7 Hz, 3H), 1.34 (d, *J* = 7.1 Hz, 3H), 1.10 (s, 9H); ^13^C NMR (101 MHz, CDCl_3_) δ 173.1, 170.1, 169.9, 136.0, 135.7, 135.1, 133.4, 133.1, 129.8, 128.6, 128.5, 128.2, 127.6, 127.6, 69.1, 68.5, 68.2, 67.1, 26.8, 21.1, 19.2, 16.8, 16.5; HRMS (TOF MS ES+) m/z calcd for C_32_H_38_O_7_NaSi^+^ [M+Na]^+^: 585.2285; Found: 585.2293.

**TBDPSO-(GL)_2_-COOBn**

**Appearance:** colorless oil**, Yield:** 84%**, Characterization:** R*_f_* = 0.26 (20% EtOAc/hexane); IR (film)/cm^-1^ 3041, 2987, 2855, 1751 (C=O), 1453, 1267, 1177, 1088, 820, 700; ^1^H NMR (400 MHz, CDCl_3_) δ 7.73 – 7.67 (m, 4H), 7.48 – 7.31 (m, 11H), 5.26 – 5.14 (m, 4H), 4.87 (d, *J* = 16.0 Hz, 1H), 4.63 (d, *J* = 16.0 Hz, 1H), 4.38 (d, *J* = 16.8 Hz, 1H), 4.31 (d, *J* = 16.8 Hz, 1H), 1.53 (d, *J* = 7.1 Hz, 3H), 1.53 (d, *J* = 7.1 Hz, 3H), 1.09 (s, 9H); ^13^C NMR (101 MHz, CDCl_3_) δ 170.6, 169.8, 166.6, 135.6, 135.5, 135.1, 132.7, 129.9, 128.6, 128.5, 128.2, 127.8, 69.5, 68.4, 67.2, 61.9, 60.7, 26.6, 19.2, 16.8, 16.8.

**TBDPSO-(LG)_2_-COOBn**

**Appearance:** colorless oil**, Yield:** 89%**, Characterization:** R*_f_* = 0.24 (15% EtOAc/hexane); IR (film)/cm^-1^ 3069, 2933, 2857, 1753 (C=O), 1451, 1424, 1277, 1174, 1128, 1101, 969, 740, 701, 611, 507; ^1^H NMR (400 MHz, CDCl_3_) δ 7.71 – 7.65 (m, 4H), 7.49 – 7.31 (m, 11H), 5.24 (q, *J* = 7.1 Hz, 1H), 5.19 (s, 2H), 4.80 (d, *J* = 15.9 Hz, 1H), 4.68 (d, *J* = 16.0 Hz, 1H), 4.62 (d, *J* = 15.9 Hz, 1H), 4.45 (d, *J* = 16.0 Hz, 1H), 4.38 (q, *J* = 6.8 Hz, 1H), 1.53 (d, *J* = 7.1 Hz, 3H), 1.43 (d, *J* = 6.8 Hz, 3H), 1.10 (s, 9H); ^13^C NMR (101 MHz, CDCl_3_) δ 173.0, 169.5, 166.9, 166.8, 135.9, 135.7, 134.8, 133.4, 132.9, 129.8, 128.7, 128.5, 127.7, 127.6, 69.0, 68.6, 67.3, 61.1, 60.1, 26.8, 21.3, 19.2, 16.7; HRMS (TOF MS ES+) m/z calcd for C_33_H_38_O_9_NaSi^+^ [M+Na]^+^: 629.2183; Found: 629.2181.

**TBDPSO-G_2_L_2_-COOBn**

**Appearance:** colorless oil**, Yield:** 97%**, Characterization:** R*_f_* = 0.26 (20% EtOAc/hexane); IR (film)/cm^-1^ 3069, 2931, 2857, 1752 (C=O), 1451, 1173, 970; ^1^H NMR (400 MHz, CDCl_3_) δ 7.74 – 7.67 (m, 4H), 7.47 – 7.33 (m, 11H), 5.25 – 5.13 (m, 4H), 4.77 (d, *J* = 16.1 Hz, 1H), 4.66 (d, *J* = 16.1 Hz, 1H), 4.37 (d, *J* = 16.8 Hz, 1H), 4.37 (d, *J* = 16.8, 1H), 1.54 (d, *J* = 7.1 Hz, 3H), 1.52 (d, *J* = 7.1 Hz, 3H), 1.10 (s, 9H); ^13^C NMR (101 MHz, CDCl_3_) δ 170.5, 169.9, 169.5, 166.9, 135.5, 135.0, 132.6, 132.6, 129.9, 128.6, 128.5, 128.2, 127.8, 69.3, 69.1, 67.2, 61.8, 60.4, 26.6, 19.2, 16.7, 16.6; HRMS (TOF MS ES+) m/z calcd for C_33_H_42_NO_9_Si^+^ [M+NH_4_]^+^: 624.2629; Found: 624.2637.

**TBDPSO-L_2_G_2_-COOBn**

**Appearance:** colorless oil**, Yield:** 86%**, Characterization:** R*_f_* = 0.25 (30% Et_2_O/hexane); IR (film)/cm^-1^ 3070, 2931, 2857, 1750 (C=O), 1451, 1170, 971, 702; ^1^H NMR (400 MHz, CDCl_3_) δ 7.72 – 7.66 (m, 4H), 7.50 – 7.32 (m, 11H), 5.20 (s, 2H), 5.01 (q, *J* = 7.1 Hz, 1H), 4.85 (d, *J* = 16.1 Hz, 1H), 4.79 – 4.63 (m, 3H), 4.35 (q, *J* = 6.7 Hz, 1H), 1.45 – 1.39 (m, 6H), 1.10 (s, 9H); ^13^C NMR (101 MHz, CDCl_3_) δ 173.1, 169.8, 166.8, 166.6, 136.0, 135.7, 134.8, 133.4, 133.0, 129.8, 128.7, 128.6, 128.4, 127.6, 127.6, 68.5, 68.3, 67.3, 61.1, 60.5, 26.8, 21.1, 19.2, 16.6; HRMS (TOF MS ES+) m/z calcd for C_33_H_38_O_9_NaSi^+^ [M+Na]^+^: 629.2183; Found: 629.2194.

**TBDPSO-G_4_-COOBn**

**Appearance:** colorless oil**, Yield:** 81%**, Characterization:** R*_f_* = 0.25 (30% EtOAc/hexane); IR (film)/cm^-1^ 3069, 2932, 2857, 1759 (C=O), 1588, 1424, 1394, 1270, 1165, 1136, 822, 703; ^1^H NMR (400 MHz, CDCl_3_) δ 7.72 – 7.67 (m, 4H), 7.48 – 7.35 (m, 11H), 5.21 (s, 2H), 4.81 (s, 2H), 4.75 (s, 2H), 4.74 (s, 2H), 4.37 (s, 2H), 1.10 (s, 9H); ^13^C NMR (101 MHz, CDCl_3_) δ 170.5, 166.8, 166.8, 166.5, 135.5, 134.8, 132.6, 129.9, 128.6, 128.6, 128.4, 127.8, 67.3, 61.8, 61.2, 60.7, 60.2, 26.6, 19.2; HRMS (TOF MS ES+) m/z calcd for C_31_H_34_O_9_NaSi^+^ [M+Na]^+^: 601.1870; Found: 601.1862.

**TBDPSO-L_4_-COOBn**

**Appearance:** colorless oil**, Yield:** 63%**, Characterization:** R*_f_* = 0.32 (15% EtOAc/hexane); IR (film)/cm^-1^ 3069, 290, 2931, 2856, 1751 (C=O), 1452, 1184, 1128, 1093, 702; ^1^H NMR (400 MHz, CDCl_3_) δ 7.74 – 7.65 (m, 4H), 7.46 – 7.31 (m, 11H), 5.23 – 5.10 (m, 4H), 4.95 (q, *J* = 7.1 Hz, 1H), 4.33 (q, *J* = 6.7 Hz, 1H), 1.52 (d, *J* = 7.1 Hz, 3H), 1.51 (d, *J* = 7.1 Hz, 3H), 1.42 (d, *J* = 6.7 Hz, 3H), 1.40 (d, *J* = 7.1 Hz, 3H), 1.10 (s, 9H); ^13^C NMR (101 MHz, CDCl_3_) δ 173.1, 169.9, 169.7, 135.9, 135.7, 135.1, 133.4, 133.0, 129.8, 128.6, 128.5, 128.2, 127.6, 127.6, 69.2, 68.8, 68.5, 68.2, 67.2, 26.8, 21.1, 19.2, 16.7, 16.6; HRMS (TOF MS ES+) m/z calcd for C_35_H_42_O_9_NaSi^+^ [M+Na]^+^: 657.2496; Found: 657.2501.

**TBDPSO-(LG)_3_-COOBn**

**Appearance:** colorless oil**, Yield:** 68%**, Characterization:** R*_f_* = 0.49 (30% EtOAc/hexane); IR (film)/cm^-1^ 3069, 2931, 2857, 1758 (C=O), 1588, 1452, 1424, 1389, 1175, 1131, 1101, 703; ^1^H NMR (400 MHz, CDCl_3_) δ 7.71 – 7.64 (m, 4H), 7.49 – 7.31 (m, 11H), 5.29 – 5.17 (m, 4H), 4.86 (d, *J* = 16.1 Hz, 1H), 4.82 (d, *J* = 15.9 Hz, 1H), 4.68 (d, *J* = 16.0 Hz, 1H), 4.66 – 4.60 (m, 2H), 4.45 (d, *J* = 16.0 Hz, 1H), 4.38 (q, *J* = 6.7 Hz, 1H), 1.56 (d, *J* = 7.1 Hz, 3H), 1.56 (d, *J* = 7.1 Hz, 3H), 1.43 (d, *J* = 6.7 Hz, 3H), 1.10 (s, 9H); ^13^C NMR (101 MHz, CDCl_3_) δ 173.0, 169.5, 169.4, 166.9, 166.8, 166.4, 135.9, 135.7, 134.8, 133.4, 132.9, 129.8, 128.6, 128.5, 127.6, 127.6, 69.3, 69.2, 68.9, 68.6, 67.3, 61.1, 60.8, 60.2, 26.8, 21.2, 19.2, 16.7, 16.7; HRMS (TOF MS ES+) m/z calcd for C_38_H_44_O_13_NaSi^+^ [M+Na]^+^: 759.2449; Found: 759.2456.

**TBDPSO-G_3_L_3_-COOBn**

**Appearance:** colorless oil**, Yield:** 70%**, Characterization:** R*_f_* = 0.42 (30% EtOAc/hexane); IR (film)/cm^-1^ 3069, 2934, 2857, 1754 (C=O), 1452, 1425, 1387, 1269, 1184, 1133, 1094, 703; ^1^H NMR (400 MHz, CDCl_3_) δ 7.72 – 7.67 (m, 4H), 7.48 – 7.31 (m, 11H), 5.25 – 5.11 (m, 5H), 4.82 (d, *J* = 16.1 Hz, 1H), 4.79 – 4.68 (m, 3H), 4.37 (s, 2H), 1.60 (d, *J* = 7.1 Hz, 3H), 1.54 (d, *J* = 7.1 Hz, 3H), 1.53 (d, *J* = 7.1 Hz, 3H), 1.09 (s, 9H); ^13^C NMR (101 MHz, CDCl_3_) δ 170.5, 169.9, 169.5, 169.4, 166.8, 166.5, 135.6, 132.6, 129.9, 128.6, 128.5, 128.2, 127.8, 69.3, 69.1, 67.2, 61.8, 60.8, 60.3, 26.6, 19.2, 16.7, 16.7, 16.6.

**TBDPSO-(LG)_4_-COOBn**

**Appearance:** colorless oil**, Yield:** 81%**, Characterization:** R*_f_* = 0.57 (35% EtOAc/hexane); IR (film)/cm^-1^ 3070, 2927, 2856, 1753 (C=O), 1588, 1451, 1423, 1387, 1275, 1171, 1128, 1091, 740, 701; ^1^H NMR (400 MHz, CDCl_3_) δ 7.72 – 7.64 (m, 4H), 7.48 – 7.32 (m, 11H), 5.31 – 5.18 (m, 5H), 4.92 – 4.79 (m, 3H), 4.73 – 4.60 (m, 4H), 4.46 (d, *J* = 16.0 Hz, 1H), 4.38 (q, *J* = 6.7 Hz, 1H), 1.59 (d, *J* = 7.1 Hz, 3H), 1.57 (d, *J* = 7.1 Hz, 3H), 1.56 (d, *J* = 7.1 Hz, 3H), 1.43 (d, *J* = 6.7 Hz, 3H), 1.10 (s, 9H); ^13^C NMR (101 MHz, CDCl_3_) δ 173.0, 169.5, 169.4, 169.4, 166.9, 166.8, 166.5, 166.4, 135.9, 135.7, 134.8, 133.4, 132.9, 129.8, 128.6, 128.5, 127.6, 127.6, 69.2, 69.1, 68.9, 68.6, 67.3, 61.1, 60.8, 60.7, 60.2, 26.8, 21.3, 19.2, 16.7; HRMS (TOF MS ES+) m/z calcd for C_43_H_50_O_17_NaSi^+^ [M+Na]^+^: 889.2715; Found: 889.2730.

**TBDPSO-L_4_G_4_-COOBn**

**Appearance:** colorless oil**, Yield:** 45 %**, Characterization:** R*_f_* = 0.43 (35% EtOAc/hexane); IR (film)/cm^-1^ 3069. 2938, 2857, 1752 (C=O), 1451, 1424, 1391, 1158, 1128, 1089, 740, 703; ^1^H NMR (400 MHz, CDCl_3_) δ 7.72 – 7.65 (m, 4H), 7.47 – 7.33 (m, 11H), 5.26 – 5.19 (m, 3H), 5.15 (q, *J* = 7.1 Hz, 1H), 4.95 (q, *J* = 7.1 Hz, 1H), 4.92 – 4.64 (m, 8H), 4.33 (q, *J* = 6.7 Hz, 1H), 1.59 (d, *J* = 8.2 Hz, 3H), 1.57 (d, *J* = 8.1 Hz, 3H), 1.42 (d, *J* = 6.8 Hz, 3H), 1.40 (d, *J* = 7.1 Hz, 3H), 1.10 (s, 9H); ^13^C NMR (101 MHz, CDCl_3_) δ 173.1, 170.0, 169.7, 169.5, 166.8, 166.4, 166.4, 166.3, 135.9, 135.7, 134.8, 133.4, 133.0, 129.8, 128.7, 128.4, 127.6, 127.6, 68.9, 68.8, 68.4, 68.2, 67.4, 61.2, 60.8, 60.7, 60.6, 26.8, 21.1, 19.2, 16.7, 16.6, 16.6; HRMS (TOF MS ES+) m/z calcd for C_43_H_50_O_17_NaSi^+^ [M+Na]^+^: 889.2715; Found: 889.2733.

**HO-GL-COOBn**

**Appearance:** colorless oil**, Yield:** 93%**, Characterization:** R*_f_* = 0.17 (60% Et_2_O/hexane); IR (film)/cm^-1^ 3473 (br, OH), 2944, 1744 (C=O), 1453, 1267, 1192, 1095, 745; ^1^H NMR (400 MHz, CDCl_3_) δ 7.43 – 7.32 (m, 5H), 5.26 (q, *J* = 7.1 Hz, 1H), 5.23 – 5.16 (m, 2H), 4.30 (d, *J* = 17.4 Hz, 1H), 4.24 (d, *J* = 17.4 Hz, 1H), 2.32 (br, 1H), 1.55 (d, *J* = 7.1 Hz, 3H); ^13^C NMR (101 MHz, CDCl_3_) δ 172.7, 170.0, 135.0, 128.6, 128.5, 128.1, 69.4, 67.2, 60.5, 16.8.

**HO-LG-COOBn**

**Appearance:** colorless oil**, Yield:** 93%**, Characterization:** R*_f_* = 0.16 (30% EtOAc/hexane); IR (film)/cm^-1^ 3488 (br, OH), 3034, 2985, 2944, 1744 (C=O), 1453, 1278, 1181, 1125, 752, 700; ^1^H NMR (400 MHz, CDCl_3_) δ 7.44 – 7.31 (m, 5H), 5.21 (s, 2H), 4.80 (d, *J* = 15.9 Hz, 1H), 4.71 (d, *J* = 15.9 Hz, 1H), 4.42 (q, *J* = 6.9 Hz, 1H), 2.16 (br, 1H), 1.48 (d, *J* = 7.0 Hz, 3H); ^13^C NMR (101 MHz, CDCl_3_) δ 175.1, 167.1, 134.8, 128.7, 128.5, 67.3, 66.7, 61.2, 20.3.

**HO-GG-COOBn**

**Appearance:** colorless oil**, Yield:** 90%**, Characterization:** R*_f_* = 0.20 (40% EtOAc/hexane); IR (film)/cm^-1^ 3309 (br, OH), 2952, 1744 (C=O), 1580, 1431, 1401, 1200, 1092, 1039, 700; ^1^H NMR (400 MHz, CDCl_3_) δ 7.45 – 7.31 (m, 5H), 5.22 (s, 2H), 4.78 (s, 2H), 4.31 (s, 2H), 2.33 (br, 1H); ^13^C NMR (101 MHz, CDCl_3_) δ 172.7, 167.1, 134.8, 128.7, 128.4, 67.4, 61.1, 60.4; HRMS (APCI) m/z calcd for C_11_H_11_O_5_^-^ [M-H]^-^: 223.0612; Found: 223.0611.

**HO-LL-COOBn**

**Appearance:** colorless oil**, Yield:** 64%**, Characterization:** R*_f_* = 0.12 (30% EtOAc/hexane); IR (film)/cm^-1^ 3490 (br, OH), 2987, 2939, 1737 (C=O), 1498, 1452, 1266, 1188, 1122, 1092, 1042, 743, 697; ^1^H NMR (400 MHz, CDCl_3_) δ 7.44 – 7.31 (m, 5H), 5.31 – 5.13 (m, 3H), 4.35 (q, *J* = 7.0 Hz, 1H), 2.55 (br, 1H), 1.55 (d, *J* = 7.1 Hz, 3H), 1.45 (d, *J* = 6.9 Hz, 3H); ^13^C NMR (101 MHz, CDCl_3_) δ 175.1, 170.0, 135.0, 128.6, 128.5, 128.2, 69.4, 67.2, 66.7, 20.4, 16.8; HRMS (TOF MS ES+) m/z calcd for C_13_H_16_O_5_Na^+^ [M+Na]^+^: 275.0895; Found: 275.0900.

**HO-LLL-COOBn**

**Appearance:** colorless oil**, Yield:** 88%**, Characterization:** R*_f_* = 0.20 (30% EtOAc/hexane); IR (film)/cm^-1^ 3490 (br, OH), 2917, 2849, 1737 (C=O), 1495, 1462, 1239, 1091, 740; ^1^H NMR (400 MHz, CDCl_3_) δ 7.43 – 7.31 (m, 5H), 5.25 – 5.12 (m, 4H), 4.36 (q, *J* = 6.9 Hz, 1H), 2.65 (s, 1H), 1.55 (d, *J* = 7.1 Hz, 3H), 1.55 (d, *J* = 7.1 Hz, 3H), 1.50 (d, *J* = 6.9 Hz, 3H); ^13^C NMR (101 MHz, CDCl_3_) δ 175.1, 169.9, 169.6, 128.6, 128.5, 128.3, 69.3, 69.1, 67.2, 66.7, 20.5, 16.8, 16.7; HRMS (TOF MS ES+) m/z calcd for C_16_H_21_O_7_^+^ [M+H]^+^: 325.1287; Found: 325.1286.

**HO-(LG)_2_-COOBn**

**Appearance:** colorless oil**, Yield:** 89%**, Characterization:** R*_f_* = 0.25 (30% EtOAc/hexane); IR (film)/cm^-1^ 3514 (br, OH), 2926. 2851, 1751 (C=O), 1587, 1349, 1278. 1177, 1133, 1099, 752, 700; ^1^H NMR (400 MHz, CDCl_3_) δ 7.43 – 7.32 (m, 5H), 5.28 (q, *J* = 7.1 Hz, 1H), 5.20 (s, 2H), 4.87 (d, *J* = 16.0 Hz, 1H), 4.82 (d, *J* = 15.9 Hz, 1H), 4.72 (d, *J* = 16.1 Hz, 1H), 4.64 (d, *J* = 15.9 Hz, 1H), 4.42 (q, *J* = 6.9 Hz, 1H), 2.35 (br, 1H), 1.58 (d, *J* = 7.1 Hz, 3H), 1.50 (d, *J* = 6.9 Hz, 3H); ^13^C NMR (101 MHz, CDCl_3_) δ 174.9, 169.4, 166.9, 166.6, 134.8, 128.7, 128.5, 69.2, 67.3, 66.7, 61.1, 60.8, 20.3, 16.7; HRMS (TOF MS ES+) m/z calcd for C_17_H_20_O_9_Na^+^ [M+Na]^+^: 391.1005; Found: 391.1006.

**HO-G_4_-COOBn**

**Appearance:** colorless oil**, Yield:** 60%**, Characterization:** R*_f_* = 0.28 (55% EtOAc/hexane); IR (film)/cm^-1^ 3500 (br, OH), 2953, 1743 (C=O), 1422, 1394, 1273, 1154, 1095, 1074, 737, 697; ^1^H NMR (400 MHz, CDCl_3_) δ 7.43 – 7.32 (m, 5H), 5.21 (s, 2H), 4.86 (s, 2H), 4.84 (s, 2H), 4.75 (s, 2H), 4.32 (s, 2H), 2.18 (br, 1H); ^13^C NMR (101 MHz, CDCl_3_) δ 172.6, 166.8, 166.6, 166.5, 134.8, 128.7, 128.4, 67.4, 61.2, 60.8, 60.7, 60.4; HRMS (TOF MS ES+) m/z calcd for C_15_H_16_O_9_Na^+^ [M+Na]^+^: 363.0692; Found: 363.0701.

**TBDPSO-GL-COOH**

**Appearance:** colorless oil**, Yield:** 100%**, Characterization:** IR (film)/cm^-1^ broad OH vibration, 3049, 2937, 2862, 1745 (C=O), 1729 (C=O), 1431, 1192, 1140, 820, 700; ^1^H NMR (400 MHz, CDCl_3_) δ 7.73 – 7.67 (m, 4H), 7.46 – 7.37 (m, 6H), 5.15 (q, *J* = 7.1 Hz, 1H), 4.38 (d, *J* = 16.9 Hz, 1H), 4.33 (d, *J* = 16.9 Hz, 1H), 1.51 (d, *J* = 7.1 Hz, 3H), 1.10 (s, 9H); ^13^C NMR (101 MHz, CDCl_3_) δ 176.1, 170.6, 135.6, 135.5, 132.6, 132.6, 129.9, 127.8, 68.2, 61.9, 26.6, 19.2, 16.7; HRMS (APCI) m/z calcd for C_21_H_25_O_5_Si^-^ [M-H]^-^: 385.1477; Found: 385.1465.

**TBDPSO-LG-COOH**

**Appearance:** colorless oil**, Yield:** 95%**, Characterization:** IR (film)/cm^-1^ broad OH vibration visible, 3069, 2932, 2857, 1728 (C=O), 1588, 1424, 1132, 1105, 820, 738, 698, 609, 504, 483; ^1^H NMR (400 MHz, CDCl_3_) δ 7.73 – 7.64 (m, 4H), 7.49 – 7.34 (m, 6H), 4.60 (d, *J* = 16.4 Hz, 1H), 4.50 (d, *J* = 16.4 Hz, 1H), 4.40 (q, *J* = 6.8 Hz, 1H), 1.43 (d, *J* = 6.8 Hz, 3H), 1.10 (s, 9H); ^13^C NMR (101 MHz, CDCl_3_) δ 173.0, 172.4, 135.9, 135.7, 133.4, 132.9, 129.8, 127.7, 127.6, 68.6, 60.0, 26.8, 21.2, 19.2; HRMS (TOF MS ES+) m/z calcd for C_21_H_26_O_5_NaSi^+^ [M+Na]^+^: 409.1447; Found: 409.1461.

**TBDPSO-GG-COOH**

**Appearance:** colorless oil**, Yield:** 100%**, Characterization:** IR (film)/cm^-1^ broad OH vibration visible, 3071, 2929, 2855, 1763 (C=O), 1729 (C=O), 1580, 1431, 1259, 1200, 1110, 931, 805, 700; ^1^H NMR (400 MHz, CDCl_3_) δ 7.75 – 7.66 (m, 4H), 7.47 – 7.37 (m, 6H), 4.69 (s, 2H), 4.38 (s, 2H), 1.10 (s, 9H); ^13^C NMR (101 MHz, CDCl_3_) δ 171.8, 170.6, 135.5, 134.8, 132.6, 130.0, 127.8, 61.8, 60.0, 26.6, 19.2; HRMS (TOF MS ES+) m/z calcd for C_20_H_24_O_5_NaSi^+^ [M+Na]^+^: 395.1291; Found: 395.1291.

**TBDPSO-LL-COOH**

**Appearance:** colorless oil**, Yield:** 100%**, Characterization:** IR (film)/cm^-1^broad OH vibration observed, 3071, 2933, 2858, 1763 (C=O), 1722 (C=O), 1587, 1461, 1427, 1185, 1107, 972, 909, 820, 734, 700; ^1^H NMR (400 MHz, CDCl_3_) δ 7.72 – 7.66 (m, 4H), 7.47 – 7.34 (m, 6H), 4.95 (q, *J* = 7.1 Hz, 1H), 4.34 (q, *J* = 6.7 Hz, 1H), 1.42 (d, *J* = 6.7 Hz, 3H), 1.39 (d, *J* = 7.2 Hz, 3H), 1.10 (s, 9H); ^13^C NMR (101 MHz, CDCl_3_) δ 176.1, 173.1, 136.0, 135.7, 133.4, 133.0, 129.8, 127.6, 127.6, 68.5, 68.1, 26.8, 21.1, 19.2, 16.6; HRMS (TOF MS ES+) m/z calcd for C_22_H_28_O_5_NaSi^+^ [M+Na]^+^: 423.1604; Found: 423.1598.

**TBDPSO-LL-COOH**

**Appearance:** colorless oil**, Yield:** 100%**, Characterization:** IR (film)/cm^-1^ broad OH vibration observed, 3071, 2933, 2858, 1763 (C=O), 1722 (C=O), 1587, 1461, 1427, 1185, 1107, 972, 909, 820, 734, 700; ^1^H NMR (400 MHz, CDCl_3_) δ 7.72 – 7.66 (m, 4H), 7.47 – 7.34 (m, 6H), 4.95 (q, *J* = 7.1 Hz, 1H), 4.34 (q, *J* = 6.7 Hz, 1H), 1.42 (d, *J* = 6.7 Hz, 3H), 1.39 (d, *J* = 7.2 Hz, 3H), 1.10 (s, 9H); ^13^C NMR (101 MHz, CDCl_3_) δ 176.1, 173.1, 136.0, 135.7, 133.4, 133.0, 129.8, 127.6, 127.6, 68.5, 68.1, 26.8, 21.1, 19.2, 16.6; HRMS (TOF MS ES+) m/z calcd for C_22_H_28_O_5_NaSi^+^ [M+Na]^+^: 423.1604; Found: 423.1598.

**TBDPSO-GGG-COOH**

**Appearance:** colorless oil**, Yield:** 98%**, Characterization:** IR (film)/cm^-1^ broad OH vibration observed, 2926, 2849, 1721(C=O), 1423, 1102, 1082, 809; ^1^H NMR (400 MHz, CDCl_3_) δ 7.72 – 7.66 (m, 4H), 7.49 – 7.35 (m, 6H), 4.75 (s, 2H), 4.74 (s, 2H), 4.38 (s, 2H), 1.10 (s, 9H); ^13^C NMR (101 MHz, CDCl_3_) δ 171.0, 170.6, 166.8, 135.6, 132.6, 130.0, 127.8, 61.8, 60.4, 60.3, 26.6, 19.2; HRMS (TOF MS ES+) m/z calcd for C_22_H_26_O_7_NaSi^+^ [M+Na]^+^: 453.1346; Found: 453.1351.

**TBDPSO-(GL)_2_-COOH**

**Appearance:** yellowish oil**, Yield:** 99%**, Characterization:** IR (film)/cm^-1^ broad OH vibration observed, 3069, 2932, 1744 (C=O), 1590, 1185, 1130, 740, 702 ; ^1^H NMR (400 MHz, CDCl_3_) δ 7.76 – 7.66 (m, 4H), 7.47 – 7.37 (m, 6H), 5.25 – 5.13 (m, 2H), 4.88 (d, *J* = 16.1 Hz, 1H), 4.64 (d, *J* = 16.1 Hz, 1H), 4.38 (d, *J* = 16.8, 1H), 4.32 (d, *J* = 16.9 Hz, 1H), 1.57 (d, *J* = 7.2 Hz, 3H), 1.53 (d, *J* = 7.1 Hz, 3H), 1.09 (s, 9H); ^13^C NMR (101 MHz, CDCl_3_) δ 175.2, 170.7, 169.9, 166.6, 135.6, 135.5, 132.6, 129.9, 127.8, 68.9, 68.4, 61.9, 60.7, 26.6, 19.2, 16.8, 16.6; HRMS (APCI) m/z calcd for C_26_H_31_O_9_Si^-^ [M-H]^-^: 515.1743, Found: 515.1730.

**TBDPSO-L_4_-OH**

**Appearance:** colorless oil**, Yield:** 100%**, Characterization:** IR (film)/cm^-1^ 3069, 2991, 2932, 2857, 1755 (C=O), 1588, 1452, 1376, 1187, 1129, 095, 703; ^1^H NMR (400 MHz, CDCl_3_) δ 7.73 – 7.65 (m, 4H), 7.49 – 7.34 (m, 6H), 5.16 (q, *J* = 7.1 Hz, 2H), 4.95 (q, *J* = 7.1 Hz, 1H), 4.34 (q, *J* = 6.7 Hz, 1H), 1.56 (d, *J* = 7.1 Hz, 3H), 1.56 (d, *J* = 7.1 Hz, 3H), 1.42 (d, *J* = 6.8 Hz, 3H), 1.40 (d, *J* = 7.2 Hz, 3H), 1.10 (s, 9H); ^13^C NMR (101 MHz, CDCl_3_) δ 174.9, 173.2, 170.0, 169.6, 135.9, 135.7, 133.4, 133.0, 129.8, 127.6, 127.6, 68.8, 68.7, 68.5, 68.3, 26.8, 21.1, 19.2, 16.6, 16.6; HRMS (TOF MS ES+) m/z calcd for C_28_H_36_O_9_NaSi^+^ [M+Na]^+^: 567.2026; Found: 567.2033.

**TBDPSO-(LG)_2_-COOH**

**Appearance:** yellowish oil**, Yield:** 97%**, Characterization:** IR (film)/cm^-1^ broad OH vibration, 3071, 2929, 2858, 1763 (C=O), 1427, 1185, 1133, 1110, 704; ^1^H NMR (400 MHz, CDCl_3_) δ 7.71 – 7.63 (m, 4H), 7.50 – 7.34 (m, 6H), 5.24 (q, *J* = 7.1 Hz, 1H), 4.80 (d, *J* = 16.4 Hz, 1H), 4.73 – 4.61 (m, 2H), 4.46 (d, *J* = 16.0 Hz, 1H), 4.39 (q, *J* = 6.8 Hz, 1H), 1.56 (d, *J* = 7.1 Hz, 3H), 1.43 (d, *J* = 6.8 Hz, 3H), 1.10 (s, 9H); ^13^C NMR (101 MHz, CDCl_3_) δ 173.1, 171.0, 169.4, 166.9, 135.9, 135.7, 133.4, 132.9, 129.8, 127.7, 127.6, 69.0, 68.6, 60.5, 60.3, 26.8, 21.3, 19.2, 16.7; HRMS (TOF MS ES+) m/z calcd for C_26_H_32_O_9_NaSi [M+Na]^+^: 539.1713, Found: 539.1709.

**TBDPSO-G_2_L_2_-COOH**

**Appearance:** yellowish oil**, Yield:** 94%**, Characterization:** IR (film)/cm^-1^ broad OH vibration observed, 3070, 2932, 2857, 1744 (C=O), 1589, 1426, 1186, 1131, 1102, 821, 741, 703; ^1^H NMR (400 MHz, CDCl_3_) δ 7.75 – 7.64 (m, 4H), 7.49 – 7.35 (m, 6H), 5.28 – 5.14 (m, 2H), 4.77 (d, *J* = 16.1 Hz, 1H), 4.67 (d, *J* = 16.1 Hz, 1H), 4.37 (d, *J* = 16.7 Hz, 1H), 4.37 (d, *J* = 16.7 Hz, 1H), 1.57 (d, *J* = 7.1 Hz, 6H), 1.09 (s, 9H); ^13^C NMR (101 MHz, CDCl_3_) δ 175.2, 170.6, 169.4, 166.9, 135.5, 132.6, 129.9, 127.8, 69.1, 68.7, 61.8, 60.4, 26.6, 19.2, 16.6; HRMS (TOF MS ES+) m/z calcd for C_26_H_36_NO_9_Si^+^ [M+NH_4_]^+^: 534.2159, Found: 534.2155.

**TBDPSO-L_2_G_2_-COOH**

**Appearance:** yellowish oil**, Yield:** 100%**, Characterization:** IR (film)/cm^-1^ broad OH vibration, 3329, 2931, 2855, 1760, 1587, 1425, 1176, 1131, 108, 972, 704, 507; ^1^H NMR (400 MHz, CDCl_3_) δ 7.74 – 7.62 (m, 4H), 7.49 – 7.32 (m, 6H), 5.01 (q, *J* = 7.1 Hz, 1H), 4.84 (d, *J* = 16.2 Hz, 1H), 4.80 – 4.63 (m, 3H), 4.35 (q, *J* = 6.7 Hz, 1H), 1.44 – 1.40 (m, 6H), 1.10 (s, 9H); ^13^C NMR (101 MHz, CDCl_3_) δ 173.2, 171.4, 169.9, 166.6, 136.0, 135.7, 133.4, 133.0, 129.8, 127.6, 127.6, 68.5, 68.3, 60.5, 26.8, 21.1, 19.2, 16.6; HRMS (APCI) m/z calcd for C_26_H_31_O_9_Si^-^ [M-H]^-^: 515.1743, Found: 515.1732.

**TBDPSO-(LG)_3_-COOH**

**Appearance:** yellowish oil**, Yield:** 90%**, Characterization:** IR (film)/cm^-1^ broad OH vibration visible, 3069, 2930, 2855, 1750 (C=O), 1589, 1454, 1384, 1130, 702; ^1^H NMR (400 MHz, CDCl_3_) δ 7.71 – 7.65 (m, 4H), 7.49 – 7.33 (m, 6H), 5.31 – 5.20 (m, 2H), 4.87 (d, *J* = 16.1 Hz, 1H), 4.81 (d, *J* = 16.3 Hz, 1H), 4.72 – 4.61 (m, 3H), 4.46 (d, *J* = 16.0 Hz, 1H), 4.39 (q, *J* = 6.7 Hz, 1H), 1.59 (d, *J* = 7.1 Hz, 3H), 1.57 (d, *J* = 7.0 Hz, 3H), 1.43 (d, *J* = 6.8 Hz, 3H), 1.10 (s, 9H); ^13^C NMR (101 MHz, CDCl_3_) δ 173.1, 171.5, 169.5, 169.3, 166.9, 166.5, 135.9, 135.7, 133.3, 132.9, 129.8, 127.7, 127.6, 69.2, 68.9, 68.6, 60.8, 60.6, 60.3, 26.8, 21.2, 19.2, 16.7, 16.7; HRMS (TOF MS ES+) m/z calcd for C_31_H_42_NO_13_Si^+^ [M+NH_4_]^+^: 664.2425, Found: 664.2437.

**TBDPSO-G_3_L_3_-COOH**

**Appearance:** yellowish oil**, Yield:** 100%**, Characterization:** IR (film)/cm^-1^ broad OH vibration observable, 3070, 2928, 2856, 1747 (C=O), 1589, 1454, 1426, 1385, 1191, 1132, 1108, 821, 704; ^1^H NMR (400 MHz, CDCl_3_) δ 7.73 – 7.65 (m, 4H), 7.48 – 7.36 (m, 6H), 5.26 – 5.14 (m, 3H), 4.81 (d, *J* = 16.1 Hz, 1H), 4.77 – 4.67 (m, 3H), 4.37 (s, 2H), 1.60 (d, *J* = 7.1 Hz, 3H), 1.59 (d, *J* = 7.1 Hz, 3H), 1.57 (d, *J* = 7.2 Hz, 3H), 1.09 (s, 9H); ^13^C NMR (101 MHz, CDCl_3_) δ 174.4, 170.6, 169.5, 169.5, 166.8, 166.5, 135.5, 132.6, 129.9, 127.8, 69.3, 69.1, 68.7, 61.8, 60.8, 60.3, 26.6, 19.2, 16.6, 16.6; HRMS (TOF MS ES+) m/z calcd for C_38_H_44_O_13_NaSi^+^ [M+Na]^+^: 759.2449, Found: 759.2455.

**TBDPSO-(LG)_4_-COOH**

**Appearance:** yellowish oil**, Yield:** 99%**, Characterization:** IR (film)/cm^-1^ broad OH vibration visible, 2926, 2855, 1751 (C=O), 1588, 1452, 1423, 1275, 1171, 1128, 1092, 741, 702; ^1^H NMR (400 MHz, CDCl_3_) δ 7.70 – 7.65 (m, 4H), 7.48 – 7.34 (m, 6H), 5.32 – 5.19 (m, 3H), 4.91 – 4.79 (m, 3H), 4.72 – 4.61 (m, 4H), 4.46 (d, *J* = 16.0 Hz, 1H), 4.38 (q, *J* = 6.7 Hz, 1H), 1.59 (d, *J* = 7.1 Hz, 6H), 1.56 (d, *J* = 7.1 Hz, 3H), 1.42 (d, *J* = 6.7 Hz, 3H), 1.10 (s, 9H); ^13^C NMR (101 MHz, CDCl_3_) δ 173.1, 171.1, 169.5, 169.4, 169.3, 166.9, 166.5, 166.5, 135.9, 135.7, 133.4, 132.9, 129.8, 127.7, 127.6, 69.2, 69.1, 69.0, 68.6, 60.8, 60.8, 60.5, 60.3, 26.8, 21.3, 19.2, 16.7, 16.7; HRMS (TOF MS ES+) m/z calcd for C_36_H_44_O_17_NaSi^+^ [M+Na]^+^: 799.2245; Found: 799.2250.

**TBDPSO-L_4_G_4_-COOH**

**Appearance:** yellowish oil**, Yield:** 96%**, Characterization:** IR (film)/cm^-1^ 3071, 2953, 2857, 1758 (C=O), 1425, 1165, 1131, 1095, 705; ^1^H NMR (400 MHz, CDCl_3_) δ 7.76 – 7.62 (m, 4H), 7.48 – 7.31 (m, 6H), 5.22 (q, *J* = 7.1 Hz, 1H), 5.16 (q, *J* = 7.1 Hz, 1H), 4.95 (q, *J* = 7.1 Hz, 1H), 4.91 – 4.81 (m, 5H), 4.79 – 4.68 (m, 3H), 4.33 (q, *J* = 6.7 Hz, 1H), 1.59 (d, *J* = 7.2 Hz, 3H), 1.57 (d, *J* = 7.2 Hz, 4H), 1.42 (d, *J* = 6.8 Hz, 3H), 1.39 (d, *J* = 7.1 Hz, 3H), 1.09 (s, 9H); ^13^C NMR (101 MHz, CDCl_3_) δ 173.3, 170.6, 170.0, 169.7, 169.5, 166.5, 166.4, 166.4, 135.9, 135.7, 133.4, 133.0, 129.8, 127.6, 127.6, 68.9, 68.8, 68.5, 68.3, 60.8, 60.7, 60.6, 26.8, 21.1, 19.2, 16.6, 16.6, 16.5; HRMS (TOF MS ES+) m/z calcd for C_36_H_44_O_17_NaSi^+^ [M+Na]^+^: 799.2245; Found: 799.2256.

**TBDPSO-(GL)_2_-ethyl methacrylate**

**Appearance:** colorless oil**, Yield:** 68%**, Characterization:** R*_f_* = 0.20 (20% EtOAc/hexane); IR (film)/cm^-1^ 3080, 2952, 2862, 1759 (C=O), 1720 (C=O), 1640, 1453, 1274, 1095, 946, 820, 700; ^1^H NMR (400 MHz, CDCl_3_) δ 7.74 – 7.65 (m, 4H), 7.48 – 7.35 (m, 6H), 6.16 – 6.09 (m, 1H), 5.63 – 5.58 (m, 1H), 5.23 – 5.13 (m, 2H), 4.87 (d, *J* = 16.0 Hz, 1H), 4.63 (d, *J* = 16.0 Hz, 1H), 4.51 – 4.27 (m, 6H), 1.98 – 1.91 (m, 3H), 1.53 (d, *J* = 7.1, 3H), 1.52 (d, *J* = 7.1 Hz, 3H), 1.09 (s, 9H); 13C NMR (101 MHz, CDCl_3_) δ 170.6, 169.8, 169.8, 167.0, 166.6, 135.7, 135.6, 135.5, 132.6, 129.9, 127.8, 126.2, 69.3, 68.4, 63.1, 61.9, 61.9, 60.7, 26.6, 19.2, 18.2, 16.8, 16.7.

**TBDPSO-(LG)_2_-ethyl methacrylate**

**Appearance:** colorless oil**, Yield:** 89%**, Characterization:** R*_f_* = 0.25 (30% EtOAc/hexane); IR (film)/cm^-1^ 3048, 2931, 2857, 1728 (C=O), 1588, 1468, 1426, 1136, 1109, 820, 700; ^1^H NMR (400 MHz, CDCl_3_) δ 7.71 – 7.64 (m, 4H), 7.48 – 7.34 (m, 6H), 6.15 – 6.11 (m, 1H), 5.61 (quint, *J* = 1.6 Hz, 1H), 5.24 (q, *J* = 7.1 Hz, 1H), 4.79 (d, *J* = 15.9 Hz, 1H), 4.68 (d, *J* = 16.0 Hz, 1H), 4.61 (d, *J* = 16.0 Hz, 1H), 4.50 – 4.33 (m, 6H), 1.97 – 1.93 (m, 3H), 1.56 (d, *J* = 7.1 Hz, 3H), 1.43 (d, *J* = 6.8 Hz, 3H), 1.10 (s, 9H); ^13^C NMR (101 MHz, CDCl_3_) δ 173.0, 169.5, 167.0, 166.9, 166.8, 135.9, 135.7, 133.4, 132.9, 129.8, 127.7, 127.6, 126.3, 69.0, 68.6, 63.1, 62.0, 60.9, 60.2, 26.8, 21.3, 19.2, 18.2, 16.7; HRMS (TOF MS ES+) m/z calcd for C_32_H_40_O_11_NaSi^+^ [M+Na]^+^: 651.2238, Found: 651.2240.

**TBDPSO-G_2_L_2_-ethyl methacrylate**

**Appearance:** colorless oil**, Yield:** 70%**, Characterization:** R*_f_* = 0.30 (25% EtOAc/hexane); IR (film)/cm^-1^ 3071, 2933, 2855, 1751(C=O), 1720 (C=O), 1636, 1453, 1129, 1095, 823, 741, 700, 611; ^1^H NMR (400 MHz, CDCl_3_) δ 7.75 – 7.63 (m, 4H), 7.51 – 7.33 (m, 6H), 6.16 – 6.09 (m, 1H), 5.61 (quint, *J* = 1.5 Hz, 1H), 5.31 – 5.11 (m, 2H), 4.77 (d, *J* = 16.1 Hz, 1H), 4.66 (d, *J* = 16.1 Hz, 1H), 4.53 – 4.29 (m, 6H), 1.97 – 1.93 (m, 3H), 1.57 (d, *J* = 7.1 Hz, 3H), 1.54 (d, *J* = 7.1 Hz, 3H), 1.09 (s, 9H); ^13^C NMR (101 MHz, CDCl_3_) δ 170.5, 169.9, 169.4, 167.0, 166.9, 135.7, 135.5, 132.6, 129.9, 127.8, 126.3, 69.1, 69.0, 63.0, 62.0, 61.8, 60.3, 26.6, 19.2, 18.2, 16.7, 16.6.

**TBDPSO-L_2_G_2_-ethyl methacrylate**

**Appearance:** colorless oil**, Yield:** 72%**, Characterization:** R*_f_* = 0.33 (50% Et_2_O/hexane); IR (film)/cm^-1^ 3070, 2934, 2956, 2858, 1759 (C=O), 1722 (C=O), 1636, 1425, 1159, 1130. 1107, 972, 704, 508; ^1^H NMR (400 MHz, CDCl_3_) δ 7.73 – 7.64 (m, 4H), 7.49 – 7.33 (m, 6H), 6.16 – 6.09 (m, 1H), 5.61 (quint, *J* = 1.6 Hz, 1H), 5.01 (q, *J* = 7.1 Hz, 1H), 4.84 (d, *J* = 16.1 Hz, 1H), 4.77 – 4.64 (m, 3H), 4.46 – 4.40 (m, 2H), 4.39 – 4.31 (m, 3H), 1.97 – 1.93 (m, 3H), 1.46 – 1.39 (m, 6H), 1.10 (s, 9H); ^13^C NMR (101 MHz, CDCl_3_) δ 173.0, 169.8, 167.0, 166.8, 166.6, 135.9, 135.7, 133.4, 133.0, 129.8, 127.6, 127.6, 126.3, 68.5, 68.2, 63.2, 61.9, 60.9, 60.5, 26.8, 21.1, 19.2, 18.2, 16.6; HRMS (TOF MS ES+) m/z calcd for C_32_H_41_O_11_Si^+^ [M+H]^+^: 629.2418, Found: 629.2429.

**TBDPSO-(LG)_3_-ethyl methacrylate**

**Appearance:** colorless oil**, Yield:** 58%**, Characterization:** R*_f_* = 0.55 (70% Et_2_O/hexane); IR (film)/cm^-1^ 3071, 2992, 2955, 2858, 1761, 1722, 1636, 1425, 1173, 1132, 1102, 705, 509; ^1^H NMR (400 MHz, CDCl_3_) δ 7.73 – 7.63 (m, 4H), 7.51 – 7.33 (m, 6H), 6.13 (quint, *J* = 1.1 Hz, 1H), 5.62 (quint, *J* = 1.6 Hz, 1H), 5.31 – 5.19 (m, 2H), 4.87 (d, *J* = 16.0 Hz, 1H), 4.80 (d, *J* = 16.0 Hz, 1H), 4.72 – 4.58 (m, 3H), 4.49 – 4.33 (m, 6H), 1.97 – 1.92 (m, 3H), 1.59 (d, *J* = 7.1 Hz, 3H), 1.57 (d, *J* = 7.1 Hz, 3H), 1.43 (d, *J* = 6.7 Hz, 3H), 1.10 (s, 9H); ^13^C NMR (101 MHz, CDCl_3_) δ 173.0, 169.5, 169.4, 167.0, 166.8, 166.5, 135.9, 135.7, 133.4, 132.9, 129.8, 127.7, 127.6, 126.3, 69.2, 68.9, 68.6, 63.2, 62.0, 60.9, 60.7, 60.2, 26.8, 21.3, 19.2, 18.2, 16.7, 16.7; HRMS (TOF MS ES+) m/z calcd for C_37_H_50_NO_15_Si^+^ [M+HH_4_]^+^: 776.2950, Found: 776.2955.

**TBDPSO-G_3_L_3_-ethyl methacrylate**

**Appearance:** colorless oil**, Yield:** 73%**, Characterization:** R*_f_* = 0.24 (35% EtOAc/hexane); IR (film)/cm^-1^ 3069, 2952, 2857, 1755 (C=O), 1721 (C=O), 1451, 1425, 1382, 1169, 1133, 1094, 704; ^1^H NMR (400 MHz, CDCl_3_) δ 7.72 – 7.67 (m, 4H), 7.48 – 7.36 (m, 6H), 6.15 – 6.11 (m, 1H), 5.61 (quint, *J* = 1.6 Hz, 1H), 5.26 – 5.12 (m, 3H), 4.86 – 4.70 (m, 4H), 4.49 – 4.34 (m, 6H), 1.98 – 1.93 (m, 3H), 1.60 (d, *J* = 7.1, 3H), 1.59 (d, *J* = 7.1 Hz, 3H), 1.53 (d, *J* = 7.1 Hz, 3H), 1.09 (s, 9H); 13C NMR (101 MHz, CDCl3) δ 170.5, 169.9, 169.5, 169.4, 167.0, 166.8, 166.5, 135.5, 132.6, 129.9, 127.8, 126.2, 69.2, 69.1, 69.1, 63.0, 62.0, 61.8, 60.8, 60.2, 26.6, 19.2, 18.2, 16.7, 16.6, 16.6; HRMS (TOF MS ES+) m/z calcd for C_37_H_46_O_15_NaSi^+^ [M+Na]^+^: 781.2504, Found: 781.2492.

**TBDPSO-(LG)_4_-ethyl methacrylate**

**Appearance:** colorless oil**, Yield:** 69%**, Characterization:** R*_f_* = 0.62 (8% MeCN/CH_2_Cl_2_); IR (film)/cm^-1^ 3080, 2992, 2954, 2857, 1753 (C=O), 1720 (C=O), 1635, 1423, 1277, 1169, 1128, 1093, 964, 742, 704, 612, 508; ^1^H NMR (400 MHz, CDCl_3_) δ 7.71 – 7.64 (m, 4H), 7.47 – 7.34 (m, 6H), 6.16 – 6.11 (m, 1H), 5.62 (quint, *J* = 1.6 Hz, 1H), 5.32 – 5.18 (m, 3H), 4.92 – 4.58 (m, 7H), 4.49 – 4.34 (m, 6H), 1.95 (dd, *J* = 1.6, 1.0 Hz, 3H), 1.59 (d, *J* = 7.1 Hz, 6H), 1.56 (d, *J* = 7.1 Hz, 3H), 1.42 (d, *J* = 6.8 Hz, 3H), 1.10 (s, 9H); ^13^C NMR (101 MHz, CDCl_3_) δ 173.0, 169.5, 169.4, 167.0, 166.8, 166.5, 166.4, 135.9, 135.7, 133.4, 132.9, 129.8, 127.6, 127.6, 126.3, 69.2, 69.1, 68.9, 68.6, 63.1, 62.0, 60.9, 60.8, 60.7, 60.2, 26.8, 21.3, 19.2, 18.2, 16.7; HRMS (TOF MS ES+) m/z calcd for C_42_H_52_O_19_NaSi^+^ [M+Na]+: 911.2770, Found: 911.2783.

**TBDPSO-L_4_G_4_-ethyl methacrylate**

**Appearance:** colorless oil**, Yield:** 75%**, Characterization:** R*_f_* = 0.60 (8% MeCN/CH_2_Cl_2_); IR (film)/cm^-1^ 3071, 2928, 2857, 1757 (C=O), 1722 (C=O), 1636, 1451, 1425, 1384, 1158, 1130, 1093, 705; ^1^H NMR (400 MHz, CDCl_3_) δ 7.72 – 7.65 (m, 4H), 7.49 – 7.33 (m, 6H), 6.16 – 6.12 (m, 1H), 5.62 (quint, *J* = 1.6 Hz, 1H), 5.22 (q, *J* = 7.1 Hz, 1H), 5.16 (q, *J* = 7.1 Hz, 1H), 4.95 (q, *J* = 7.1 Hz, 1H), 4.89 (d, *J* = 16.1 Hz, 1H), 4.85 – 4.73 (m, 6H), 4.69 (d, *J* = 16.1 Hz, 1H), 4.48 – 4.40 (m, 2H), 4.40 – 4.30 (m, 3H), 1.95 (dd, *J* = 1.6, 1.0 Hz, 3H), 1.59 (d, *J* = 7.1 Hz, 3H), 1.57 (d, *J* = 7.1 Hz, 3H), 1.42 (d, *J* = 6.7 Hz, 3H), 1.40 (d, J = 7.1 Hz, 3H), 1.09 (s, 9H); ^13^C NMR (101 MHz, CDCl_3_) δ 173.1, 169.9, 169.7, 169.5, 167.0, 166.7, 166.4, 166.4, 166.3, 135.9, 135.7, 133.4, 133.0, 129.8, 127.6, 127.5, 126.3, 68.9, 68.8, 68.4, 68.2, 63.2, 61.9, 61.0, 60.8, 60.7, 60.6, 26.8, 21.1, 19.2, 18.2, 16.7, 16.6, 16.5; HRMS (TOF MS ES+) m/z calcd for C_42_H_52_O_19_NaSi^+^ [M+Na]^+^: 911.2770, Found: 911.2791.

**HO-(GL)_2_-ethyl methacrylate**

**Appearance:** colorless oil**, Yield:** 86%**, Characterization:** R*_f_* = 0.24 (50% EtOAc/hexane); IR (film)/cm^-1^ 3503 (br. OH), 2959, 1751 (C=O), 1720 (C=O), 1640, 1453, 1177, 1095; ^1^H NMR (400 MHz, CDCl_3_) δ 6.15 – 6.11 (m, 1H), 5.63 – 5.59 (m, 1H), 5.30 (q, *J* = 7.1 Hz, 1H), 5.19 (q, *J* = 7.1 Hz, 1H), 4.87 (d, *J* = 16.0 Hz, 1H), 4.67 (d, *J* = 16.0 Hz, 1H), 4.52 – 4.42 (m, 1H), 4.42 – 4.22 (m, 5H), 2.56 – 2.17 (br, 1H), 1.98 – 1.92 (m, 3H), 1.61 (d, J = 7.1 Hz, 3H), 1.53 (d, J = 7.1 Hz, 3H); ^13^C NMR (101 MHz, CDCl_3_) δ 172.6, 169.7, 169.6, 167.0, 166.4, 135.8, 126.2, 69.4, 69.0, 63.1, 62.0, 60.8, 60.5, 18.2, 16.8, 16.8; HRMS (TOF MS ES+) m/z calcd for C_16_H_23_O_11_^+^ [M+H]^+^: 391.1240; Found: 391.1228.

**HO-(LG)_2_-ethyl methacrylate**

**Appearance:** colorless oil, **Yield:** 76%, **Characterization:** R*_f_* = 0.37 (60% EtOAc/hexane); IR (film)/cm^-1^ 3500 (br. OH), 2986, 2957, 1750 (C=O), 1719 (C=O), 1635, 1451, 1384, 1292, 1169, 1128, 1096, 950; ^1^H NMR (400 MHz, CDCl_3_) δ 6.16 – 6.09 (m, 1H), 5.60 (quint, *J* = 1.6 Hz, 1H), 5.26 (q, *J* = 7.1 Hz, 1H), 4.86 (d, *J* = 16.0 Hz, 1H), 4.79 (d, *J* = 15.9 Hz, 1H), 4.72 (d, *J* = 16.0 Hz, 1H), 4.62 (d, *J* = 16.0 Hz, 1H), 4.46 – 4.33 (m, 5H), 3.34 – 2.44 (br, 1H), 1.94 (t, *J* = 1.2 Hz, 3H), 1.59 (d, *J* = 7.1 Hz, 3H), 1.49 (d, *J* = 7.0 Hz, 3H); ^13^C NMR (101 MHz, CDCl_3_) δ 174.8, 169.4, 167.0, 166.8, 166.6, 135.7, 126.3, 69.1, 66.7, 63.1, 62.0, 60.9, 60.8, 20.3, 18.2, 16.7; HRMS (TOF MS ES+) m/z calcd for C_16_H_22_O_11_Na^+^ [M+Na]^+^: 413.1060, Found: 413.1066.

**HO-G_2_L_2_-ethyl methacrylate**

**Appearance:** colorless oil**, Yield:** 84%**, Characterization:** R*_f_* = 0.20 (50% EtOac/hexane); IR (film)/cm^-1^ 3503 (br, OH), 2952, 1744 (C=O), 1720 (C=O), 1640, 1453, 1386, 1162, 1088, 954; ^1^H NMR (400 MHz, CDCl_3_) δ 6.18 – 6.08 (m, 1H), 5.61 (quint, *J* = 1.6 Hz, 1H), 5.28 – 5.14 (m, 2H), 4.86 (d, *J* = 16.1 Hz, 1H), 4.78 (d, *J* = 16.0 Hz, 1H), 4.50 – 4.29 (m, 6H), 2.22 (s, 1H), 1.95 (dd, *J* = 1.6, 1.0 Hz, 3H), 1.60 (d, *J* = 7.1 Hz, 3H), 1.54 (d, *J* = 7.1 Hz, 3H); ^13^C NMR (101 MHz, CDCl_3_) δ 172.6, 169.8, 169.3, 167.0, 166.6, 135.8, 126.3, 69.3, 69.2, 63.0, 62.0, 60.8, 60.4, 18.2, 16.7, 16.7.

**HO-L_2_G_2_-ethyl methacrylate**

**Appearance:** colorless oil**, Yield:** 60%**, Characterization:** R*_f_* = 0.28 (70% Et_2_O/hexane); IR (film)/cm^-1^ 3500 (br, OH), 2987, 2958, 1753, 1720, 1635, 1388, 1295, 1166, 1129, 1097, 943; ^1^H NMR (400 MHz, CDCl_3_) δ 6.16 – 6.10 (m, 1H), 5.61 (quint, *J* = 1.6 Hz, 1H), 5.32 – 5.23 (m, 1H), 4.89 (d, *J* = 16.1 Hz, 1H), 4.79 – 4.68 (m, 3H), 4.50 – 4.29 (m, 5H), 2.87 – 2.70 (m, 1H), 1.95 (t, *J* = 1.3 Hz, 3H), 1.61 (d, *J* = 7.1 Hz, 3H), 1.50 (d, *J* = 6.9 Hz, 3H); ^13^C NMR (101 MHz, CDCl_3_) δ 175.0, 169.5, 167.0, 166.8, 166.5, 135.7, 126.3, 69.0, 66.7, 63.2, 61.9, 61.0, 60.7, 20.4, 18.2, 16.7; HRMS (TOF MS ES+) m/z calcd for C_16_H_23_O_11_^+^ [M+H]^+^: 391.1240, Found: 391.1226.

**HO-(LG)_3_-ethyl methacrylate**

**Appearance:** colorless oil**, Yield:** 82%**, Characterization:** R*_f_* = 0.22 (50% EtOAc/hexane); IR (film)/cm^-1^ 3531 (br, OH), 2993, 2957, 1755 (C=O), 1720 (C=O), 1635, 1386, 1279, 1176, 1130, 1096, 955; ^1^H NMR (400 MHz, CDCl_3_) δ 6.17 – 6.10 (m, 1H), 5.63 – 5.56 (m, 1H), 5.33 – 5.22 (m, 2H), 4.94 – 4.57 (m, 6H), 4.47 – 4.32 (m, 5H), 2.75 (d, *J* = 5.3 Hz, 1H), 1.95 (s, 3H), 1.60 (d, *J* = 7.2 Hz, 3H), 1.59 (d, J = 7.0 Hz, 3H), 1.50 (d, *J* = 7.0 Hz, 3H); ^13^C NMR (101 MHz, CDCl_3_) δ 174.9, 169.4, 167.0, 166.8, 166.6, 166.4, 135.7, 126.3, 69.2, 69.1, 66.7, 63.2, 62.0, 60.9, 60.8, 60.8, 20.3, 18.2, 16.7, 16.7; HRMS (ES+) m/z calcd for C_21_H_28_O_15_Na^+^  [M+Na]^+^: 543.1326, Found: 543.1334.

**HO-G_3_L_3_-ethyl methacrylate**

**Appearance:** colorless oil**, Yield:** 75%**, Characterization:** R*_f_* = 0.24 (50% EtOAc/hexane); IR (film)/cm^-1^ 3520 (br, OH), 2993, 2952, 1750 (C=O), 1720 (C=O), 1633, 1386, 1176, 954; ^1^H NMR (400 MHz, CDCl_3_) δ 6.16 – 6.10 (m, 1H), 5.61 (quint, *J* = 1.6 Hz, 1H), 5.28 – 5.13 (m, 3H), 4.91 – 4.72 (m, 4H), 4.50 – 4.25 (m, 6H), 1.95 (t, *J* = 1.3 Hz, 3H), 1.60 (d, *J* = 7.1, 3H), 1.59 (d, *J* = 7.1 Hz, 3H), 1.53 (d, *J* = 7.1 Hz, 3H); ^13^C NMR (101 MHz, CDCl_3_) δ 172.6, 169.9, 169.5, 169.4, 167.0, 166.6, 166.4, 135.8, 126.2, 69.3, 69.1, 69.1, 63.0, 62.0, 60.9, 60.7, 60.4, 18.2, 16.7, 16.6, 16.6.

**HO-(LG)_4_-ethyl methacrylate**

**Appearance:** colorless oil**, Yield:** 76%**, Characterization:** R*_f_* = 0.27 (60% EtOAc/hexane); IR (film)/cm^-1^ 3501 (br, OH), 2993, 2953, 2925, 1756 (C=O), 1720 (C=O), 1635, 1385, 1175, 1130, 1095, 956; ^1^H NMR (400 MHz, CDCl_3_) δ 6.16 – 6.05 (m, 1H), 5.65 – 5.54 (m, 1H), 5.30 – 5.22 (m, 3H), 4.92 – 4.59 (m, 8H), 4.45 – 4.33 (m, 5H), 2.64 – 2.37 (br, 1H), 1.97 – 1.90 (m, 3H), 1.62 – 1.57 (m, 9H), 1.49 (d, *J* = 7.0 Hz, 3H); ^13^C NMR (101 MHz, CDCl_3_) δ 174.9, 169.4, 167.0, 166.8, 166.6, 166.4, 135.7, 126.3, 69.1, 69.1, 66.7, 63.1, 62.0, 60.9, 60.8, 20.3, 18.2, 16.7, 16.7; HRMS (TOF MS ES+) m/z calcd for C_26_H_34_O_19_Na^+^ [M+Na]^+^: 673.1592, Found: 673.1584.

**HO-L_4_G_4_-ethyl methacrylate**

**Appearance:** colorless oil**, Yield:** 82%**, Characterization:** R*_f_* = 0.18 (55% EtOAc/hexane); IR (film)/cm^-1^ 3460 (br, OH), 1745 (C=O), 1720 (C=O), 1634, 1167, 720 ; ^1^H NMR (400 MHz, CDCl_3_) δ 6.13 (quint, *J* = 1.0 Hz, 1H), 5.62 (quint, *J* = 1.6 Hz, 1H), 5.31 – 5.13 (m, 3H), 4.97 – 4.66 (m, 8H), 4.46 – 4.42 (m, 2H), 4.40 – 4.33 (m, 3H), 1.95 (dd, *J* = 1.6, 1.0 Hz, 3H), 1.64 – 1.57 (m, 9H), 1.50 (d, *J* = 6.9 Hz, 3H); ^13^C NMR (101 MHz, CDCl_3_) δ 169.9, 169.5, 169.4, 167.3, 167.0, 166.6, 166.5, 135.8, 135.2, 127.2, 126.3, 69.3, 69.2, 69.1, 63.1, 62.1, 60.9, 60.7, 60.5, 18.2, 18.2, 16.7, 16.7, 16.6; HRMS (TOF MS ES+) m/z calcd for C_26_H_34_O_19_Na^+^ [M+Na]^+^: 673.1592, Found: 673.1577.

**4Aa**

**Appearance:** colorless oil, **Yield**: 95%, **Characterization:** R*_f_* = 0.3 (30% EtOAc/hexane); IR (film)/cm^-1^ 2990, 2959, 1752 (C=O), 1722 (C=O), 1640, 1453, 1148, 909, 730; ^1^H NMR (400 MHz, CDCl_3_) δ 6.24 – 6.21 (m, 1H), 6.13 – 6.12 (m, 1H), 5.66 (quint, J = 1.6 Hz, 1H), 5.61 (quint, J = 1.6 Hz, 1H), 5.27 (q, J = 7.1 Hz, 1H), 5.18 (q, J = 7.0 Hz, 1H), 4.91 – 4.79 (m, 2H), 4.74 (d, J = 16.2 Hz, 1H), 4.64 (d, J = 16.0 Hz, 1H), 4.50 – 4.41 (m, 1H), 4.40 – 4.33 (m, 3H), 2.00 – 1.96 (m, 3H), 1.96 – 1.93 (m, 3H), 1.62 – 1.57 (m, 3H), 1.52 (d, J = 7.1 Hz, 3H); ^13^C NMR (101 MHz, CDCl_3_) δ 169.7, 169.5, 167.2, 167.0, 166.5, 166.5, 135.8, 135.2, 127.0, 126.2, 69.3, 68.9, 63.1, 61.9, 60.8, 60.6, 18.2, 18.2, 16.7; HRMS (ES+) m/z calcd for C_20_H_26_O_12_Na^+^  [M+Na]^+^: 481.1316, Found: 481.1327.

**4Ab**

**Appearance:** colorless oil, **Yield**: 68%, **Characterization:** R*_f_* = 0.19 (30% EtOAc/hexane); IR (film)/cm^-1^ 2991, 2957, 1753 (C=O), 1717 (C=O), 1635, 1451, 1381, 1288, 1155, 1090, 943, 813; ^1^H NMR (400 MHz, CDCl_3_) δ 6.23 – 6.20 (m, 1H), 6.14 – 6.12 (m, 1H), 5.65 (quint, *J* = 1.5 Hz, 1H), 5.61 (quint, *J* = 1.5 Hz, 1H), 5.30 – 5.18 (m, 2H), 4.90 (d, *J* = 16.1 Hz, 1H), 4.80 (d, *J* = 16.0 Hz, 1H), 4.65 (d, *J* = 16.0 Hz, 1H), 4.62 (d, *J* = 16.0 Hz, 1H), 4.45 – 4.41 (m, 2H), 4.38 – 4.35 (m, 2H), 1.98 – 1.96 (m, 3H), 1.96 – 1.93 (m, 3H) , 1.62 (d, *J* = 7.1 Hz, 3H), 1.59 (d, *J* = 7.1 Hz, 3H); ^13^C NMR (101 MHz, CDCl_3_) δ 170.25, 169.42, 166.99, 166.85, 166.61, 135.75, 135.42, 126.70, 126.27, 69.10, 68.49, 63.14, 61.98, 60.93, 60.66, 18.21, 18.12, 16.86, 16.73; HRMS (TOF MS ES+) m/z calcd for C_20_H_26_O_12_Na^+^ [M+Na]+: 481.1322, Found: 481.1308.

**4Ba**

**Appearance:** colorless oil, **Yield:** 80%, **Characterization:** R*_f_* = 0.24 (30% EtOAc/hexane); IR (film)/cm^-1^  2991, 2952, 1752 (C=O), 1722 (C=O), 1640, 1453, 1274, 1140, 946, 812; ^1^H NMR (400 MHz, CDCl_3_) δ 6.24 – 6.22 (m, 1H), 6.14 – 6.11 (m, 1H), 5.67 (quint, *J* = 1.6 Hz, 1H), 5.61 (quint, *J* = 1.6 Hz, 1H), 5.27 – 5.12 (m, 2H), 4.88 – 4.71 (m, 4H), 4.50 – 4.30 (m, 4H), 1.98 (dd, *J* = 1.6, 1.0 Hz, 3H), 1.94 (dd, *J* = 1.6, 1.0 Hz, 3H), 1.58 (d, *J* = 7.1 Hz, 3H), 1.53 (d, *J* = 7.1 Hz, 3H); ^13^C NMR (101 MHz, CDCl_3_) δ 169.8, 169.3, 167.2, 166.9, 166.5, 166.5, 135.7, 135.2, 127.1, 126.2, 69.2, 69.2, 63.0, 62.0, 60.7, 60.5, 18.2, 18.2, 16.7, 16.6; HRMS (ES+) m/z calcd for C_20_H_26_O_12_Na^+^ [M+Na]^+^: 481.1316, Found: 481.1326.

**4Bb**

**Appearance:** colorless oil, **Yield:** 52%, **Characterization:** R*_f_* = 0.22 (25% EtOAc/hexane); IR (film)/cm^-1^ 2991, 2956, 1756 (C=O), 1719 (C=O), 1635, 1451, 1292, 1156, 1129, 1095, 946, 614; ^1^H NMR (400 MHz, CDCl_3_) δ 6.21 (s, 1H), 6.14 (s, 1H), 5.66 – 5.64 (m, 1H), 5.63 – 5.60 (m, 1H), 5.26 (q, *J* = 7.0 Hz, 1H), 5.17 (q, *J* = 7.1 Hz, 1H), 4.89 (d, *J* = 16.1 Hz, 1H), 4.78 – 4.66 (m, 3H), 4.46 – 4.40 (m, 2H), 4.40 – 4.35 (m, 2H), 1.99 – 1.96 (m, 3H), 1.96 – 1.94 (m, 3H), 1.62 (d, J = 7.0 Hz, 6H); ^13^C NMR (101 MHz, CDCl_3_) δ 170.2, 169.6, 167.0, 166.7, 166.7, 166.5, 135.7, 135.4, 126.6, 126.3, 68.7, 68.5, 63.2, 61.9, 60.9, 60.6, 18.2, 18.1, 16.7, 16.7; HRMS (TOF MS ES+) m/z calcd for C_20_H_27_O_12_^+^ [M+H]^+^: 459.1503, Found: 459.1519.

**6A**

**Appearance:** colorless oil, **Yield:** 51%, **Characterization:** R*_f_* = 0.21; IR (film)/cm^-1^ 2993, 2956, 1758 (C=O), 1721 (C=O), 1636, 1452, 1288, 1165, 1130, 1095, 951; ^1^H NMR (400 MHz, CDCl_3_) δ 6.22 (s, 1H), 6.13 (s, 1H), 5.67 – 5.60 (m, 2H), 5.33 – 5.18 (m, 3H), 4.93 – 4.77 (m, 3H), 4.71 – 4.58 (m, 3H), 4.46 – 4.40 (m, 2H), 4.39 – 4.35 (m, 2H), 1.98 – 1.96 (m, 3H), 1.96 – 1.94 (m, 3H), 1.64 – 1.58 (m, 9H); ^13^C NMR (101 MHz, CDCl_3_) δ 170.3, 169.4, 169.4, 167.0, 166.8, 166.6, 166.4, 135.7, 135.4, 126.7, 126.3, 69.2, 69.1, 68.5, 63.2, 62.0, 60.9, 60.8, 60.7, 18.2, 18.1, 16.9, 16.7; HRMS (TOF MS ES+) m/z calcd for C_25_H_33_O_16_^+^ [M+H]^+^: 589.1763, Found: 589.1785.

**6B**

**Appearance:** colorless oil, **Yield:** 55%, **Characterization:** R*_f_* = 0.20 (40% EtOAc/hexane); IR (film)/cm^-1^ 2922, 2855, 1751 (C=O), 1720 (C=O), 1640, 1453, 1379, 1140, 954, 812; ^1^H NMR (400 MHz, CDCl_3_) δ 6.24 (quint, *J* = 1.0 Hz, 1H), 6.16 – 6.11 (m, 1H), 5.68 (quint, *J* = 1.6 Hz, 1H), 5.61 (quint, *J* = 1.6 Hz, 1H), 5.26 – 5.12 (m, 3H), 4.88 – 4.73 (m, 6H), 4.49 – 4.40 (m, 1H), 4.39 – 4.32 (m, 3H), 1.99 (dd, J = 1.6, 1.0 Hz, 3H), 1.95 (dd, J = 1.6, 1.0 Hz, 3H), 1.60 (d, J = 7.1 Hz, 3H), 1.59 (d, *J* = 7.1 Hz, 3H), 1.53 (d, J = 7.1 Hz, 3H); ^13^C NMR (101 MHz, CDCl_3_) δ 169.9, 169.5, 169.4, 167.0, 166.5, 166.4, 135.8, 135.2, 127.1, 126.2, 69.3, 69.1, 69.1, 63.0, 62.0, 60.9, 60.6, 60.5, 18.2, 18.2, 16.7, 16.6, 16.6; HRMS (TOF MS ES+) m/z calcd for C_25_H_32_O_16_Na^+^ [M+Na]^+^: 611.1588, Found: 611.1575.

**8A**

**Appearance:** colorless oil, **Yield:** 51%, Characterization: R*_f_* = 0.43 (45% EtOAc/ hexane); IR (film)/cm^-1^ 2922, 2855, 1759 (C=O), 1722 (C=O), 1640, 1453, 1174, 1095, 954; ^1^H NMR (400 MHz, CDCl_3_) δ 6.25 – 6.17 (m, 1H), 6.16 – 6.09 (m, 1H), 5.66 – 5.63 (m, 1H), 5.63 – 5.60 (m, 1H), 5.30 – 5.16 (m, 4H), 4.93 – 4.85 (m, 3H), 4.80 (d, *J* = 15.9 Hz, 1H), 4.69 – 4.59 (m, 4H), 4.46 – 4.40 (m, 2H), 4.39 – 4.34 (m, 2H), 1.97 (t, *J* = 1.3 Hz, 3H), 1.95 (t, *J* = 1.3 Hz, 3H), 1.63 – 1.58 (m, 12H); ^13^C NMR (101 MHz, CDCl_3_) δ 170.2, 169.4, 169.4, 167.0, 166.8, 166.6, 166.6, 166.4, 166.4, 135.7, 135.4, 126.7, 126.3, 69.2, 69.1, 69.0, 68.5, 63.1, 62.0, 60.9, 60.8, 60.6, 18.2, 18.1, 16.8, 16.7; HRMS (TOF MS ES+) m/z calcd for C_30_H_42_NO_20_^+^ [M+NH_4_]^+^: 736.2300, Found: 736.2309.

**8B**

**Appearance:** colorless oil, **Yield:** 30%, **Characterization:** R*_f_* = 0.58 (60% EtOAc/hexane); IR (film)/cm^-1^ 2921, 2852, 1752 (C=O), 1718 (C=O), 1635, 1454, 1379, 1292, 1155, 1128, 1084, 947, 732; ^1^H NMR (400 MHz, CDCl_3_) δ 6.25 – 6.18 (m, 1H), 6.16 – 6.10 (m, 1H), 5.64 (quint, *J* = 1.5 Hz, 1H), 5.62 (quint, *J* = 1.6 Hz, 1H), 5.28 – 5.13 (m, 4H), 4.92 – 4.68 (m, 8H), 4.47 – 4.41 (m, 2H), 4.41 – 4.35 (m, 2H), 1.97 (t, *J* = 1.3 Hz, 3H), 1.95 (t, *J* = 1.3 Hz, 3H), 1.64 – 1.56 (m, 12H); ^13^C NMR (101 MHz, CDCl_3_) δ 170.4, 169.8, 169.6, 169.5, 166.7, 166.4, 166.4, 166.3, 135.8, 135.4, 126.6, 126.3, 68.9, 68.8, 68.5, 63.2, 61.9, 61.1, 60.8, 60.7, 60.6, 18.2, 18.1, 16.8, 16.7, 16.6, 16.6; HRMS (TOF MS ES+) m/z calcd for C_30_H_38_O_20_Na^+^ [M+Na]^+^: 741.1854, Found: 741.1853.

# S17. ^1^H and ^13^C NMR spectra of OLGADMAs

**4Aa**

^1^H NMR (400 MHz, CDCl_3_)

**4Aa**

^13^C NMR (101 MHz, CDCl_3_)

**4Ab**

^1^H NMR (400 MHz, CDCl_3_)

**4Ab**

^13^C NMR (101 MHz, CDCl_3_)

**
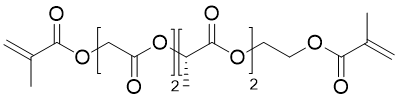
**

**4Ba**

^1^H NMR (400 MHz, CDCl_3_)

**4Ba**

^13^C NMR (101 MHz, CDCl_3_)

**
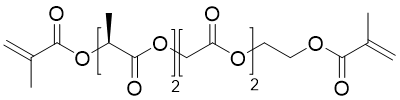
**

**4Bb**

^1^H NMR (400 MHz, CDCl_3_)

**4Bb**

^13^C NMR (101 MHz, CDCl_3_)

**
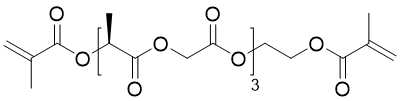
**

**6A**

^1^H NMR (400 MHz, CDCl_3_)

**6A**

^13^C NMR (101 MHz, CDCl_3_)

**
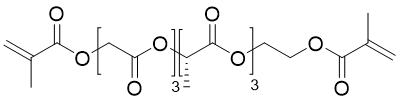
**

**6B**

^1^H NMR (400 MHz, CDCl_3_)

**6B**

^13^C NMR (101 MHz, CDCl_3_)

**
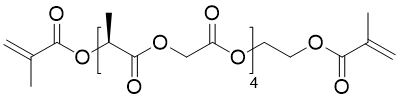
**

**8A**

^1^H NMR (400 MHz, CDCl_3_)

**8A**

^13^C NMR (101 MHz, CDCl_3_)

**8B**

^1^H NMR (400 MHz, CDCl_3_)

**8B**

^13^C NMR (101 MHz, CDCl_3_)

# S18. Optimization of dexamethasone encapsulation

The optimization of dexamethasone encapsulation was carried-out during nanoprecipitation polymerization of **4Aa**. The objective was to identify a reaction composition which would allow to obtain a stable NP suspension without the need for filtration or sonication, whilst maximizing the tolerated amount of dexamethasone present in the reaction mixture and the NP yield. Examples of optimization runs with a varying amount of dexamethasone in the absence and presence of PEGDA700 are listed in Table S2.

| PEGDA700 | Dexamethasone  (wt% of monomer feed) | NP Suspension Obtained | Estimated NP Yield** (%) | z-average (nm) | PI |
| --- | --- | --- | --- | --- | --- |
| - | 10 | Yes | 8 | 259.6 | 0.04 |
| - | 25 | Yes | 6 | 318.2 | 0.26 |
| - | 37.5 | - | - | - | - |
| - | 50 | - | - | - | - |
| - | 100 | - | - | - | - |
| yes | 37.5 | Yes | 40 | 279.1 | 0.06 |
| yes | 50 | Yes | 31 | 316.0 | 0.14 |
| yes | 100 | - | - | - | - |

**Table S3.** The outcome of nanoprecipitation polymerizations of **4Aa** with different concentrations of dexamethasone in the absence and presence of PEGDA700.

# S19. ATR-FTIR spectra of NPs

# S20. ^1^H NMR and DLS study of the nanoprecipitation polymerization reaction

Compounds **4Aa** and PEGDA700 were subjected to the nanoprecipitation polymerization under standard conditions. Samples (0.2 mL) of the reaction mixture were taken at determined time points, quickly diluted into ice cold deionized water (0.6 mL) with exposure to air and analyzed by DLS. The so-prepared suspensions were then freeze-dried for 48 h, dissolved in deuterated DMSO (0.75 mL) and analyzed by ^1^H NMR.

# S21. Stability of dexamethasone under typical reaction conditions

A solution of dexamethasone (6 mg) and AIBN (4 mg) in THF (1.6 mL) was quickly injected into water (4.8 mL) at 70 ºC, stirring at 1000 rpm, with the system open to air and under N_2_ gas flow. The mixture was immediately subjected to nitrogen gas bubbling for 5 minutes and then left under nitrogen gas atmosphere for a further 10 min. The mixture was then opened to air, cooled in an ice bath for 2 minutes and freeze-dried for 48 h. The lyophilizates were subjected to ^1^H NMR analysis as presented below.

**
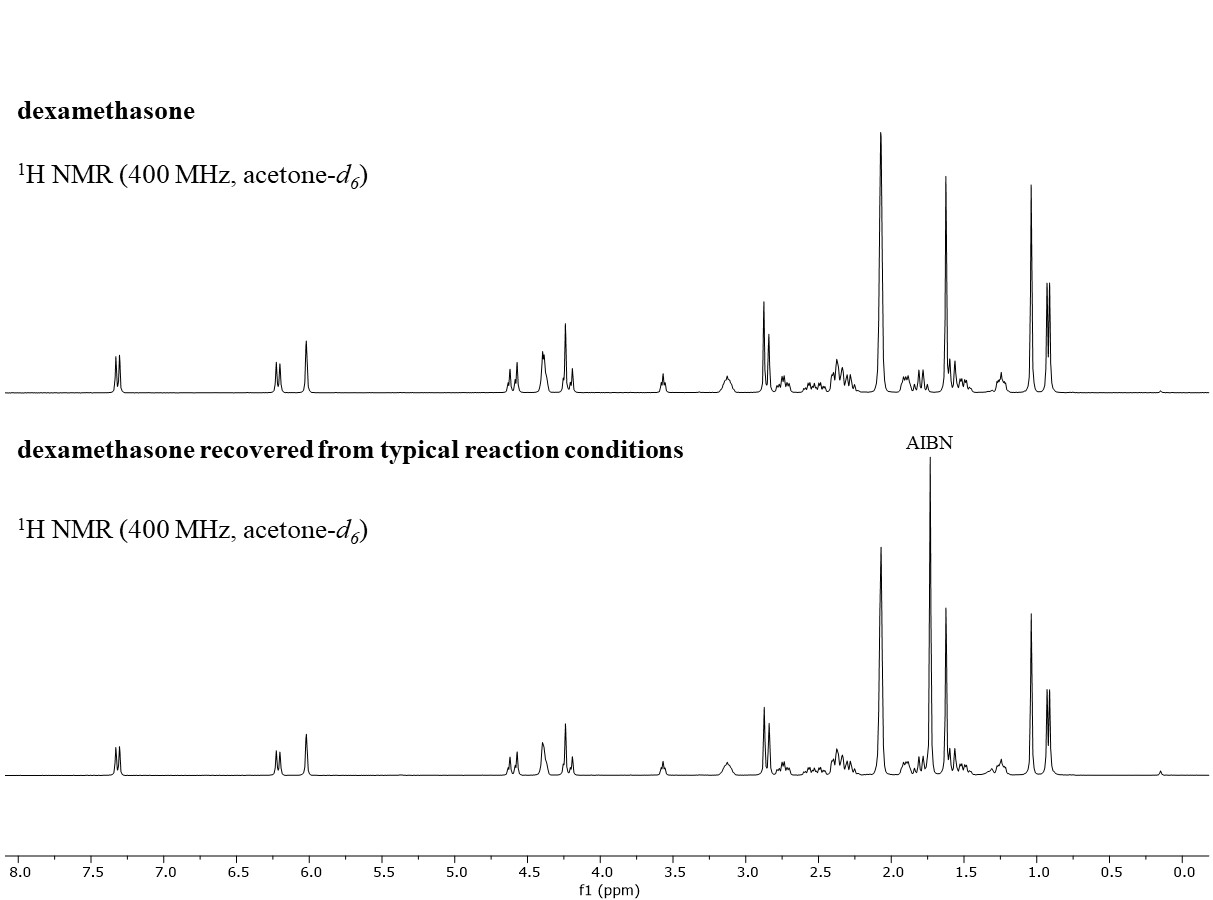
**

**Figure S1.** Comparison of ^1^H NMR spectrum (400 MHz, acetone-*d6*) of dexamethasone and the solid obtained from the procedure described above.

# S22. ^1^H NMR analysis of released dexamethasone

The combined filtrates deriving from the dexamethasone release assay of **DNP4Aa** were freeze-dried and subjected to ^1^H NMR analysis as presented in Figure S2.


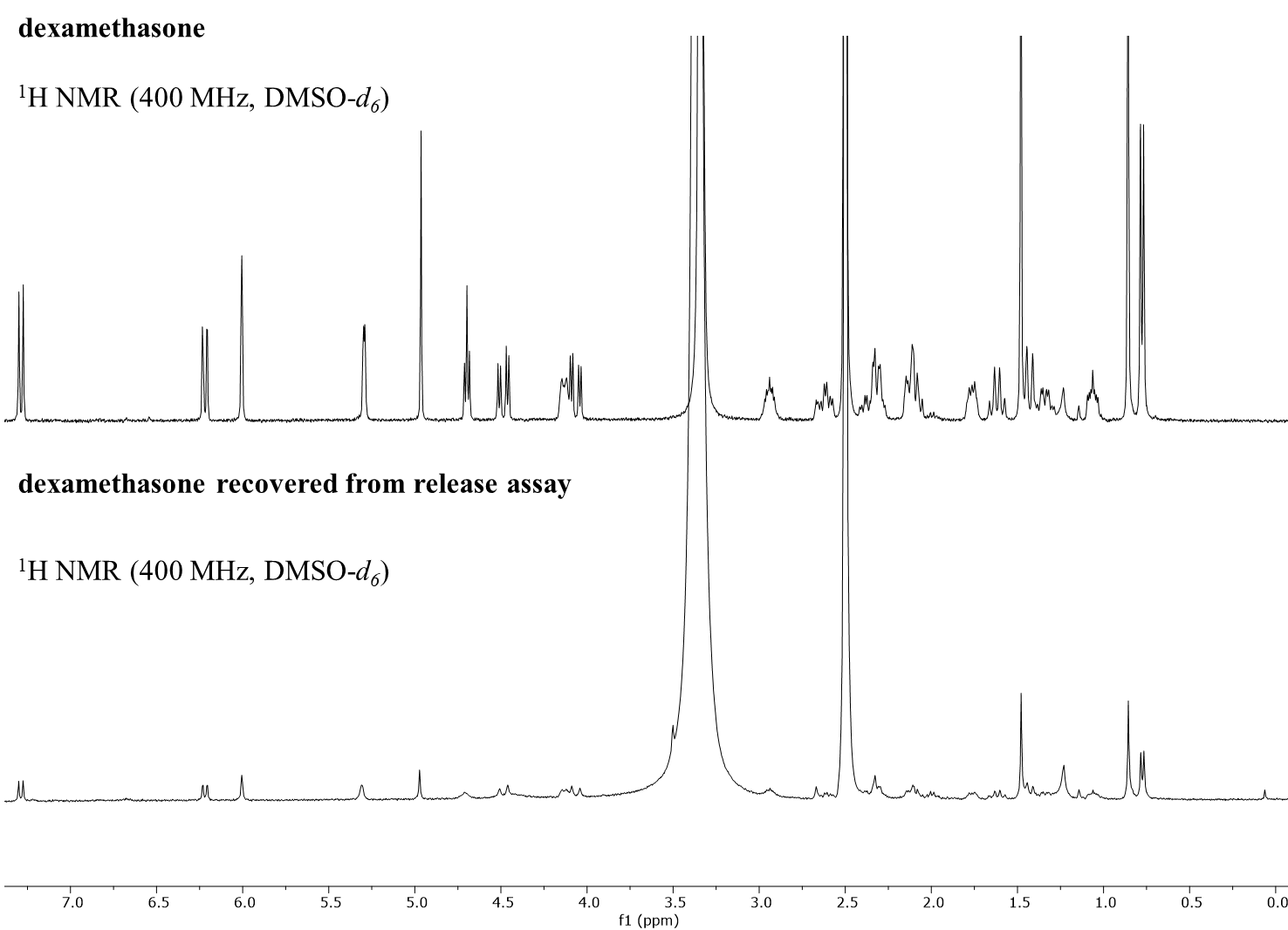


**Figure S2.** Comparison of ^1^H NMR spectrum (400 MHz, DMSO-*d6*) of dexamethasone and the freeze-dried solid obtained from the dexamethasone release assay of **DNP4Aa**.

# S23. Size-tunability of dexamethasone-loaded OLGADMA-based NPs

Fives sizes of dexamethasone-loaded OLGADMA-based NPs have been prepared from **4Aa** by variation of the duration of the nitrogen gas purging step described in the nanoprecipitation polymerization protocol. The quantitative reaction compositions were identical, the total reaction time was kept at 15 minutes, and the obtained reaction mixtures were subjected standard purification conditions. The analysis of the obtained NP suspensions is described in Table S3.

| N_2_ purge duration (min) | Z-average (nm) | PI | ζ-potential (mV) | | EE (%) |
| --- | --- | --- | --- | --- | --- |
| 0 | 313.1 ± 6.96 | 0.15 ± 0.02 | -48.4 ± 1.3 | 66.6 | |
| 1 | 303.1 ± 5.11 | 0.13 ± 0.02 | -32.9 ± 0.1 | 70.0 | |
| 5 | 279.1 ± 2.50 | 0.06 ± 0.05 | -37.0 ± 0.001 | 72.4 | |
| 10 | 178.4 ± 1.04 | 0.19 ± 0.06 | -45.0 ± 0.8 | 54.6 | |
| 15 | 104.9 ± 1.38 | 0.17 ± 0.04 | -37.9 ± 1.4 | 36.3 | |

**Table S2.** Analysis of dexamethasone-loaded OLGADMA-based NPs prepared from **4Aa** with variations of the duration of the N_2_ purge step of nanoprecipitation polymerization.

# S24. Synthesis of PLGA alt

PLGA alt was prepared according to procedures reported by Stayshich and Meyer, according to the scheme presented below.^2^

**Preparation:** The compound was prepared from **HO-GL-COOBn** following the general procedure for catalytic hydrogenation. **Appearance:** colourless oil, **Yield:** 97%, **Characterisation:** IR (film)/cm^-1^ 3445 (OH), broad OH (acid), 1729 (C=O), 1200, 1094; ^1^H NMR (400 MHz, CDCl_3_) δ 6.57 (br. 1H), 5.24 (q, *J* = 7.1 Hz, 1H), 4.32 (d, *J*  = 17.4 Hz, 1H), 4.26 (d, *J* = 17.4 Hz, 1H), 1.57 (d, *J* = 7.1 Hz, 3H); ^13^C NMR (101 MHz, CDCl_3_) δ 174.9, 172.8, 69.0, 60.4, 16.7; HRMS (ESI(-)-FTMS) m/z calcd for [M-H]^-^: 147.0299, Found: 147.0299. The spectroscopic data was consistent with that previously reported.^2^

**Preparation:** DMAP (7.3 mg, 0.06 mmol) was added to a solution of **HO-GL-COOH** (100.0 mg, 0.7 mmol) in EtOAc (0.2 mL) and CH_2_Cl_2_ (0.2 mL) at rt and the solution was cooled to 0 °C. Then, DCC (217.0 mg, 1.05 mmol) was added slowly over 2 min. The reaction mixture was then warmed to rt and allowed to stir for 24 h. The mixture was then diluted with CH_2_Cl_2_, filtered through a short plug of celite, and the resulting solution precipitated into cold methanol. The resulting suspension was filtered to afford the desired polymer. **Appearance:** white solid, **Characterisation:** IR (film)/cm^-1^ 2996, 2946, 1756 (C=O), 1175, 1094; ^1^H NMR (400 MHz, CDCl_3_) δ 5.26 (q, J = 7.0 Hz, 1H), 4.89 (d, J = 16.0 Hz, 1H), 4.66 (d, J = 16.0 Hz, 1H), 1.60 (d, J = 7.1 Hz, 3H); ^13^C NMR (101 MHz, CDCl_3_) δ 169.4, 166.4, 69.1, 60.8, 16.7; GPC (THF) *M*n: 5954, *M*w: 7158, PDI: 1.20. The spectroscopic data was consistent with that previously reported.^2^

# S25. Dynamic Light Scattering (DLS) and Electrophoretic Light Scattering (ELS)

Z-average, PI and ζ-potential were determined using a Malvern Zetasizer Ultra instrument. For size analysis, samples were prepared by mixing 40-50 µl of nanoparticle dispersions with the appropriate dispersant (1 mL). Measurements were performed using polystyrene cuvettes at 25 °C, measuring the scattered light at an angle of 173°. The samples were then transferred to a folded capillary cell for ζ-potential determination, performing the measurements at 25 °C.

# S26. Intensity-weighted nanoparticle size distributions and correlation functions

**NP4Aa**

**NP4Ab**

**NP4Bb**

**NP6A**

**NP6B**

**NP8B**

**NP-PLGA-PEG**

**NP-PLGA**

**NP-PLGA alt**

**DNP4Aa**

**DNP4Ab**

**DNP4Bb**

**DNP6A**

**DNP6B**

**DNP8B**

**DNP-PLGA-PEG**

**DNP-PLGA**

**DNP-PLGA alt**

# S27. Quantitation of dexamethasone

Analytical curves were constructed measuring the absorption of standard aqueous solutions of dexamethasone (25, 20, 15, 10, 5, 1, 0.5 and 0 µg/mL) at 240 nm using a quartz cuvette. Limits of detection (LOD) and quantification (LOQ) were calculated as 3.3 σ/S and 10 σ/S, respectively, where σ is the standard deviation of intercept and S is the slope of the calibration plot. The measurements were carried-out in triplicate for the quantitation of dexamethasone in the various experiments. Samples were subjected to dilution prior to measurement, in the event of absorbances larger than 1.

# S28. Transmission Electron Microscopy (TEM)

Samples for TEM have been prepared by sonication of NP suspensions for 60 sec followed by syringe filtration through 5.0 µm syringe filters. The resulting suspension (10 µL) was then deposited onto Formvar/Carbon supported copper grids 200 mesh and allowed to adsorb for 3 min before removing the excess sample with filter paper. The grids were left to dry on room temperature for 24 h before analysis. Imaging was performed using TEM at 80 kV with minimum dose exposure system.

# S29. ζ-potential Analysis During Storage at Room Temperature

Suspensions **DNP4Aa**, **DNP4Ab** and **DNP4Bb** have been stored at room temperature, and have been subjected to ζ-potential measurement in water at 25 °C weekly. The results are plotted in Figure S4.

**Figure S4**. ζ-potential of **DNP4Aa**, **DNP4Ab** and **DNP4Bb** over 4 weeks of storage at room temperature, in the absence of mechanical stirring.

#

# S30. References

(1) Stayshich, R. M.; Meyer, T. Y. New Insights into Poly(Lactic- Co -Glycolic Acid) Microstructure: Using Repeating Sequence Copolymers to Decipher Complex NMR and Thermal Behavior. *J. Am. Chem. Soc.* **2010**, *132* (31), 10920–10934. https://doi.org/10.1021/ja102670n.

(2) Stayshich, R. M.; Meyer, T. Y. Preparation and Microstructural Analysis of Poly(Lactic-Alt-Glycolic Acid). *J. Polym. Sci. Part A Polym. Chem.* **2008**, *46* (14), 4704–4711. https://doi.org/10.1002/pola.22801.
